# Construction of a stromal cell-related prognostic signature based on a 101-combination machine learning framework for predicting prognosis and immunotherapy response in triple-negative breast cancer

**DOI:** 10.3389/fimmu.2025.1544348

**Published:** 2025-05-14

**Authors:** Fanrong Li, Congnan Jin, Yacheng Pan, Zheng Zhang, Liying Wang, Jieqiong Deng, Yifeng Zhou, Binbin Guo, Shenghua Zhang

**Affiliations:** ^1^ Department of Genetics, School of Basic Medical Sciences, Suzhou Medical College of Soochow University, Suzhou, China; ^2^ Jiangsu Clinical Medicine Research Institute, The First Affiliated Hospital of Nanjing Medical University, Nanjing, China

**Keywords:** triple-negative breast cancer, machine learning, prognosis, immunotherapy, tumor microenvironment

## Abstract

**Background:**

Triple-negative breast cancer (TNBC) is a highly aggressive subtype with limited therapeutic targets and poor immunotherapy outcomes. The tumor microenvironment (TME) plays a key role in cancer progression. Advances in single-cell transcriptomics have highlighted the impact of stromal cells on tumor progression, immune suppression, and immunotherapy. This study aims to identify stromal cell marker genes and develop a prognostic signature for predicting TNBC survival outcomes and immunotherapy response.

**Methods:**

Single-cell RNA sequencing (scRNA-seq) datasets were retrieved from the Gene Expression Omnibus (GEO) database and annotated using known marker genes. Cell types preferentially distributed in TNBC were identified using odds ratios (OR). Bulk transcriptome data were analyzed using Weighted correlation network analysis (WGCNA) to identify myCAF-, VSMC-, and Pericyte-related genes (MVPRGs). A consensus MVP cell-related signature (MVPRS) was developed using 10 machine learning algorithms and 101 model combinations and validated in training and validation cohorts. Immune infiltration and immunotherapy response were assessed using CIBERSORT, ssGSEA, TIDE, IPS scores, and an independent cohort (GSE91061). FN1, a key gene in the model, was validated through qRT-PCR, immunohistochemistry, RNA interference, CCK-8 assay, apoptosis assay and wound-healing assay.

**Results:**

In TNBC, three stromal cell subpopulations—myofibroblastic cancer-associated fibroblasts (myCAF), vascular smooth muscle cells (VSMCs), and pericytes—were enriched, exhibiting high interaction frequencies and strong associations with poor prognosis. A nine-gene prognostic model (MVPRS), developed from 23 prognostically significant genes among the 259 MVPRGs, demonstrated excellent predictive performance and was validated as an independent prognostic factor. A nomogram integrating MVPRS, age, stage, and tumor grade offered clinical utility. High-risk group showed reduced immune infiltration and increased activity in tumor-related pathways like ANGIOGENESIS and HYPOXIA, while low-risk groups responded better to immunotherapy based on TIDE and IPS scores. FN1, identified as a key oncogene, was highly expressed in TNBC tissues and cell lines, promoting proliferation and migration while inhibiting apoptosis.

**Conclusion:**

This study reveals TNBC microenvironment heterogeneity and introduces a prognostic signature based on myCAF, VSMC, and Pericyte marker genes. MVPRS effectively predicts TNBC prognosis and immunotherapy response, providing guidance for personalized treatment. FN1 was validated as a key oncogene impacting TNBC progression and malignant phenotype, with potential as a therapeutic target.

## 1 Introduction

Breast cancer is the most commonly diagnosed cancer among women worldwide and remains one of the leading causes of cancer-related mortality in women (1). According to International Agency for Research on Cancer (IARC), the global incidence of breast cancer reached 2.3 million new cases in 2022 (2). Breast cancer is characterized by significant heterogeneity at both pathological and molecular levels, leading to differences in pathogenesis, risk factors, therapeutic responses, and prognosis (3). Among breast cancer subtypes, TNBC is a highly aggressive form characterized by the lack of estrogen receptor (ER), progesterone receptor (PR), and human epidermal growth factor receptor 2 (HER2), accounting for approximately 15-20% of all breast cancer cases (4). Moreover, TNBC exhibits a high degree of molecular heterogeneity and can be further classified into multiple molecular subtypes based on gene expression profiles, each with distinct biological characteristics, disease progression patterns, and treatment sensitivities (4, 5). Compared to other subtypes, TNBC is often associated with a higher histological grade, a strong tendency for recurrence and metastasis, and the absence of effective therapeutic targets, limiting treatment options to surgery, chemotherapy, and radiotherapy (6). For advanced or metastatic TNBC patients, however, chemotherapy is usually insufficient, with most patients eventually developing chemoresistance. The development of chemoresistance in TNBC patients may be closely linked to the tumor microenvironment (TME) (7).

Advances in single-cell transcriptomics have provided a valuable tool for studying the tumor microenvironment. The TME is composed of tumor cells, stromal cells, immune cells, secreted factors, extracellular matrix, and other components (8), all of which are critical to cancer initiation and progression (9). These components interact through signalling pathways and cell-cell interactions to influence tumor progression and treatment responses (10). Recent studies have highlighted the importance of stromal cells in tumor progression, particularly myCAF, VSMC, and pericyte (11–17). Myofibroblasts play multifaceted roles within tumors, primarily supporting tumor growth and immune suppression. They provide support to tumor cells by secreting growth factors and ECM proteins, thereby promoting tumor proliferation and migration. Myofibroblasts also suppress antitumor immune responses by secreting immunosuppressive factors such as TGF-β and IL-11, and through metabolic regulation, rendering the TME immunosuppressive (18). VSMC normally maintain vasoconstriction and regulate blood flow. However, under pathological conditions, they undergo phenotypic switching to a proliferative and synthetically active state, contributing to vascular remodelling and neointimal hyperplasia (19). This phenotypic change parallels tumor behavior, as VSMC within the TME can promote tumor angiogenesis and ECM remodeling, thus providing support for tumor growth and nutrient supply, and playing a key role in tumor progression. Pericytes are also known to contribute to tumor angiogenesis and the establishment of an immunosuppressive environment (13, 20). Under physiological conditions, pericytes are connected to endothelial cells through N-cadherin and local adherent plaques, which facilitate intercellular signaling. This interaction helps maintain vascular wall homeostasis and ensures the normal function of the microcirculation (21). Moreover, most pericyte-endothelial cell communication relies on a paracrine mechanism. For example, endothelial cells promote pericyte recruitment and coverage through the PDGF-B/PDGFR-β axis, maintaining vascular stability and regulating vascular maturation (22, 23). However, in the tumor microenvironment, the interaction between pericytes and ECs undergoes significant changes, leading to vascular abnormalities. For example, Perrot found that the elevated concentration of prostaglandin E2 (PGE2) in the TME downregulates N-cadherin through the prostaglandin E receptor EP-4 and EP-1 pathways. This weakens pericyte-EC adhesion, disrupts their interactions, and destroys vascular barrier integrity, thereby facilitating tumor cell entry into the circulatory system and promoting the development of hematogenous metastasis in malignant tumors (21). Zhang et al. found that the expression of Rho guanine nucleotide exchange factor 37 (ARHGEF37) is increased in hepatocellular carcinoma (HCC), directly activating Cdc42 in cancer cell pseudopodia. This promotes extrahepatic invasion of HCC cells, disrupts endothelial-pericyte interactions, and ultimately contributes to the development of HCC lung metastasis (24). Additionally, Yi Lu et al. found that in HCC, hypoxia-induced PDGF-B production activates hepatic stellate cells (specialized pericytes in the liver), enabling their interaction with endothelial cells and thereby promoting tumor angiogenesis (25). These aberrant vessels generate hypoxic microenvironments, promoting immune tolerance and reducing the effectiveness of chemotherapy (13, 26). These stromal cells collectively regulate tumor invasion, angiogenesis, and immune suppression within the TME, impacting TNBC prognosis and response to immunotherapy. Therefore, marker genes associated with myCAF, VSMC, and pericyte may have significant roles in predicting prognosis and immunotherapeutic response in patients. However, there remains a lack of systematic studies focusing on stromal cell-related genes in TNBC.

Given the strengths of bulk RNA-seq in capturing larger sample sizes and extensive clinical information, combined with the high resolution of single-cell transcriptomics, an increasing number of studies are integrating both approaches to analyze tumor heterogeneity (27), develop risk models, and construct nomograms to predict patient outcomes, providing new clinical treatment methods for cancer (28). In this study, we obtained scRNA-seq data, microarray data, and RNA-seq data for TNBC patients from the GEO and The Cancer Genome Atlas (TCGA) databases. We first conducted a comprehensive analysis of the scRNA-seq data to map the TME and identify marker genes for cell populations. Using single-sample gene set enrichment analysis (ssGSEA) and WGCNA, we identified 259 marker genes associated with myCAF, VSMC, and pericytes. Subsequently, we employed a novel machine learning framework involving 10 machine learning algorithms and 101 model combinations to construct a consensus MVPRS. We validated the prognostic value of the model in both training and validation cohorts through Kaplan-Meier survival and area under the curve (AUC) analyses.

**Figure 1:**
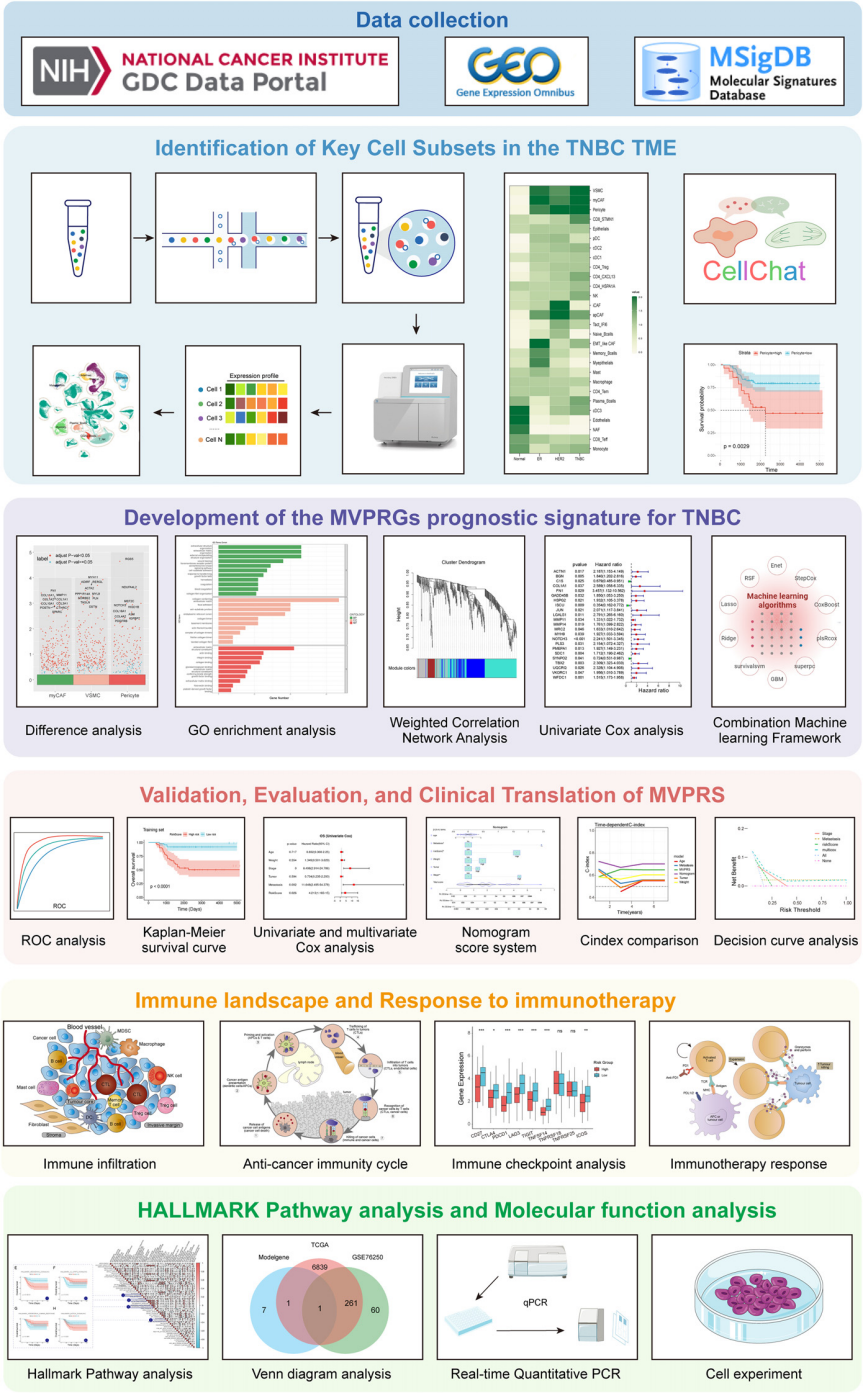
Flowchart of this study. This study workflow is based on multi-omics data integration analysis, aiming to explore key cell subpopulations in the TNBC microenvironment, identify their characteristic genes, construct a related prognostic signature, and comprehensively evaluate its clinical value, immune microenvironment association, immunotherapy response, and functional pathways. The research workflow consists of the following six main modules: Module 1 (Data Collection): RNA-seq, scRNA-seq data, and gene sets were obtained from the TCGA, GEO, and MSigDB databases to provide data support for subsequent analyses. Module 2 (Identification of Key Cell Subpopulations): This module analyzes key cell subpopulations in the TNBC microenvironment using single-cell transcriptome data. By integrating cell communication analysis and prognostic analysis, it identifies cell types with potential biological functions. Module 3 (MVPRS Prognostic Model Construction): Differential analysis, weighted gene co-expression network analysis (WGCNA), and univariate Cox analysis were integrated to identify characteristic genes of key cell subpopulations. Machine learning algorithms were then used to construct the MVPRS prognostic signature model. Module 4 (Clinical Value Evaluation of MVPRS): The application value of MVPRS in TNBC prognosis prediction was evaluated through ROC curves, Kaplan-Meier survival analysis, cox regression analysis, and nomogram scoring system. Its clinical applicability was compared by using C-index and validated by using decision curve analysis (DCA). Module 5 (Relationship Between MVPRS, Immune Infiltration, and Immunotherapy Response): The role of MVPRS in immune infiltration, the anti-tumor immune cycle, immune checkpoint gene expression, and immunotherapy response was analyzed to assess its potential value in tumor immune regulation. Module 6 (Mechanistic Exploration of MVPRS): HALLMARK pathway analysis, Venn diagram-based key gene screening, and molecular and cellular experiments were combined to further validate the functions of MVPRS-related genes and reveal their potential mechanisms.

Furthermore, we investigated the relationship between MVPRS and immune infiltration status, as well as response to immune checkpoint inhibitor (ICI), exploring potential biological processes associated with poor prognosis in TNBC linked to MVPRS. We also developed a nomogram incorporating clinical pathological features to assist in predicting patient outcomes in clinical practice. By constructing this model, our study provides a theoretical foundation for understanding the pathophysiology of TNBC and offers opportunities for personalized prognosis prediction and immunotherapy. The overall workflow of the study is illustrated in **Figure 1**.

## 2 Methods and materials

### 2.1 Clinical sample collection

TNBC tissue samples and corresponding pair-matched adjacent normal tissue samples, which were obtained from patients underwent tylectomies at the Affiliated Hospital of Soochow University, were snap-frozen in liquid nitrogen immediately after resection. Before surgery, none of these patients received anti-cancer treatment, including chemotherapy,radiotherapy or immunotherapy. This study was approved by the Ethics Committees of Soochow University.

### 2.2 Data collection

ScRNA-seq data of breast tissue were obtained from the GEO database, with accession number GSE161529. Additionally, two independent microarray datasets retrieved from the GEO database were GSE58812 and GSE76250. FPKM normalized transcriptomic tumor data of TNBC were obtained from the TCGA database. Furthermore, clinical information for 112 TNBC patients was downloaded from the UCSC Xena platform (https://xenabrowser.net/). To ensure data quality and model robustness, we performed rigorous data cleaning. Survival analysis (such as Cox regression and Kaplan-Meier analysis) relies on key variables like survival time and event status. Missing these essential key data could affect the reliability of the analysis and the accuracy of statistical inference. Therefore, we excluded samples with missing survival time or event status. Additionally, to prevent data duplication from interfering with model training, we removed duplicate samples, ensuring dataset independence and improving the model’s generalizability. During model training, we further evaluated the data distribution and found that only two patients had a survival time of less than one year, both of whom were alive, representing no event occurred. Since survival analysis relies on a sufficient number of events (eg: death) to compute hazard ratios (HR) and the C-index, the extremely small number of short-term survivors could compromise the model’s stability in 1-year survival prediction. To address this, we excluded samples with a follow-up time of less than 200 days, ensuring the model’s discriminative power and predictive accuracy across different time scales. Finally, we retained 104 TNBC samples from the GSE58812 dataset for constructing the prognostic feature model, while 96 TNBC samples from the TCGA-TNBC dataset were retained for model validation (**Supplementary Table S1**). Moreover, we collected transcriptomic data and corresponding clinical information from patients treated with anti-PD1 and anti-CTLA4 therapies from the GEO database (accession number GSE91061) to explore the value of MVPRS in predicting immunotherapy response.

### 2.3 Single-cell RNA-seq data processing

We analyzed the scRNA-seq data using the R package Seurat (version 4.3.0) (29). To perform high-quality filtering, we removed genes detected in fewer than three cells and filtered cells by selecting those with gene counts between 100 and 6000, mitochondrial RNA content below 50%, and hemoglobin proportion (percent_hb) less than 5%. Subsequently, all mitochondrial and ribosomal genes were removed (**Supplementary Figure S1**). The data were then normalized using the Normalization function. Highly variable genes were selected using the FindVariableGenes function, setting the nFeature parameter to 3000, based on average expression and dispersion thresholds. These highly variable genes were then scaled using the ScaleData function, followed by principal component analysis (PCA) using the RunPCA function. Graph-based Louvain clustering was performed on the top 20 principal components (PCs) using the FindClusters function, with a clustering resolution parameter (Res) set to 1.0. The FindAllmarker function was used with the Wilcoxon test and Bonferroni correction for p-values to identify cluster-specific marker genes. Subsequently, cell identities were authenticated based on known cell marker genes. T cells were identified using CD3D and CD3E as marker genes, while NK cells were identified using NCAM1, GNLY, NKG7, and KLRD1. Stromal cells were identified based on DCN, COL1A1, COL1A2, LUM, PDGFRA, and PDGFRB, whereas Plasma B cells were identified using MZB1 and IGHG4. Myoepithelial cells were identified using KRT14 and ACTA2, and Myeloid cells were identified based on CD68, CD163, LYZ, SPP1, CST3, LST1, C1QC, C1QA, and TREM2. Epithelial cells were identified using CD24, EPCAM, KRT19, KRT7, KRT8, and KRT18, while Endothelial cells were identified using PECAM1, VWF, ENG, CDH5, and PLVAP. Finally, CD20^+^ B cells were identified based on MS4A1 and CD79A. Uniform Manifold Approximation and Projection (UMAP) was applied for visualization. For annotating cell subpopulations, we used the same dimensionality reduction and clustering methods, while batch correction for “orig.ident” was performed using the Harmony package (version 1.2.1). The characteristic genes for each subcluster are provided in the **Supplementary Table S2**-**Supplementary Table S5**.

### 2.4 Single-sample gene set enrichment analysis

Single-sample Gene Set Enrichment Analysis (ssGSEA) is a widely used method for quantifying the enrichment of a specific gene set within an individual sample. The ssGSEA score for each sample reflects the degree of systematic upregulation or downregulation of a particular gene set within that sample. In this study, to identify marker genes of different cell types in the tumor microenvironment (TME), we used the FindAllMarkers function in the Seurat package to calculate highly expressed genes for each cell cluster. For each cluster, we selected genes with p_value < 0.05 and the top 100 ranked by avg_log2FC as marker genes. These marker genes were then used in the ssGSEA method to calculate the abundance of these cell types in each TNBC sample. Next, we used the surv_categorize function to group patients based on the optimal cutoff value and devided them into high-risk and low-risk groups. Kaplan-Meier (KM) survival analysis was then performed using the survminer package, and the log-rank test was applied to compare survival curves between the high and low abundance groups to determine the statistical significance of survival differences between the two groups.

Additionally, with the same method, we calculated the MVP(myCAF-VSMC-Pericyte-marker genes) score for each TNBC sample by using the marker genes of myCAF, VSMC, and Pericytes as the gene set.

### 2.5 The tissue distribution of TME cell subsets

The OR can be used to quantify the relative enrichment of a specific cell cluster in a particular tissue (30). Specifically, we used 2×2 contingency tables and performed Fisher’s exact test to assess differences in the abundance of each cell type across different subtypes, and calculated odds ratios (ORs) to reflect the direction and magnitude of these abundance differences. Meanwhile, the Benjamini-Hochberg method was applied to adjust for multiple testing and effectively control the false discovery rate (FDR). An OR value greater than 1.5 indicates that the cell cluster tends to be distributed in the specific tissue, while an OR value less than 1.5 suggests a lower tendency for distribution in that tissue. To describe the distribution preferences of cell clusters across different tissues, we used the OR as the measurement metric.

### 2.6 Cell-cell communication analysis

The R package CellChat is used to infer, visualize, and analyze cell-cell communication within scRNA-seq data, enabling the description of interactions between ligands, receptors, and their cofactors. This method leverages a curated ligand–receptor interaction database and applies a mass action law-based model to compute communication probabilities. The calculation is based on the product of ligand gene expression in sender cells and receptor gene expression in receiver cells, simulating the binding dynamics of ligand–receptor interactions. To enhance confidence in the communication inference, CellChat performs permutation testing for each ligand–receptor pair to exclude false positives due to random expression, yielding statistically significant *p*-values. To explore the interaction frequency and intensity among different cell types in the microenvironment of TNBC patients, as well as potential communications between these cell types, we used CellChat to analyze the ligand-receptor interactions among different cells.

### 2.7 Gene ontology enrichment analysis

To explore the biological functions associated with specific cell clusters or gene sets, we conducted Gene Ontology (GO) enrichment analysis. GO enrichment analysis includes three aspects: Biological Processes (BP), Molecular Functions (MF), and Cellular Components (CC), which are used to systematically identify significantly enriched gene functional categories (31). We referred to the methodological study by Yu et al., 2012 (32), and selected the R package clusterProfiler to perform GO enrichment analysis on the target gene set. Specifically, we first used the bitr() function to convert gene symbols (SYMBOL) to ENTREZ IDs to ensure compatibility with the GO database. Then, we conducted enrichment analysis using the enrichGO() function, covering the BP, MF, and CC categories. The p-value threshold was set at 0.05, and the q-value threshold was also set at 0.05, with Benjamini-Hochberg (BH) correction applied to control the false discovery rate (FDR) and ensure statistical reliability. During the result processing stage, we used the setReadable() function to convert ENTREZ IDs back to gene symbols to enhance the biological interpretability of the results. Additionally, we utilized barplot(), dotplot(), and ggplot() functions for the visualization of enrichment analysis results, clearly presenting the enrichment characteristics of key functional pathways.

### 2.8 Gene set enrichment analysis

We used the ssGSEA method from the R package GSVA to calculate the MVP score for each TNBC sample, and classified the samples into high-risk and low-risk groups based on the median value of all samples’ scores. Subsequently, differential gene analysis between the high-risk and low-risk groups was conducted using the limma package. To identify potential signaling pathways associated with this feature, we calculated the GSEA scores for 50 tumor-related signaling pathways. Furthermore, to reveal the biological processes (BP) involved in the different risk subgroups, we used the R package clusterProfiler to perform GSEA analysis on the GO_BP gene sets (c5.go.v7.5.1.symbols.gmt) between the two risk groups. Annotation information for Hallmark and GO_BP gene sets can be downloaded from the MSigDB database (https://www.gsea-msigdb.org/gsea/msigdb/index.jsp). The database version is v2024.1, updated in August 2024. It remains widely used in biological research and continuously provides authoritative reference data for gene set enrichment analysis. This study is based on the latest gene set information provided by MSigDB to ensure the accuracy and timeliness of signaling pathway analysis.

### 2.9 Weighted correlation network analysis

WGCNA is a systematic biological approach used to characterize gene association patterns across different samples, effectively identifying highly co-expressed gene sets (33, 34). In this study, we utilized the ssGSEA algorithm to calculate the MVP activity score for each TNBC sample and used it as the phenotypic data for WGCNA analysis. To identify co-expression modules significantly associated with MVP scores, we applied WGCNA to TNBC microarray gene expression data. Specifically, we first selected highly variable genes to remove low-expression or low-variance genes that could affect network construction. Next, we ensured data quality by computing the clustering relationships among samples, identifying and then removing samples with potential outliers. After removing abnormal samples, we proceeded with network construction and module identification. To determine the optimal soft threshold, we used the pickSoftThreshold function and set β = 3 to ensure that the network conformed to the scale-free topology. We then constructed a weighted gene co-expression network, calculated gene similarity, and applied hierarchical clustering to group genes into different co-expression modules. To refine module identification, we further used the dynamic tree-cutting algorithm for module segmentation and merged them based on module similarity, ultimately identifying four major modules. Finally, we conducted module-phenotype association analysis by calculating the correlation between each module and the MVP score, selecting the module with the highest correlation for further analysis.

### 2.10 Construction of prognostic signature by integrative machine learning approaches

We applied univariate Cox regression analysis to the GSE58812 dataset to identify MVP genes with potential prognostic significance. Using GSE58812 as the training set and TCGA-TNBC as the validation set, we constructed a survival prediction model. A total of ten machine learning methods were employed, covering linear Cox regression, regularization techniques, ensemble learning, dimensionality reduction, and nonlinear modeling, ensuring model comprehensiveness and robustness. These methods included Lasso, Ridge, stepwise Cox, CoxBoost, random survival forest (RSF), elastic net (Enet), partial least squares regression Cox (plsRcox), supervised principal components (SuperPC), generalized boosted regression modeling (GBM), and survival support vector machine (survival-SVM). Among them, Lasso, Ridge, and Elastic Net were primarily used for feature selection and dimensionality reduction in high-dimensional data. CoxBoost, RSF and GBM enhanced predictive performance through ensemble learning. As dimensionality reduction methods, SuperPC and plsRcox reduced noise, while survival-SVM identified complex nonlinear patterns. This multi-method integration strategy contributes to optimizing feature selection, improving model generalization, and enhancing survival prediction accuracy.

In the training set, we applied 101 different combinations of the ten algorithms and performed feature selection and model construction within a 10-fold cross-validation framework. We evaluated all constructed models by calculating their C-index in both the training and validation sets. The models were ranked based on their average C-index for predictive performance, and we ultimately selected the algorithm combination that demonstrated robust performance and clinical translational potential. Based on this selection, we used CoxBoost + Elastic Net for feature selection and risk signature modeling. First, we optimized the penalty parameter of CoxBoost using the optimCoxBoostPenalty function and determined the optimal number of boosting steps (stepno) through cross-validation (cv.CoxBoost) to identify survival-related variables. The model generated a set of non-zero coefficients, indicating that these variables were significantly associated with survival outcomes and were selected for further analysis. Next, we applied Elastic Net (α = 0.5) for further variable selection. The optimal regularization parameter (lambda = 0.06943276) was determined through cross-validation, extracting significant variables and ultimately constructing a multivariable Cox proportional hazards model. This led to the identification of a prognostic signature for predicting overall survival in TNBC patients, referred to as the MVP-related signature (MVPRS).

### 2.11 Survival analysis and construction of a predictive nomogram

Based on the median MVPRS risk score, we divided the samples in both the training and validation sets into high-risk and low-risk groups. Subsequently, we used the R package survminer to perform Kaplan-Meier curve analysis to assess the differences in OS between the high-risk and low-risk groups (p < 0.05). Additionally, the timeROC package was used for ROC curve analysis to evaluate the predictive sensitivity and specificity of MVPRS for OS in TNBC patients. However, during model training, we observed that only two patients had an overall survival of less than one year, and both were censored (i.e., no events occurred). This data distribution could affect model stability and reduce the accuracy of short-term survival predictions. Since timeROC analysis relies on an adequate number of short-term survival events to ensure effective differentiation between positive and negative cases, the current dataset was insufficient for reliable ROC calculations. Therefore, we selected 3-year, 5-year, and 7-year time points for analysis and compared the area under the curve (AUC) of MVPRS with other clinical characteristics. We also performed univariate and multivariate Cox regression analyses on the TCGA-TNBC dataset to determine whether MVPRS is an independent prognostic factor for the survival of TNBC patients. To enhance the prognostic accuracy and predictive capability of the model, we constructed a nomogram that integrates MVPRS and clinical characteristics to quantify the expected survival of TNBC patients. Finally, the discrimination and accuracy of the nomogram were evaluated using ROC curves and the C-index, and decision curve analysis (DCA) was used to assess its net clinical benefit.

### 2.12 Analysis of the association between MVPRS and cell infiltration in the TNBC tumor microenvironment

The tumor microenvironment (TME) refers to the surrounding microenvironment in which tumor cells exist. It is primarily composed of tumor cells and various cellular components, including immune cells, tumor-associated fibroblasts, bone marrow-derived inflammatory cells, stromal tissue, and blood vessels. Additionally, it contains signaling molecules such as cytokines, chemokines, and the extracellular matrix (ECM). This complex ecosystem plays a critical role in tumor initiation, progression, immune evasion, and therapeutic response. To assess the association between MVPRS and cell infiltration in the TME of TNBC, we used ssGSEA to calculate the characteristic scores of TME cells in TNBC patients. To validate the reliability of the ssGSEA results, we further applied the CIBERSORT algorithm to quantitatively analyze the infiltration levels of 22 types of immune cells (35).

### 2.13 Obtaining anti-cancer immunity cycle scores

The anti-cancer immunity cycle is a key component of cancer immunotherapy, involving seven steps: the release of cancer antigens (Step 1), cancer antigen presentation (Step 2), initiation and activation of the immune response (Step 3), trafficking of immune cells to the tumor (Step 4), infiltration of immune cells into the tumor (Step 5), recognition of cancer cells by T cells (Step 6), and killing of cancer cells (Step 7) (36). These seven steps together constitute the anti-cancer immunity cycle. We obtained activity scores for these seven anti-cancer immune steps for the TCGA-TNBC samples from the Tracking Tumor Immunophenotype (TIP) platform (http://biocc.hrbmu.edu.cn/TIP/index.jsp).

### 2.14 TIDE analysis and evaluation of ICI therapy response

We used the Tumor Immune Dysfunction and Exclusion (TIDE) analysis method to evaluate tumor immune escape mechanisms and predict responses to ICI therapy (37). Gene expression data from patients were input into the TIDE online platform to assess their immune escape scores and further explore the association with MVPRS. We used the Immunophenoscore (IPS) algorithm to predict the response to ICI therapy. This algorithm calculates the IPS score using machine learning based on unbiased gene expression of representative cell types, with higher IPS scores indicating a better response to immunotherapy. The IPS scores for the TCGA-TNBC patient samples were obtained from the Cancer Immunome Atlas (TCIA) database (https://tcia.at/home).

### 2.15 RNA extraction and Quantitative real-time polymerase chain reaction

Total RNA was extracted using an RNA extraction reagent (RC101-01, Vazyme) following the manufacturer’s instructions. The concentration and purity of the extracted RNA were measured using a NanoDrop 2000 spectrophotometer. Subsequently, total RNA was reverse transcribed into cDNA using a reverse transcription kit (R312-01, Vazyme) according to the reaction conditions specified in the kit’s protocol. Real-time quantitative PCR (RT-qPCR) was performed using a SYBR Green dye system (Q111-02, Vazyme) on an ABI 7500 Fast Real-Time PCR System (Applied Biosystems, USA). The primers used for qRT-PCR were as follows:

*FN1*: Forward primer: 5’-GCTGCACATTGCCTGTTCTG-3’, Reverse primer: 5’-TCCTACAGTATTGCGGGCCA-3’.

*GAPDH*: Forward primer: 5’-GATTCCACCCATGGCAAATTC-3’, Reverse primer: 5’-CTGGAAGATGGTGATGGGATT-3’.

### 2.16 Cell cultures

The TNBC cell line MDA-MB-231 was purchased from Wuhan PriCellaLife Technologies and authenticated using short tandem repeat (STR) profiling. The cells were cultured in high-glucose DMEM (PM150210, Pricella) supplemented with 10%(v/v) fetal bovine serum (16000044, Gibco™) and 1%(v/v) penicillin-streptomycin solution (10 kU/mL penicillin and 10 mg/mL streptomycin, PB180120, Pricella). All cells were maintained at 37°C in a 5%(v/v) CO_2_ incubator. The culture medium was replaced regularly, and cells were passaged using 0.25% trypsin-EDTA(w/v) solution (25200056, Invitrogen).

### 2.17 RNA interference

The small interfering RNAs (siRNAs) used in the experiment were synthesized by AnZhen BioMed (Suzhou) and specifically targeted *FN1*. Transfections were performed using Lipofectamine™ 3000 Transfection Reagent (L3000015, Thermofisher) according to the manufacturer’s protocol, with a final siRNA concentration of 75 nM. After transfection, cells were cultured for the specified duration based on experimental requirements to assess gene knockdown efficiency. The siRNA target sequences for *FN1* used in this study were as follows:

si*FN1*#1:

Sense: 5’-CUGCUCCAAGAAUUGGUUUTT-3’

Antisense: 5’-AAACCAAUUCUUGGAGCAGTT-3’

si*FN1*#2:

Sense: 5’-CAGCACAACUUCGAADUAUTT-3’

Antisense: 5’-AUAAUUCGAAGUUGUGCUGTT-3’

si*FN1*#3:

Sense: 5’-UGGUUGUAUCAGGACUUAUTT-3’

Antisense: 5’-AUAAGUCCUGAUACAACCATT-3’

NC:

Sense: 5’-UUCUCCGAACGUGUCACGUTT-3’

Antisense: 5’-ACGUGACACGUUCGGAGAATT-3’.

### 2.18 Immunohistochemistry

Paraffin-embedded tissue samples were sectioned into 4 μm thick slices. The slices underwent antigen retrieval by heating in a pressure cooker for 15 ~ 20 minutes with 0.01 M citrate buffer to break aldehyde bonds formed during fixation. The samples were then incubated overnight at 4°C with a specific FN1 antibody (1:500, GB15091, Servicebio). The next day, the sections were incubated with an HRP-conjugated secondary antibody (goat anti-mouse IgG-HRP, G1214, Servicebio) at room temperature for 30 minutes, followed by staining with DAB chromogenic reagent (K5007, Dako) according to the manufacturer’s instructions. Finally, the sections were observed and photographed under a microscope.

### 2.19 Cell proliferation assay

Cell proliferation was assessed using the Cell Counting Kit-8 (K1018, APExBIO). The cells were seeded at a density of 1000 cells per well in a 96-well plate, with 100 µL of complete culture medium added to each well. Absorbance at 450 nm was measured at 0 and 48 hours using SpectraMax^®^ iD5 (Molecular devices, USA). One hour before measurement, 10 µL of CCK-8 reagent was added to each well and mixed thoroughly. Three replicates were performed for each sample.

### 2.20 Wound-healing assay

We assessed cell migration ability using a wound healing assay. MDA-MB-231 cells transfected with FNI siRNA or NC siRNA were seeded in 6-well plates. Once the cells reached 90% confluence, a sterile 200 µL pipette tip was used to create a clean, straight scratch in the cell monolayer at the center of each well. For data integration and collection, the wound healing process was observed at 0 and 48 hours, and the gap between the edges of the wound was evaluated by randomly selecting three fields of view under an inverted microscope. Wound width and healing were analyzed using ImageJ software(version 1.52). Each experiment was repeated three times.

### 2.21 Flow cytometry analysis of apoptosis

Cell apoptosis was analyzed using Annexin V-fluorescein isothiocyanate (FITC)/propidium iodide (PI) double staining. MDA-MB-231 cells were collected 48 hours after siRNA treatment, washed twice with pre-chilled PBS, and then stained according to the instructions of the Annexin V-FITC/PI double staining kit (C1383L, Beyotime). The cells were resuspended in binding buffer and incubated with Annexin V-FITC and PI at room temperature for 15 minutes, protected from light. After staining, apoptosis was assessed using a flow cytometer (BD FACSCanto II), and the apoptotic cells were analyzed with FlowJo software(version 10.8.1).

### 2.22 Statistical analysis

All statistical analyses were performed using R software (version 4.3.0) and appropriate packages. The Wilcoxon test was used as a non-parametric method to estimate the differences between two non-normally distributed variables. Kaplan-Meier survival analysis and log-rank tests were conducted using the R package survival to compare OS between different subgroups. Spearman correlation analysis was used to examine the relationship between the risk score and immune cell infiltration. The qRT-PCR results were analyzed using Student’s t-test. Unless otherwise specified, statistical significance was set at *P* value < 0.05.

## 3 Results

### 3.1 The breast cancer microenvironment atlas

Based on scRNA-seq data from GSE161529, we obtained gene expression profiles for 204,332 cells from 34 surgical tissue samples from 34 patients. These samples included 13 normal tissues, 11 ER-positive subtype tumors, 6 HER2-positive subtype tumors, and 4 TNBC subtype tumors. After quality control to ensure good gene coverage, consistent read ranges, and low mitochondrial content, a total of 197,867 cells were retained for further analysis.

**Figure 2:**
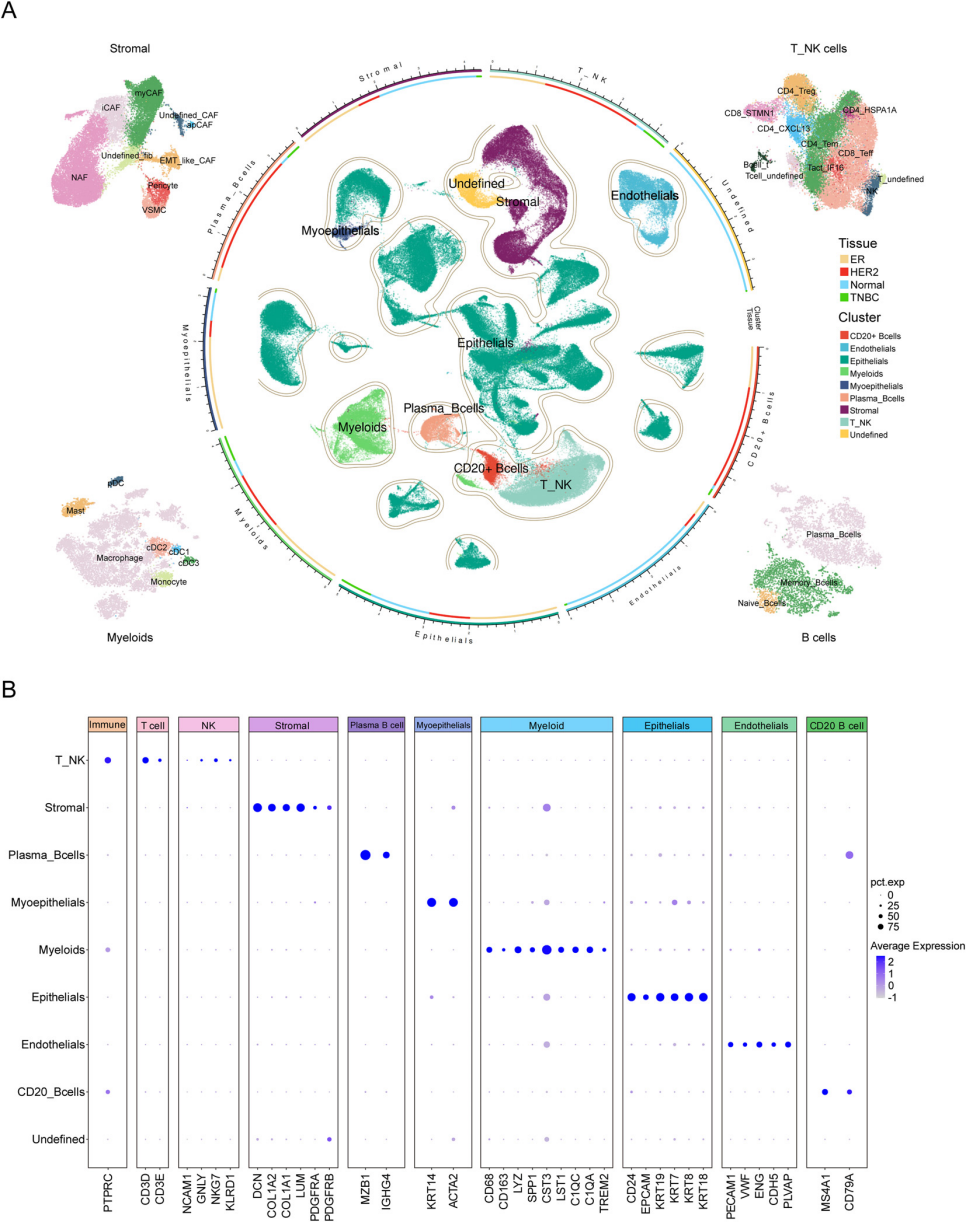
Comprehensive single-cell transcriptomic analysis of TME cell types in breast cancer. **(A)** UMAP visualization of single-cell RNA sequencing data from breast cancer samples, showing the distribution and clustering of distinct cell types within the tumor microenvironment. The central region displays the clustering distribution of major cell types, including Myeloid cells, T_NK cells, CD20+ B cells, Plasma B cells, Epithelial cells, Stromal cells, and Undefined cell populations. The segmented annotations in the outer ring indicate the classification of major cell types. The circular color bar represents the correspondence between cell types and sample tissue sources. Surrounding the plot from left to right are the subpopulation classifications of Stromal cells, T_NK cells, Myeloid cells and B cells. The color legend on the right indicates that “Tissue” is used to differentiate samples from different sources: ER, HER2, Normal, and TNBC, while “Cluster” is used to distinguish the clustering of major cell types. **(B)** The dot plot illustrates the expression levels of marker genes across major cell types in the single-cell RNA sequencing data. The X-axis represents the marker genes of each cell type, while the Y-axis denotes different cell types. The dot size (pct.exp) indicates the proportion of cells within each cluster expressing the gene, whereas the color intensity (Average Expression) reflects the gene’s average expression level.

First, we performed PCA on the top 3,000 variable genes for dimensionality reduction, followed by graph-based clustering to determine 48 cell clusters. These cell clusters were annotated using known marker genes. We identified eight major cell groups (**Figure 2A, B**, **Supplementary Figure S2A**), including epithelial cells (N = 115,168), stromal cells (N = 23,008), endothelial cells (N = 10,352), myoepithelials cells (N = 1,616), T/NK cells (N = 22,866), myeloid cells (N = 12,790), plasma B cells (N = 4,025), and CD20^+^ B cells (N = 3,584), as well as an additional unclassified cell population (N = 4,458). The heatmap of cell type-specific marker gene expression shows a clear gradient in transcriptional expression levels across different cell populations, further validating the accuracy of the cell annotation results (**Supplementary Figure S2B**).

Further subgroup annotation was performed on the T/NK cell groups, stromal cells, myeloid cells, and B cells. For T/NK cells, we identified a total of eleven subgroups, including CD4_CXCL13, CD4_HSPA1A, CD4_Tem, CD4_Treg, CD8_STMN1, CD8_Teff, Tact_IFI6, and NK cells, as well as two undefined subgroups and one subgroup co-expressing both T cell and B cell markers, which is likely to represent doublets (**Figure 2A**, **Supplementary Figure S2C**, **Supplementary Table S2**). Stromal cells were divided into nine subgroups, including myCAF, iCAF, apCAF, pericyte, VSMC, EMT-like CAF, NAF, and two undefined fibroblast subgroups (**Figure 2A**, **Supplementary Figure S2D**, **Supplementary Table S3**). Myeloid cells were classified into seven subgroups: macrophage, monocyte, cDC1, cDC2, cDC3, pDC, and mast cell (**Figure 2A**, **Supplementary Figure S2E**, **Supplementary Table S4**). B cells were subdivided into three subgroups, including plasma cells, memory B cells, and naive B cells (**Figure 2A**, **Supplementary Figure S2F**, **Supplementary Table S5**).

### 3.2 Identification of key cell types in TNBC based on cell distribution, communication and their prognostic associations

**Figure 3:**
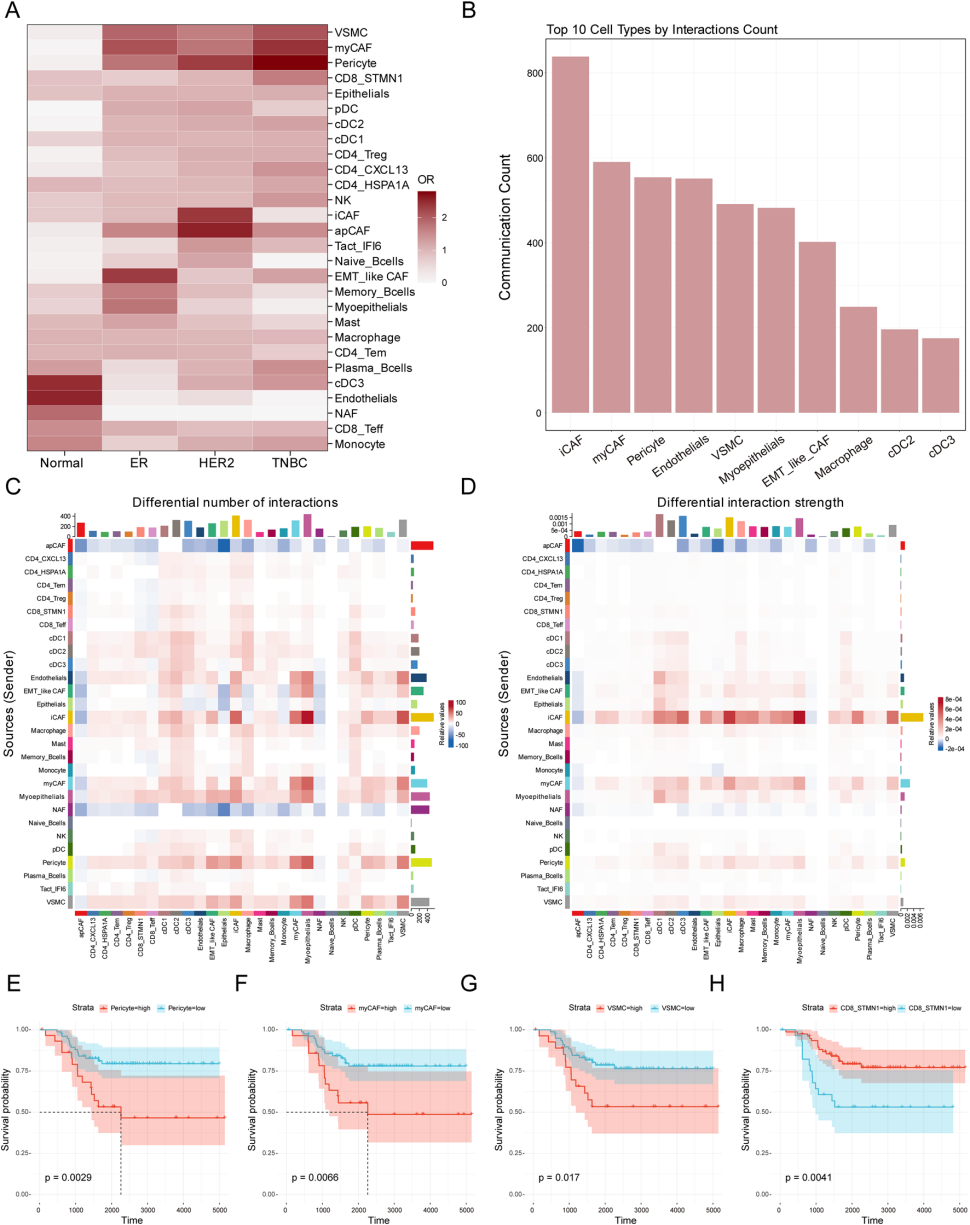
Identification of crucial cell types in the TME of TNBC. **(A)** Heatmap showing distribution preferences of cell types across different tissues in the breast cancer. The X-axis represents different breast cancer subtypes, while the Y-axis represents different cell types. The color bar indicates the OR value, and the color intensity reflects the abundance differences of various cell types across the four tissue types. **(B)** The bar chart displays the 10 most active cell types in cell communication within the tumor microenvironment. The X-axis shows the top 10 cell types ranked by total communication events, while the Y-axis represents the total number of cell-cell communication in the tumor microenvironment. **(C)** The heatmap illustrates changes in cell-cell communication frequency in TNBC tissue compared to normal tissue. The X-axis represents signal-receiving cell types, while the Y-axis represents signal-sending cell types. The color indicates the changing trend of cell interactions: red signifies an increase in communication frequency in TNBC compared to normal tissue for a specific cell type, whereas blue indicates a decrease. The colored bar chart at the top represents the total signal input for each receiving cell type, while the colored bar chart on the right represents the total signal output for each sending cell type. **(D)** The heatmap illustrates changes in cell-cell communication intensity in TNBC tissue compared to normal tissue. The X-axis represents signal-receiving cell types, while the Y-axis represents signal-sending cell types. The color indicates the trend in interaction intensity: red signifies an enhanced signal transmission effect in TNBC compared to normal tissue for a specific cell type, whereas blue indicates a weakened effect. The colored bar chart at the top represents the total signal input for each receiving cell type, while the colored bar chart on the right represents the total signal output for each sending cell type. **(E-H)** The Kaplan-Meier survival curves evaluate the prognostic significance of myCAF, VSMC, Pericytes, and CD8_STMN1 cell abundance in TNBC patients. The X-axis represents survival time from diagnosis (in days), while the Y-axis indicates the probability of patients remaining alive at a given time point. The Strata grouping is represented by red and blue curves, which correspond to patient groups with high and low abundance of the respective cell type. The shaded area denotes the 95% confidence interval (CI) for survival probability.

The composition of the tumor microenvironment varied significantly across tissues from different patients. To distinguish the microenvironmental differences between TNBC and normal tissues as well as other breast cancer subtypes, we analyzed the tissue distribution characteristics of the previous 28 clearly defined cell populations. The OR analysis revealed that VSMC, myCAF, pericyte, CD8_STMN1, cDC2, CD4_CXCL13, and NK cells showed significant preferential distribution in TNBC. In contrast, cDC3, endothelial cells, NAF, CD8_Teff, and monocyte were primarily enriched in normal tissues. ER-positive samples were enriched with EMT-like CAF, memory B cells, myoepithelial cells, and mast cells, whereas iCAF, apCAF, Tact_IFI6, and naive B cells were mainly found in HER2-positive tumor samples (**Figure 3A** and **Supplementary Table S6**).

To evaluate the importance of these cells in microenvironmental interactions, we performed cellular communication analysis using the CellChat software and ranked the cell populations based on the number of communications. The analysis showed that the top ten cell populations were iCAF, myCAF, pericyte, endothelial cells, VSMC, myoepithelial cell, EMT-like CAF, macrophage, cDC2, and cDC3 (**Figure 3B**). In the analysis of cellular interactions between TNBC and normal tissues, we further compared differences in interaction frequency (**Figure 3C**) and interaction strength (**Figure 3D**). Interaction frequency and strength respectively reflect the number of communication events occurring between different cell types and the intensity of signal transmission during cell communication. The results showed that in TNBC tissues, iCAF, EMT-like CAF, myCAF, endothelial cells, myoepithelial cells, pericytes and VSMC exhibited increased interaction frequency with other cells in the TME (**Figure 3C**), indicating that these cells engaged in more extensive communication within the TNBC microenvironment, contributing to an overall more active signal transmission. In contrast, apCAF and NAF related interaction frequency showed a significant decrease. Further analysis of interaction strength revealed that iCAF, myCAF, pericytes, EMT-like CAF, and VSMC also exhibited enhanced interaction strength with other cells. This suggests that these cells not only participate in more frequent communication events but may also influence TNBC microenvironment dynamics through more potent signal transmission. These findings indicate that these cells may play a crucial regulatory role in TNBC, influencing tumor initiation and progression. Given the advantages of bulk RNA-seq data in terms of larger sample sizes and richer clinical information, we performed prognostic analysis to further evaluate the clinical significance of these cell infiltrations in TNBC samples based on the results of cell type analysis. Using ssGSEA, we calculated the signature scores for the previous cell types in TNBC patients (**Supplementary Table S8**). The results showed that a high abundance of myCAF, VSMC, and pericyte in TNBC tumors was associated with poor survival prognosis, whereas a high abundance of CD8_STMN1, cDC2, CD4_CXCL13, and NK cells was associated with better survival prognosis (**Figures 3E-H**, **Supplementary Figure S3A-C**). Integrating the findings from tissue distribution characteristics, cell communication, and prognostic analysis suggests that myCAF, VSMC, and pericyte may have an important impact on the initiation and progression of TNBC.

### 3.3 Identification of Key Modules and MVP-Related Genes in Bulk RNA-seq

**Figure 4:**
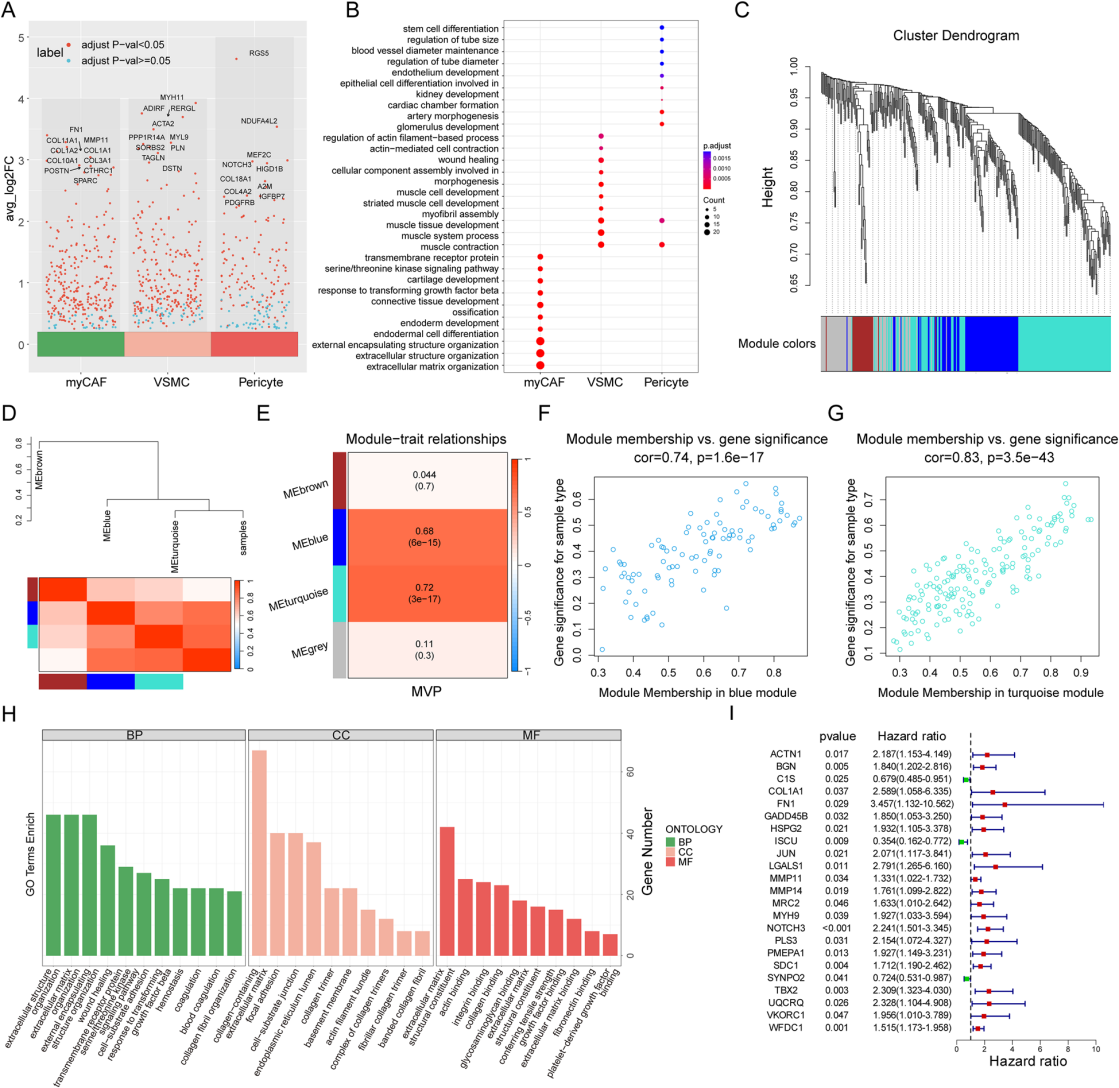
Identification of the myCAF-VSMC-Pericyte related genes. **(A)** Volcano plot highlights marker genes of three stromal cell populations (myCAF, VSMC, and Pericyte), with Top 10 genes labeled. The X-axis represents three types of stromal cells, while the Y-axis represents the average log2 fold change value, where higher values indicate a greater upregulation of genes. Each dot represents a gene, and the color of the dot indicates the significance threshold: red denotes significantly differentially expressed genes, while blue represents genes with no significant differential expression. **(B)** Gene Ontology Enrichment Analysis of myCAF, VSMC and Pericyte with their marker genes. The X-axis represents three types of stromal cells, while the Y-axis lists significantly enriched biological processes (BP). The dot size (Count) indicates the number of genes enriched in each pathway, with larger dots representing a greater number of enriched genes in the processes. The dot color (p.adjust) represents statistical significance, where a deeper color indicates a higher enrichment significance of the GO term. **(C)** WGCNA gene hierarchical clustering dendrogram. The X-axis represents all input genes, while the Y-axis indicates the hierarchical clustering height, reflecting the similarity in gene expression patterns. Different colored modules represent distinct co-expression gene modules, where genes with similar expression patterns are grouped into the same module. **(D)** Module-trait correlation heatmap and dendrogram. This figure displays the correlation between modules and key traits (MVP). The hierarchical clustering dendrogram at the top illustrates the clustering relationships among co-expression gene modules and their association with trait information (MVP). Similar modules are clustered into the same branch. The branch length represents the correlation between modules, with shorter branches indicating more similar expression patterns among modules. The color of the heatmap at the bottom represents the correlation strength, where red indicates a strong positive correlation and blue indicates a strong negative correlation. Each row corresponds to a co-expression gene module, while the rightmost column represents the trait information (MVP) of the samples. **(E)** Module-trait correlation heatmap. The X-axis represents trait information (MVP), while the Y-axis lists the co-expression gene modules identified by WGCNA (MEbrown, MEblue, MEturquoise, and MEgrey). The heatmap color indicates the strength of the correlation between modules and the trait. The numerical values display the correlation coefficient and p-value, with the value in parentheses representing the p-value, indicating statistical significance. **(F, G)** The scatter plots illustrate the correlation between module membership (MM) and gene significance (GS) in the blue **(F)** and turquoise **(G)** modules. The X-axis represents module membership (MM). It indicates how central a gene is within the module. This is measured by its correlation with the module characteristic gene. The Y-axis represents gene significance (GS), reflecting the correlation between the gene and the phenotype (MVP). A higher GS value indicates a stronger correlation between gene expression levels and the studied phenotype variable (MVP). Cor represents the correlation between MM and GS, where a higher cor value suggests that key genes in the module not only occupy a central position within the module (high MM value) but are also highly associated with the clinical phenotype under study (high GS value). The p-value represents statistical significance. **(H)** GO enrichment analysis of MVPRGs in Biological Process (BP), Cellular Component (CC), and Molecular Function (MF) categories. The X-axis lists significantly enriched GO terms in the GO enrichment analysis. The Y-axis represents the number of genes enriched in each GO term. Colors differentiate the three GO categories: BP, CC, and MF. **(I)** Forest plot of univariate cox regression analysis results. This plot presents the survival relevance of MVPRGs. The left side lists gene names. The p-value measures the statistical significance of the gene’s association with survival risk, indicating whether its expression significantly impacts patient survival. The hazard ratio (HR) quantifies the relationship between gene expression and survival risk. HR > 1 suggests a risk factor and higher expression is associated with poorer survival outcomes. HR < 1 indicates a protective factor and higher expression correlates with better survival outcomes.

We performed differential gene expression (DEG) analysis for these three cell groups (**Figure 4A**) and Gene Ontology (GO) enrichment analysis (**Figure 4B**; **Supplementary Table S9**). In the myCAF cell population, we detected biological processes associated with extracellular matrix organization and connective tissue development. On the other hand, VSMC exhibited distinct GO features, showing enrichment in muscle contraction and development processes. Pericytes were significantly enriched in processes related to vascular diameter maintenance and regulation, endothelial development, and arterial morphogenesis, which were associated with vascular formation and stabilization. Additionally, pericytes also showed contractility, being enriched in features of muscle contraction and development. These results further confirmed their cellular identities and their functional specificity in tumor stroma remodelling, muscle contraction, and vascular formation and stabilization (38–40).

The ssGSEA algorithm is commonly used to evaluate changes in biological processes and pathway activity in individual samples. In this study, we used the ssGSEA algorithm to obtain MVP scores for each TNBC sample, which were subsequently used as phenotypic data for WGCNA analysis. To identify the modules significantly associated with MVP scores, we applied WGCNA analysis to the GSE58812 dataset. After excluding outlier samples, a co-expression network was constructed based on 768 MVP-related DEGs, with a soft threshold power of 3 chosen to ensure a scale-free network (**Figure 4C**, **Supplementary Figure S3D-E**). Ultimately, four modules were obtained (**Figure 4D**). Our findings indicated that the MEblue module and the MEturquoise module were closely related to MVP scores in bulk RNA-seq with correlation coefficients of 0.68 and 0.72, respectively (**Figure 4E**).

Moreover, scatter plots of gene significance (GS) versus module membership (MM) for the blue and turquoise modules showed significant correlations (**Figures 4F, G**), suggesting that the genes within these modules may have important MVP-related functions. A total of 259 genes were ultimately identified in the two modules, referred to as MVPRGs, which were considered the marker genes most relevant to myCAF, pericyte, and VSMC populations at both the bulk and single-cell transcriptomic levels (**Supplementary Figure S3F**, **Supplementary Table S10**).

The GO enrichment results for MVPRGs illustrated that they were significantly enriched in multiple biological processes (BPs), including extracellular matrix organization, extracellular structure organization, wound healing, and blood coagulation, indicating that MVPRGs play an important role in tissue repair, extracellular matrix construction, vascular homeostasis, and mediating cell-matrix interactions (**Figure 4H**). Regarding cellular components (CCs), MVPRGs were significantly enriched in collagen-containing extracellular matrix, focal adhesion, and actin filament bundles, highlighting their key role in extracellular matrix construction and cell adhesion. For molecular functions (MFs), MVP genes were significantly enriched in extracellular matrix structural components, actin binding, and integrin binding, implying that these genes are essential for maintaining cytoskeletal integrity and mediating cell-matrix interactions (**Supplementary Table S11**).

Subsequently, we performed univariate Cox regression analysis on the 259 MVP genes, identifying 23 genes with significant prognostic value, which were then used for further model construction (**Figure 4I**).

### 3.4 Construction of prognostic signature based on ensemble machine learning

**Figure 5:**
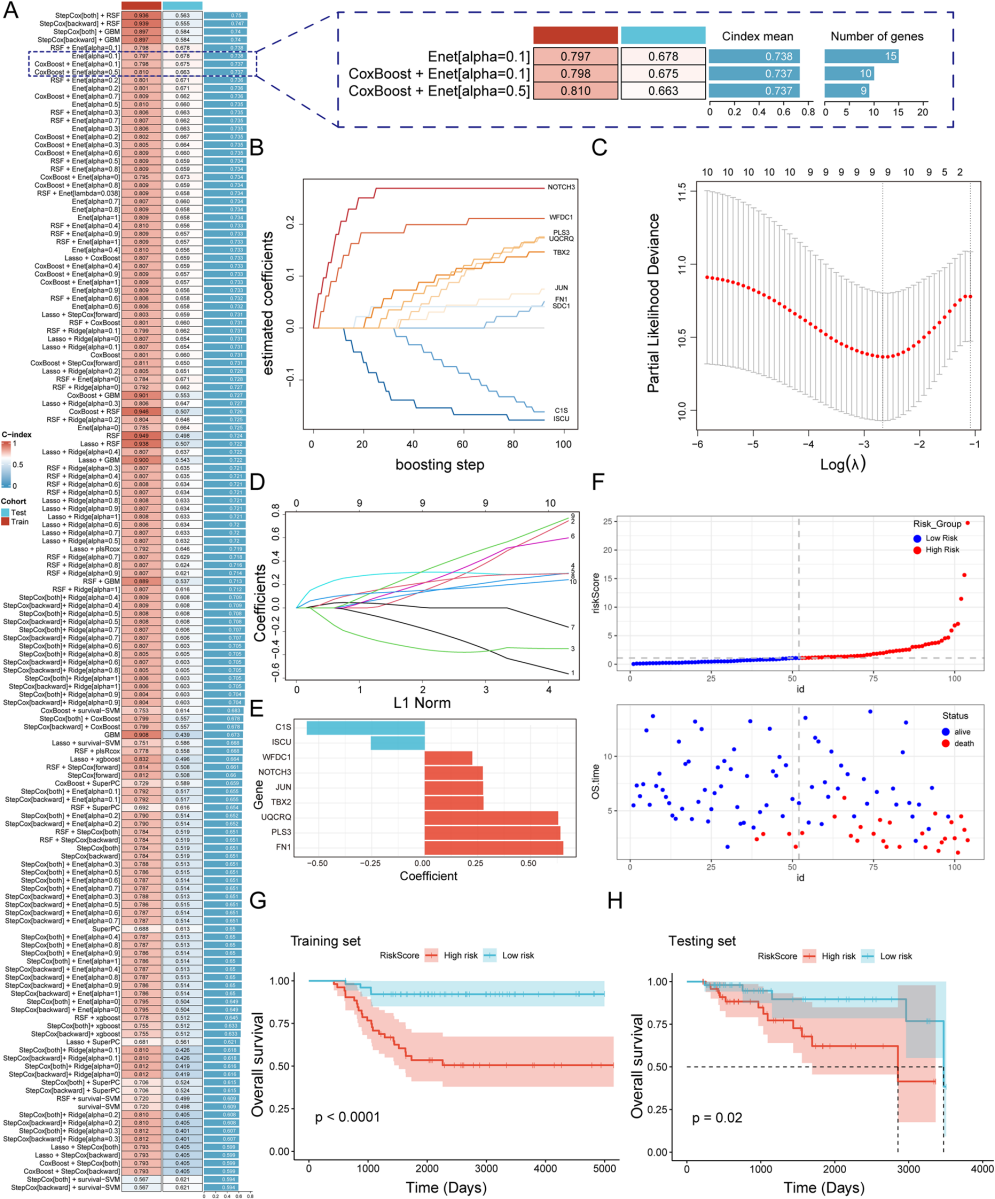
A consensus MVPRS was constructed and validated through an integrative procedure based on machine learning **(A)** A total of 101 prediction models were developed using a tenfold cross-validation framework, and the C-index for each model was subsequently calculated across all validation datasets. The Y-axis represents model names, with each row corresponding to a model combination. The color bar labeled ‘Cohort’ distinguishes the data source used in the model (Test and Train). The C-index value, ranging from 0 to 1, indicates the discriminative ability of the model for survival prediction, with higher values representing better predictive performance. On the right, a further comparison is provided between selected models in terms of their C-index mean and the number of feature genes. **(B)** Coefficient path plot for the CoxBoost model. This plot illustrates the changes in regression coefficients of different genes during the CoxBoost training process as the number of boosting iterations increases. The X-axis represents the number of iterations (0–100), indicating the model optimization process. The Y-axis represents the regression coefficients, reflecting the magnitude and direction of each gene’s impact on survival risk. **(C-D)** Visualization analysis of elastic net regression in the GSE58812 cohort, including optimal λ selection **(C)** and regression coefficient paths **(D)**. In **(C)**, the X-axis represents the logarithm of the regularization parameter λ, while the Y-axis shows the partial likelihood deviance. The lowest point of the curve corresponds to the optimal λ value, indicating the model’s best generalization ability at this λ value. In **(D)**, the X-axis represents the normalized path of the L1 penalty function, and the Y-axis represents the regression coefficients of genes. The curves illustrate the selection process of different genes as they are gradually filtered during regularization. **(E)** Bar plot showing key genes selected by stepwise cox regression and their regression coefficients. The X-axis represents the regression coefficients, while the Y-axis lists the nine key genes selected in the Cox regression model. Colors differentiate gene types: red indicates genes associated with increased survival risk, while blue represents genes linked to reduced survival risk. **(F)** Risk score and survival status distribution of patients in the GSE58812 cohort. The top plot shows the distribution of patient risk scores, where the X-axis represents patient IDs, and the Y-axis indicates individual risk scores. Colors distinguish between the high-risk group (red) and the low-risk group (blue). The bottom plot displays the distribution of overall survival time and survival status. The X-axis remains consistent with the top plot, representing patient sample IDs, while the Y-axis indicates overall survival time in years. Colors differentiate survival status: red represents deceased patients, while blue represents surviving patients. **(G, H)** The Kaplan-Meier survival curves illustrate survival differences between MVPRS high- and low-risk groups in the training **(G)** and testing **(H)** cohorts. The X-axis represents follow-up time (days), while the Y-axis indicates the proportion of surviving patients at different time points. Strata grouping is shown with red and blue curves representing the high-risk and low-risk groups, respectively. The shaded area denotes the confidence interval (CI) for survival probability in each group.

To construct the consensus MVP-related signature (MVPRS), we used a combination of 101 machine-learning algorithms to analyze the 23 prognostic genes identified from the univariate Cox regression analysis. The GSE58812 dataset was used as the training set, while the TCGA-TNBC dataset served as the validation set, with detailed patient clinical characteristics summarized in **Supplementary Table S1**. Using a tenfold cross-validation framework, we fitted 101 predictive models to the training set and calculated the concordance index (C-index) for both training and validation sets (**Figure 5A**).

Among the 101 models, we selected the top five predictive models based on their average C-index, ultimately constructed using either the Random Survival Forest (RSF) algorithm or the Gradient Boosting Machine (GBM) algorithm. Although these five models performed well in the training set, the top four models exhibited poor performance in the validation set, with a C-index below 0.6. These models showed a marked performance discrepancy between the training and validation sets, indicating a potential risk of overfitting, and were therefore excluded. Additionally, the fifth model demonstrated limited discriminatory ability in the validation set, as the survival differences between high- and low-risk groups did not reach statistical significance. Therefore, we excluded these models that were overly fitted to the training set from further consideration. Next, we focused on the Enet[alpha=0.1], CoxBoost+Enet[alpha=0.1], and CoxBoost+Enet[alpha=0.5] models, as these three models demonstrated good predictive performance in both the training and validation sets. However, the Enet[alpha=0.1] model included a total of 15 genes, while the CoxBoost+Enet[alpha=0.1] and CoxBoost+Enet[alpha=0.5] models included 10 and 9 genes, respectively, while still achieving comparable predictive efficacy (**Figure 5A**). Given that fewer genes are preferable when the C-index differences are marginal, we determined that the model constructed using the CoxBoost+Enet[alpha=0.5] method was a highly accurate predictive model closely associated with MVP genes (**Figure 5A**). Additionally, we added time-dependent ROC (timeROC) analysis for different models to provide a more comprehensive evaluation. The results showed that the CoxBoost + Enet [α = 0.5] model demonstrated good ROC curve (AUC) performance at years 3, 5, and 7 in both the training and validation sets (**Supplementary Figure S4**), further confirming its robust predictive capability.

In this study, we applied CoxBoost combined with Elastic Net (α = 0.5) for feature selection and ultimately constructed a multivariable Cox proportional hazards model, identifying a final set of nine genes (**Figures 5B-E**, **Supplementary Table S12**). Based on this model, we calculated risk scores for patients and stratified them into high-risk (MVPRS > median) and low-risk (MVPRS ≤ median) groups according to the median value. Notably, as risk scores increased, the number of deceased patients also gradually increased (**Figure 5F**). Additionally, in both the training and validation sets, patients in the high-risk group had a significantly shorter overall survival (OS) compared to those in the low-risk group (**Figures 5G, H**).

Furthermore, an in-depth check of the origins of these nine genes revealed that C1S is primarily derived from myCAF, WFDC1 mainly from VSMC and ISCU. PLS3 is from both myCAF and VSMC. NOTCH3 and TBX2 are predominantly expressed in Pericytes and VSMC, while JUN, UQCRQ and FN1 are found across myCAF, VSMC, and Pericyte, covering all three stromal cell subpopulations (**Supplementary Figure S5**). Although not all three subpopulations contribute genes equally, our feature selection strategy for constructing the MVPRS model was based on the overall marker gene set of myCAF, VSMC, and Pericytes, rather than a common gene set shared by all three. Given that this model remains effective in predicting TNBC prognosis and reflects the functional roles of key stromal cells within the tumor microenvironment (TME), we retained the MVPRS designation to highlight its broad association with stromal cell populations.

### 3.5 Evaluation of the MVPRS model with the development and validation of a clinical nomogram

**Figure 6:**
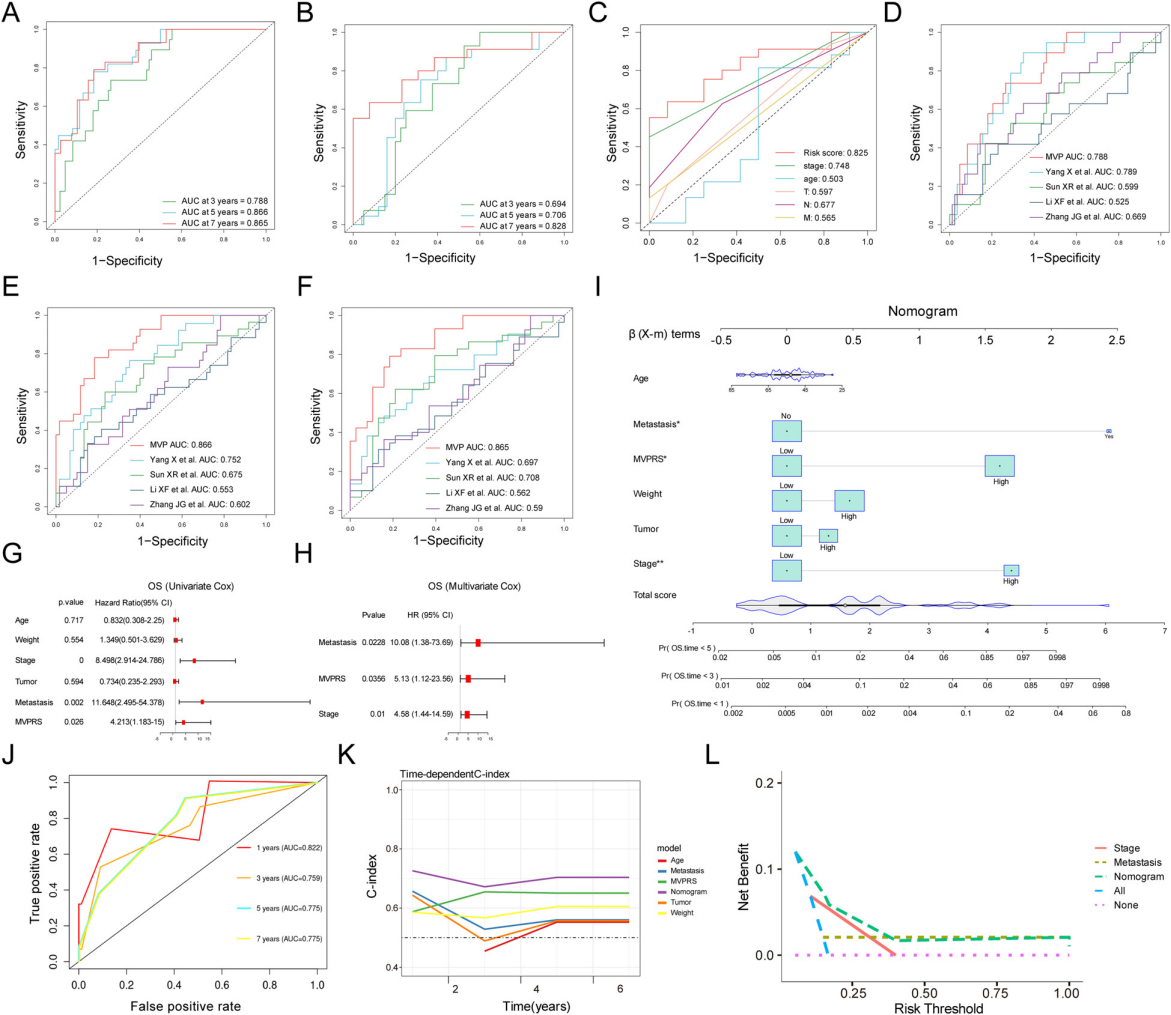
Evaluation of the MVPRS model with Nomogram establishment and validation. **(A, B)** ROC curves illustrate the specificity and sensitivity of MVPRS in predicting 3, 5, and 7-year OS in the training set **(A)** and validation set **(B)**. The X-axis represents the false positive rate, while the Y-axis represents sensitivity, indicating the true positive rate. The ROC curves in different colors represent the predictive performance of MVPRS at 3, 5, and 7 years. **(C)** The ROC curve compares the prognostic performance of MVPRS with other clinical features (age, stage, T, N, M). The X-axis represents the false positive rate, while the Y-axis represents the true positive rate. Different colors distinguish the ROC curves of various predictive factors. **(D-F)** The prognostic performance of MVPRS is compared with four published survival prediction models for TNBC in predicting overall survival at 3 years **(D)**, 5 years **(E)**, and 7 years **(F)**. The X-axis represents the false positive rate, while the Y-axis represents the true positive rate. Different colors distinguish the ROC curves of various models. **(G, H)** Univariate and multivariate analyses of clinical characteristics and MVPRS for OS in the validation cohort. **(I)** Development of a nomogram incorporating MVPRS alongside clinical features, including Age, Metastasis, Weight, Tumor and Stage. The top section (β (X-m) terms) displays the distribution of regression coefficients (β values) for each variable, indicating their contribution to survival prediction. Total Score represents the overall score calculation, which is used to determine each patient’s score on the nomogram and ultimately predict the probability of death at 1, 3, and 5 years. **(J)** ROC curves illustrate the nomogram’s ability to predict outcomes at 3, 5, and 7 years. The X-axis represents the false positive rate, while the Y-axis represents the true positive rate. Different colors distinguish the ROC curves of the nomogram at 1, 3, 5, and 7 years. **(K)** The C-index is compared across the nomogram and other clinical characteristics, including Age, Metastasis, Weight, Tumor and MVPRS. The X-axis represents follow-up time in years, while the Y-axis represents the C-index, which reflects the survival prediction ability of the model. Different colors distinguish the C-index curves of various features. **(L)** DCA for net benefit of different models and clinical characteristics (Stage, Metastasis, MVPRS, and the nomogram). The X-axis represents the individualized decision threshold, which indicates the level of survival risk at which a doctor or patient would accept intervention. The Y-axis represents the net benefit of the model at a specific risk threshold, where a higher net benefit indicates greater clinical value of the model at that threshold.

The ROC curve analysis indicated that, in the training set, the AUC for MVPRS reached 0.788, 0.866, and 0.865 at 3-year, 5-year, and 7-year intervals, respectively. In the validation set, the AUC values were 0.694, 0.706, and 0.828, respectively (**Figures 6A, B**). These results demonstrated the strong discriminative power of MVPRS. Moreover, we compared the AUC of MVPRS with other clinical characteristics, including age, tumor, node, metastasis and stage. The results demonstrated that the AUC of MVPRS outperformed those of the other clinical characteristics (**Figure 6C**).

Additionally, we evaluated the prognostic value of MVPRS compared to four other risk scores, including those proposed by Yang X et al., Sun XR et al., Li XF et al., Zhang JG et al., in predicting TNBC patient outcomes (41–44). Our findings indicated that the AUC of MVPRS for predicting 3-year, 5-year, and 7-year survival was significantly higher than that of the other four risk models (**Figure 6D-F**). These results highlighted the outstanding predictive accuracy of MVPRS in forecasting the prognosis of TNBC patients.

To evaluate whether MVPRS serves as an independent prognostic factor for TNBC, we performed univariate and multivariate Cox regression analyses on OS in the validation dataset (**Figures 6G, H**). The results showed that MVPRS was a significant risk factor for OS in both univariate (HR > 1, p < 0.001) and multivariate (HR 1.802, p < 0.001) analysis, remaining an independent prognostic factor, which indicated its robust prognostic capability in TNBC patients.

To enhance the clinical utility of MVPRS, we developed a nomogram that integrates MVPRS with additional clinical characteristics (**Figure 6I**). The AUC for the nomogram at 3, 5, and 7 years was 0.759, 0.775, and 0.775, respectively, demonstrating strong predictive performance (**Figure 6J**). In addition, the concordance index (C-index) validated the nomogram’s reliability and stability in forecasting OS, outperforming the performance of other individual clinical features (**Figure 6K**). DCA demonstrated that the nomogram achieved higher net clinical benefit compared to other clinical characteristics (**Figure 6L**). These findings suggested that the MVPRS-based nomogram was a reliable and precise tool for individualized prognosis prediction in TNBC patients.

### 3.6 Correlation between immune microenvironment, immunotherapy response, and MVPRS

**Figure 7:**
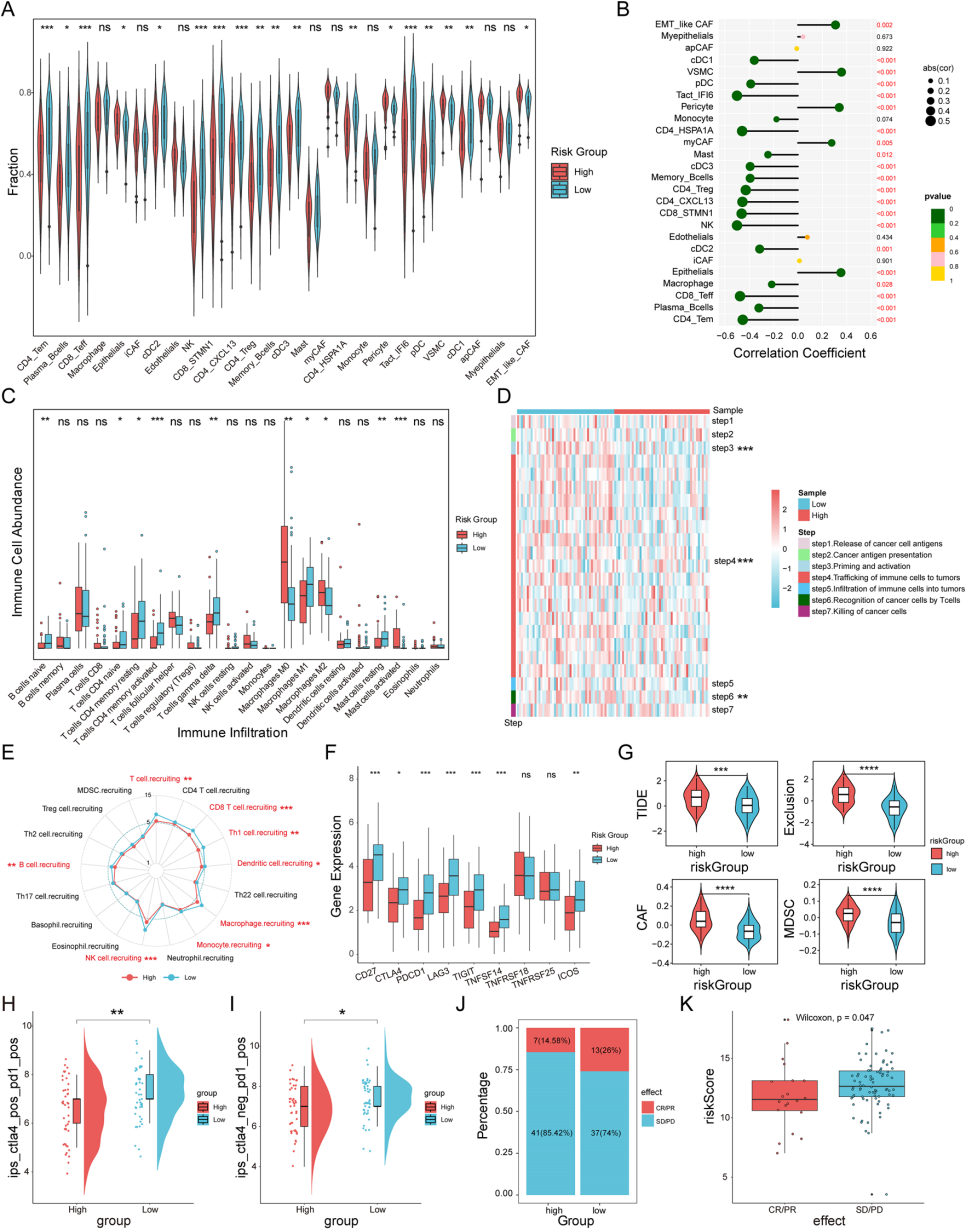
The immune landscape and immunotherapy response associated with MVPRS in TNBC. **(A)** The abundance of each TME-infiltrated cell type between high- and low-risk groups, quantified by the ssGSEA algorithm. **(B)** Lollipop plot showing the correlation between TME-infiltrating cells and MVPRS. **(C)** The abundance of 22 immune-infiltrated cell types between high- and low-risk groups, quantified by the CIBESORT algorithm. **(D)** Heatmap illustrating the differences in the activity of the seven-step anti-cancer immunity cycle between high- and low-risk groups. **(E)** Radar plot depicting the difference in immune cell recruitment capabilities between MVPRS subgroups. **(F)** Box plots showing expression levels of immune checkpoint genes in high and low MVP risk groups. **(G)** Violin plots depicting TIDE scores, exclusion scores, CAF scores, and MDSC scores between high and low MVP risk groups. **(H)** The IPS score of ips_ctla4_pos_pd1_pos compared across high- and low-risk groups. **(I)** The IPS score of ips_ctla4_neg _pd1_pos compared across high- and low-risk groups. **(J)** The distribution of CR/PR and SD/PD among patients undergoing immunotherapy in high- and low-risk groups within the GSE91061 cohort. **(K)** The Boxplot depicting the MVPRS score between patients with CR/PR and those with SD/PD in the GSE91061 cohort. The symbols "*", "**", "***" represent p < 0.05, p < 0.01, and p < 0.001, respectively; "ns" stands for not significant (p ≥ 0.05).

To evaluate the immune infiltration status of TNBC samples, we used the ssGSEA algorithm to quantitatively analyze the abundance of microenvironmental cells in each TNBC sample (**Figure 7A**). The results indicated that the high-risk group had higher abundances of epithelial cells, pericyte, VSMC, and EMT-like CAF cells. In contrast, cell types with antitumor activity, such as CD4_Tem, Plasma_Bcells, CD8_Teff, cDC2, NK cells, and Memory_Bcells, were more enriched in the low-risk group. Similarly, Spearman correlation analysis identified 22 cell types that were significantly associated with MVPRS (*P* < 0.05), most of which were immune cells showing a negative correlation with MVPRS (**Figure 7B**). Additionally, using the CIBERSORT algorithm to quantify the abundance of infiltrating immune cells in each sample, the results similarly showed that cell types related to antitumor immunity, such as B cells naïve, T cells CD4 naïve, T cells CD4 memory resting, T cells CD4 memory activated, T cells gamma delta, Macrophages M1 and mast cells resting, were mainly enriched in the low-risk group. On the other hand, Macrophages M2, which are known to promote tumor progression, were enriched in the high-risk group (**Figure 7C**, **Supplementary Table S13**).

Given the complexity of intratumoral immune processes and the TME, immune activation and exhaustion states cannot be fully captured by merely assessing the abundance of infiltrating immune cells. A more comprehensive understanding of the antitumor functions of immune cells, along with enhanced guidance for immunotherapy, can be achieved by analyzing the activity at each stage of the cancer immunity cycle (36). As shown in **Figure 7D** and **Supplementary Figure S6B**, there were significant differences in the activities of steps in the cancer immunity cycle between MVPRS risk subgroups. Specifically, step 3 (priming and activation), step 4 (immune cell trafficking to the tumor), and step 6 (T-cell recognition of cancer cells) showed weaker activity in the high-risk group. Moreover, we further analyzed the differences in immune cell recruitment capabilities in “step 4 - immune cell trafficking to the tumor” between MVPRS risk subgroups (**Figure 7E** and **Supplementary Figure S6C**). The results showed that the high-risk group exhibited significantly reduced capabilities in recruiting immune cells, including T cells, CD8 T cells, Th1 cells, Dendritic cells, Macrophages, Monocytes, NK cells, and B cells. These findings suggested that the high-risk group demonstrated poorer antitumor activity in the functional immune cell cycle.

Previous research has shown that elevated expression of immune checkpoints correlates with improved responses to ICI therapy (45, 46). Therefore, we analyzed the expression levels of immune checkpoints across MVPRS risk subgroups. As depicted in **Figure 7F**, the low-risk group exhibited significantly higher expression of most immune checkpoints, including CD27, PD1, CTLA-4, TIGIT, LAG3, TNFRSF14, and ICOS.

Next, we calculated the TIDE scores for the MVPRS risk subgroups. The results showed that the high-risk group had higher TIDE scores, exclusion scores, CAF scores, and MDSC scores (**Figure 7G**, **Supplementary Figure S6D**, **Supplementary Table S14**), indicating a higher potential for immune evasion and potentially poorer ICI performance in the high-risk group.

To further confirm these findings, we examined the IPS scores derived from the TCIA database. Elevated IPS scores are linked to improved responses to ICI therapy, which includes four types: CTLA4+/PD1+ therapy (ips_ctla4_pos_pd1_pos), CTLA4+/PD1- therapy (ips_ctla4_pos_pd1_neg), CTLA4-/PD1+ therapy (ips_ctla4_neg_pd1_pos), and CTLA4-/PD1- therapy (ips_ctla4_neg_pd1_neg). The results showed that for CTLA4+/PD1+ and CTLA4-/PD1+ therapies, the high-risk group had significantly lower IPS scores, suggesting that patients in the high-risk group had a lower response to both PD-1 monotherapy and the combination of PD-1 and CTLA4 therapies compared to patients in the low-risk group (**Figure 7H, I**, **Supplementary Figure S6A**).

To further assess the predictive capability of MVPRS for patient response to immunotherapy, we included the GSE91061 cohort, which includes patients treated with nivolumab (anti-PD1 therapy) either alone or in combination with ipilimumab (anti-CTLA4 therapy). Based on the MVPRS signature, we calculated risk scores for this cohort and stratified patients into high-risk and low-risk groups. The results showed that the proportion of patients with stable disease/progressive disease (SD/PD) was higher in the high-risk group, whereas the low-risk group had more cases with complete response/partial response (CR/PR) (**Figure 7J**). Moreover, the risk scores of CR/PR patients were significantly lower compared to those of SD/PD patients (**Figure 7K**). Taken together, these findings supported the ability of MVPRS to predict immunotherapy efficacy, indicating that patients in the high-risk group are less likely to benefit from these treatments.

### 3.7 Potential molecular mechanism of MVPRS

**Figure 8:**
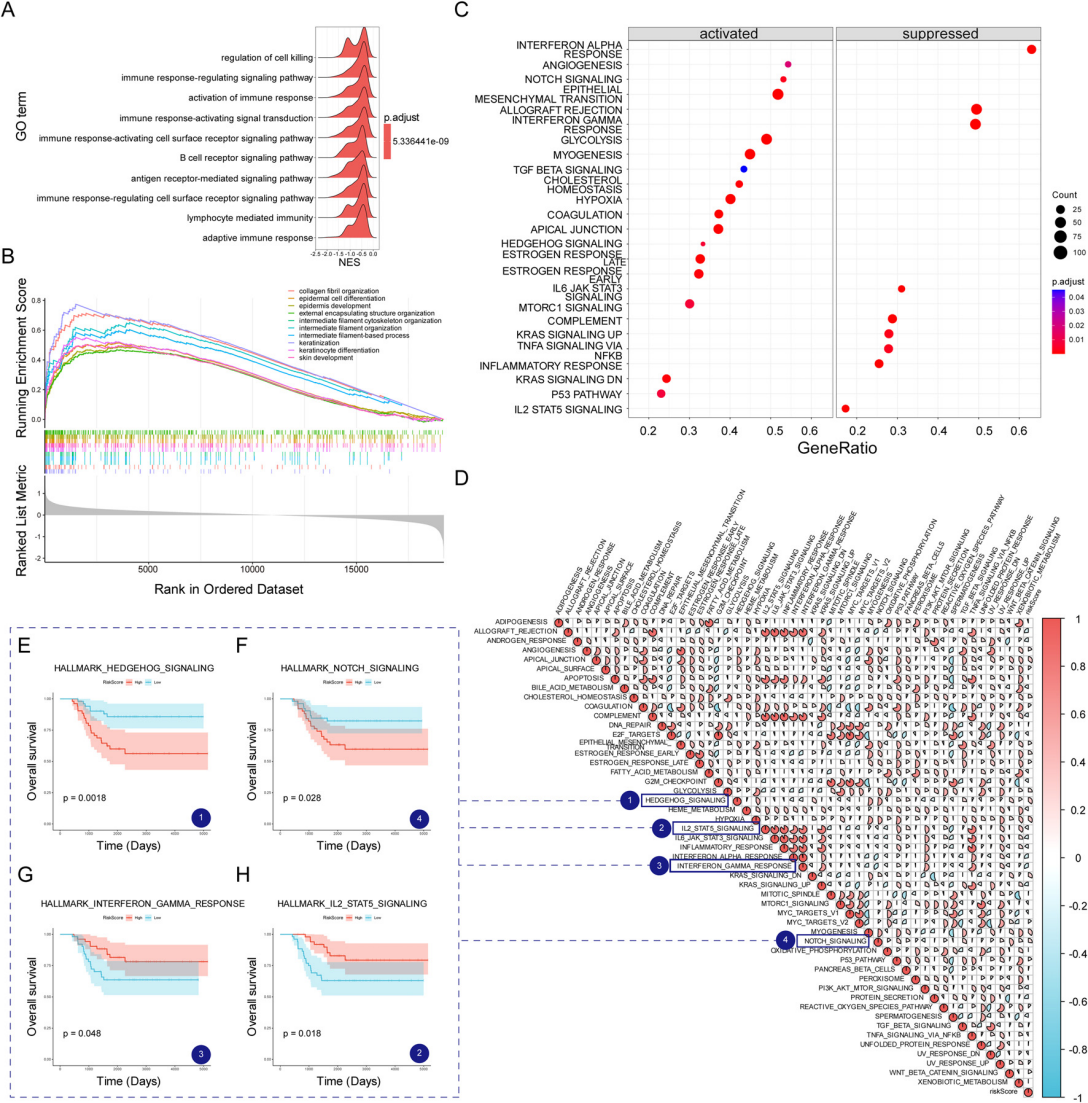
The transcriptomic characteristics of TNBC patients across different MVPRS subgroups. **(A)** Ridge plot illustrating the GO terms significantly enriched in the low-risk group. The X-axis represents the normalized enrichment score, indicating the degree of enrichment of a specific GO term in the low-risk group. The Y-axis lists the top 10 significantly enriched GO terms in the low-risk group. **(B)** GO terms significantly enriched in the high-risk group, as identified by GSEA analysis. The X-axis represents gene ranking, where all genes are ordered based on expression changes from high to low, forming a continuous ranked gene list. The Y-axis indicates the enrichment score (ES) of the gene set, with higher peak values signifying a greater degree of enrichment for the GO term in the high-risk group. Colors distinguish different GO biological process terms. Vertical lines in the middle mark the positions of genes from the GO term within the ranked gene list. The gray bar plot at the bottom represents gene ranking scores. **(C)** Differences in hallmark pathway activities between the high- and low-risk groups as evaluated by GSVA. The X-axis represents GeneRatio, indicating the enrichment ratio of genes within a gene set. A higher value signifies a greater degree of enrichment for the pathway in the dataset. The Y-axis lists Hallmark signaling pathways enriched in the high- and low-risk groups. The dot size (Count) represents the number of genes enriched in each pathway, while the dot color (p.adjust) reflects the significance level, with deeper colors indicating stronger pathway enrichment significance. **(D)** Association between the risk scores and hallmark pathway activities assessed through GSVA. The X-axis and Y-axis list a series of Hallmark signaling pathways and MVPRS risk scores. Each matrix pane represents the strength of the correlation between two pathways. The circle size and color indicate the correlation strength and direction: larger red circles represent a strong positive correlation, while larger blue circles indicate a strong negative correlation. **(E-H)** Kaplan–Meier survival plots depict the notable association between OS and GSVA scores for HEDGEHOG_SIGNALING **(E)**, NOTCH_SIGNALING **(F)**, INTERFERON_GAMMA RESPONSE **(G)**, and IL2_STAT5 SIGNALING **(H)**. The X-axis represents follow-up time (days), while the Y-axis indicates the proportion of surviving patients at different time points. The curve colors represent the high- and low-risk groups based on Hallmark pathway enrichment scores, with the red curve indicating the high-risk group and the blue curve indicating the low-risk group. The shaded area represents the confidence interval (CI) for survival probability in each group.

To explore the molecular mechanisms linking MVPRS to TNBC prognosis, we conducted a functional enrichment analysis. Using GSEA with GO gene sets, we observed that the low-risk group was enriched in immune-related biological processes, including regulation of cell killing, immune response-regulating signalling pathway, and activation of immune response, among others (**Figure 8A, B**, **Supplementary Table S15**). In contrast, the high-risk group was significantly enriched in pathways related to collagen fibril organization, epithelial cell differentiation, epidermal development, intermediate filament cytoskeleton organization, and keratinization, suggesting that their biological characteristics mainly involve extracellular matrix remodelling and abnormal differentiation of epithelial tissues. The enrichment of collagen fibrils may imply enhanced stromal remodelling within the tumor microenvironment, promoting tumor invasion and metastasis (47). Enrichment in epithelial cell differentiation and keratinization could be linked to an imbalance in cell proliferation and differentiation, typically associated with malignant tumor progression (48). Therefore, these enriched pathways might indicate that patients in the high-risk group possess more aggressive characteristics and a poorer prognosis.

Furthermore, the GSVA analysis of Hallmark gene sets revealed that the high-risk group had higher activity in tumor-related pathways (**Figure 8C**, **Supplementary Table S16**), such as ANGIOGENESIS, NOTCH SIGNALING, EPITHELIAL MESENCHYMAL TRANSITION, GLYCOLYSIS, MYOGENESIS, HYPOXIA, COAGULATION, MTORC1 SIGNALING, and HEDGEHOG SIGNALING.

Conversely, the low-risk group showed stronger activity in pathways related to INTERFERON RESPONSE, ALLOGRAFT REJECTION, IL6 JAK STAT3 SIGNALING, COMPLEMENT, IL2_STAT5_SIGNALING, and INFLAMMATORY RESPONSE. Analysis of the correlation between MVPRS and Hallmark pathway scores provided additional evidence for these findings (**Figure 8D**), highlighting the strong association of MVPRS with cancer-related biological processes.

Furthermore, to further validate the relationship between GSVA results and the three stromal cell subpopulations, we added a correlation analysis between Hallmark pathway scores and the gene set scores of myCAF, VSMC, and Pericytes (**Supplementary Figure S7**). The results showed that myCAF, VSMC, and Pericytes gene set scores were significantly positively correlated with pathways such as ADIPOGENESIS, ANDROGEN_RESPONSE, ANGIOGENESIS, APICAL_JUNCTION, APICAL_SURFACE, APOPTOSIS, COAGULATION, EPITHELIAL_MESENCHYMAL_TRANSITION, ESTROGEN_RESPONSE_EARLY, HEDGEHOG_SIGNALING, HYPOXIA, KRAS_SIGNALING_UP, MYOGENESIS, NOTCH_SIGNALING, P53_PATHWAY, PANCREAS_BETA_CELLS, TGF_BETA_SIGNALING, UV_RESPONSE_DN and XENOBIOTIC_METABOLISM. In contrast, they were significantly negatively correlated with DNA_REPAIR, E2F_TARGETS, G2M_CHECKPOINT, MTORC1_SIGNALING, MYC_TARGETS_V1, MYC_TARGETS_V2, OXIDATIVE_PHOSPHORYLATION, REACTIVE_OXYGEN_SPECIES_PATHWAY, SPERMATOGENESIS and UNFOLDED_PROTEIN_RESPONSE. These findings further suggest that these stromal cell subpopulations may play a critical role in remodeling the TNBC microenvironment and tumor progression through distinct Hallmark signaling pathways.

To assess the relationship between Hallmark pathways and TNBC prognosis, Kaplan-Meier curve analysis was conducted. The findings revealed that pathways positively linked to MVPRS, such as HEDGEHOG_SIGNALING and, NOTCH_SIGNALING were associated with poor prognosis (**Figure 8E, F**). Conversely, pathways inversely associated with MVPRS, such as INTERFERON_GAMMA_RESPONSE and IL2_STAT5_SIGNALING, were linked to a favorable prognosis (**Figures 8G, H**). These results indicated that the activation or suppression of these pathways may influence the varying prognostic outcomes within MVPRS risk subgroups.

### 3.8 FN1 promotes the malignant phenotype of TNBC cells

**Figure 9:**
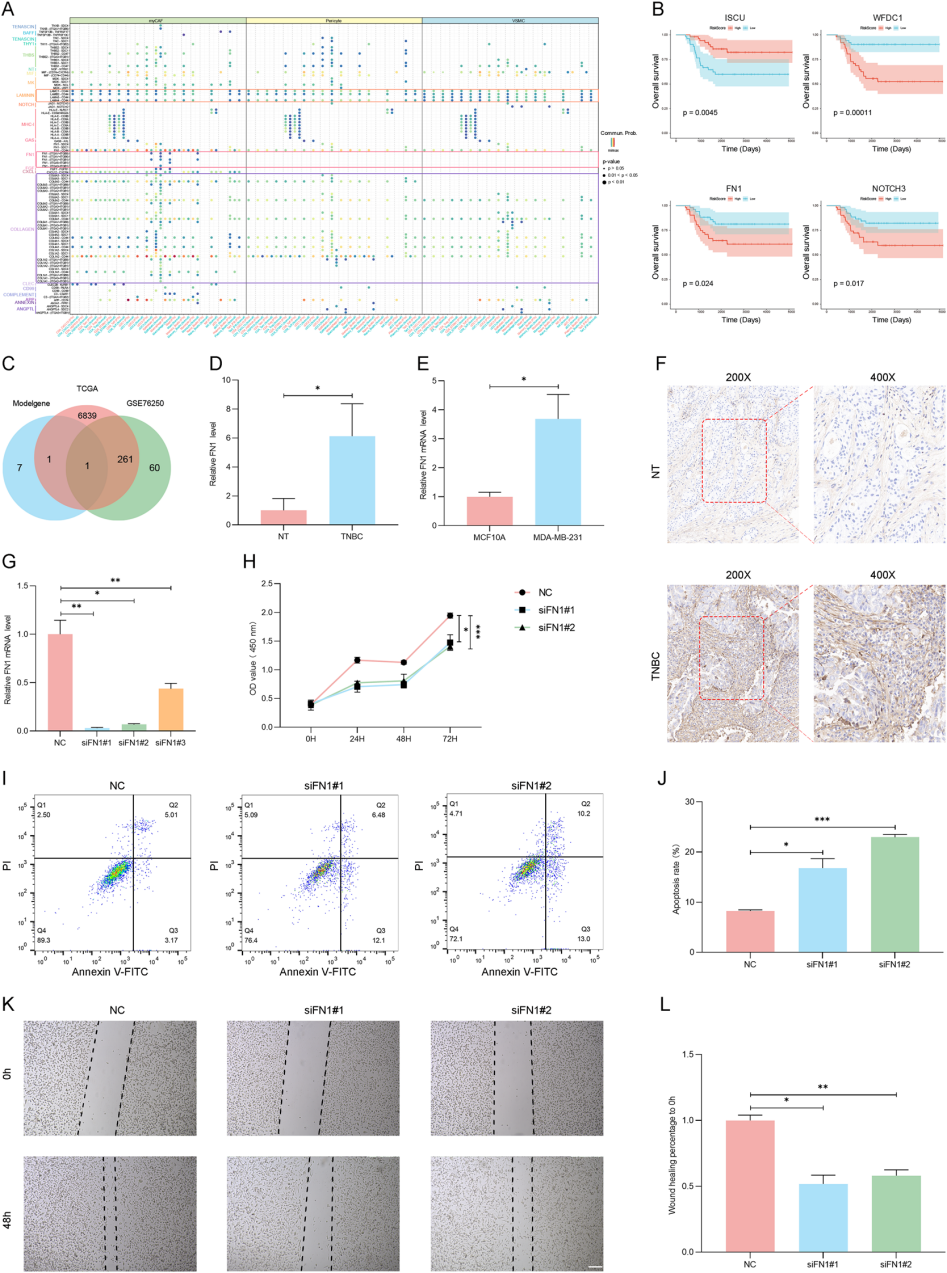
The FN1 gene promotes the malignant phenotype of TNBC. **(A)** Dot plot illustrates significant ligand-receptor interactions between myCAF, iCAF, and VSMC cells and other TME cell types. The X-axis represents receptor cell types, with red and blue colors distinguishing different tissue types—red for tumor tissue and blue for normal tissue. The Y-axis lists ligand-receptor interaction pairs. Ligand-receptor interaction pairs from different pathways are labeled in different colors (e.g., purple for the COLLAGEN pathway, red for the FN1 pathway, and orange for the LAMININ pathway). At the top, signaling-sending cells are labeled and color-coded (green for myCAF, yellow for Pericytes, and blue for VSMC). The dot color represents the probability of ligand-receptor interactions (Commun.Prob), with redder colors indicating stronger interaction intensity. The dot size reflects statistical significance, with larger dots indicating higher significance. **(B)** The Kaplan-Meier survival curves illustrate the survival differences of key genes (ISCU, WFDC1, FN1, and NOTCH3) between the high MVP risk groups and the low MVP risk groups. The X-axis represents follow-up time (days), while the Y-axis indicates the proportion of patients surviving at different time points. The curve colors distinguish between high- and low-expression groups for each gene, and the shaded areas represent the confidence interval (CI) for survival probability in each group. **(C)** Venn Diagram Showing the overlap of MVPRS Genes with Differentially Expressed Genes in TCGA-TNBC and GSE76250 datasets. **(D)** The mRNA expression levels of FN1 were measured by RT-qPCR in samples matched TNBC and adjacent normal tissues (NT) and normalized to GAPDH (P < 0.05). **(E)** The mRNA expression levels of FN1 were measured by RT-qPCR in MCF10A and MDA-MB-231 cell lines and normalized to GAPDH (P < 0.05). **(F)** FN1 expression was evaluated by IHC in TNBC tissues and adjacent normal tissues (NT) using an anti-FN1 antibody. Staining intensity was higher in TNBC tumor tissues, particularly in invasive regions, with predominant localization in the extracellular matrix and cytoplasm. The sampled regions included tumor core and invasive front areas for TNBC tissues, and corresponding normal epithelial regions in NT. Representative images at 200× and 400× magnification are shown. **(G)** The mRNA expression levels of FN1 were measured by RT-qPCR in MDA-MB-231 cells treated with FN1 siRNA or negative control (NC) siRNA and normalized to GAPDH (n = 3, *P < 0.05, **P < 0.01). **(H)** The CCK8 assay was performed in MDA-MB-231 cells treated with FN1 siRNA or NC siRNA (*P < 0.05, ***P < 0.001). **(I-J)** Apoptosis in MDA-MB-231 cells treated with FN1 siRNA or NC siRNA was detected by Annexin V-FITC/PI staining. Representative flow cytometry plots **(I)** and quantification **(J)** are shown (*P < 0.05, ***P < 0.001). **(K-L)** Scratch wound healing assays were conducted in MDA-MB-231 cells treated with FN1 siRNA or NC siRNA to assess the impact of FN1 on cell migration. Images were captured at 0 and 48 hours post-scratch, and migration distances were quantified using ImageJ software (version 1.52). Representative images of wound closure (K, scale bars: 200 μm) and corresponding quantification **(L)** demonstrate a significant reduction in migration capacity following FN1 knockdown (*P < 0.05, **P < 0.01). Data represent the mean ± SD of three independent experiments.

In the cell communication analysis, we observed that compared to the normal group, TNBC patients exhibited significantly enhanced signalling of the LAMININ, FN1, and COLLAGEN pathways in myCAF, VSMC, and Pericyte cells. (**Figure 9A**). This suggested that these three cell types may play a crucial role in the initiation and progression of TNBC through these pathways.

Furthermore, among the nine genes included in MVPRS, we observed that four genes were significantly associated with TNBC prognosis (*P* < 0.05): *WFDC1*, *ISCU*, *FN1*, and *NOTCH3*. Kaplan-Meier survival analysis revealed that high ISCU expression correlated with improved prognosis in TNBC patients, with significantly longer overall survival (OS) in the high-expression group compared to the low-expression group (HR = 0.3209, P = 0.0045). The five-year survival rate was 85.79% for high-expression patients versus 58.71% for low-expression patients. Conversely, high expression of WFDC1, FN1, and NOTCH3 was associated with poor prognosis, with significantly shorter OS and faster survival decline over time (WFDC1: HR = 4.529, P = 0.00011; FN1: HR = 2.672, P = 0.024; NOTCH3: HR = 2.3554, P = 0.017). The five-year survival rates for high-expression patients were 55.69%, 62.64%, and 63.46%, respectively, compared to 88.53%, 81.7%, and 80.58% in the low-expression group(**Figure 9B**). Subsequently, we conducted an intersection analysis between the genes in the model, the differentially expressed genes in the TCGA-TNBC dataset, and those in the GSE76250 dataset, ultimately identifying a single gene——*FN1* (**Figure 9C**, **Supplementary Table S17**, S18). In light of the prognostic results, we speculated that FN1 may play a critical role in the initiation and progression of TNBC.

To evaluate the impact of *FN1* on TNBC, we examined the expression levels of *FN1* in TNBC tissues and cell lines. The results showed that FN1 was significantly upregulated in TNBC tumor tissues and MDA-MB-231 cells compared to adjacent normal tissues and normal breast epithelial cells, as confirmed by RT-qPCR (P < 0.05) and IHC (**Figures 9D-F**). Notably, IHC staining revealed that FN1 was primarily distributed in the extracellular matrix and cytoplasm of TNBC tissues, with stronger staining observed in invasive regions (**Figure 9F**). To further investigate the functional impact of FN1, we performed knockdown experiments in MDA-MB-231 cells. FN1 silencing led to a significant reduction in mRNA levels (**Figure 9G**), suppressed cell proliferation as assessed by CCK8 assay (P < 0.01, **Figure 9H**), and induced apoptosis, as indicated by Annexin V-FITC/PI staining (P < 0.001, **Figures 9I, J**). Furthermore, FN1 knockdown markedly impaired cell migration, as demonstrated by wound healing assays (P < 0.05, **Figures 9K, L**). These findings suggested that FN1 plays a critical role in TNBC progression by promoting tumor cell proliferation, survival, and migration.

## 4 Discussion

This study provides an in-depth analysis of the TME in TNBC, focusing specifically on stromal cell subgroups (myCAF, VSMC, and pericyte) that exhibit high-frequency cell communication, strong interaction intensity, and are closely related to TNBC prognosis and response to immunotherapy. By integrating single-cell RNA sequencing data with transcriptomic data, we identified a gene set closely associated with these three cell types (MVPRGs) and developed a nine-gene prognostic signature (MVPRS). This model showed strong predictive accuracy for prognosis and immunotherapy response across multiple datasets.

In the tumor microenvironment (TME), different immune cells exhibit distinct functions, with some promoting tumor progression while others demonstrate anti-tumor activity. For example, M2 macrophages have been reported to contribute to tumor development through various mechanisms, including immune suppression, angiogenesis, neovascularization, stromal activation and remodeling (49–54). Similarly, tumor cells can secrete chemokines such as CXCL12, VEGF, and CXCL8/IL-8, attracting mast cells into the TME, where they become activated and release cytokines, chemokines, and angiogenic factors, further driving tumor progression (55, 56). Additionally, mast cells can promote tumor progression by releasing anti-inflammatory cytokines such as IL-10 and TGF-β, which suppress immune responses (57, 58). However, under specific conditions, mast cells may also exert anti-tumor effects, with their functions in the TME being regulated by multiple factors (59–61). In contrast, some immune cells play a crucial anti-tumor role in the TME. CD4^+^ Tem (CD4^+^ effector memory T cells) can recognize and bind to antigens on the surface of tumor cells to trigger an immune response. They enhance anti-tumor immunity by secreting IFN-γ, IL-2, IL-4 and IL-17, which activate cytotoxic T lymphocytes (CTLs), natural killer (NK) cells, and macrophages (62, 63). Effector CD8+ T cells (Teff) are the body’s primary effector cells and the key players in anti-tumor immunity. They can directly kill tumor cells through perforin and granzyme B (64). cDC2 activates effector T cells by secreting various cytokines (such as IL-10 and IL-23) and presenting antigens to CD4^+^ helper T cells (65, 66). NK cells can not only directly kill tumor cells but also recruit other immune cells and enhance adaptive immune responses of T cells and B cells by secreting various cytokines and chemokines (67). Naïve B cells express IgM and IgD antibodies that have not undergone somatic hypermutation on their surface. Upon encountering an antigen, they become activated, proliferate, and differentiate into effector B cells or memory B cells, exerting anti-tumor effects (68, 69). Naïve CD4^+^ T cells are immune-responsive precursor cells that have not yet encountered antigen stimulation and play a role in initiating and regulating immune responses. Upon activation through their specific T cell receptor (TCR), naïve CD4^+^ T cells can differentiate into Th1, Th2, Th17, Treg and follicular helper T cells (70–72). CD4^+^ memory T cells play a crucial role in preserving immune memory, which is essential for establishing effective tumor defense and preventing tumor recurrence (73). Studies have shown that the enrichment of resting CD4^+^ memory T cells is associated with benign prognosis in patients, while a high abundance of activated CD4^+^ memory T cells is closely linked to favorable prognosis and response to immunotherapy (74, 75). Additionally, γδ T cells have been widely observed in various tumor tissues, and their high abundance is generally associated with better prognosis. Their unique “stress-sensing” function enables them to recognize NKG2D ligands (NKG2DLs) such as MICA, which are expressed on malignant transformed cells. Through NKG2D-mediated signaling pathways, γδ T cells activate cytotoxic responses to eliminate abnormal cells. Therefore, γδ T cells are considered potent anti-tumor lymphocytes, playing a critical role in immune surveillance and anti-tumor responses (76–79). The primary function of M1 macrophages is to enhance local inflammatory responses and recruit immune cells by secreting pro-inflammatory cytokines such as IL-1, IL-6, IL-12, IL-23, and TNF-α, thereby forming a strong anti-tumor immune defense. Additionally, M1 macrophages have shown great potential in tumor immunity. Studies have demonstrated that M1 macrophages exert anti-tumor effects by secreting pro-inflammatory cytokines and utilizing direct cytotoxic mechanisms to inhibit tumor cell proliferation and survival. Moreover, they can activate T cells and further enhance anti-tumor immunity (49, 59, 70, 74–76, 80–82). Our results show that the low-risk group is significantly enriched in cell types with antitumor activity, while the high-risk group is notably enriched in tumor-promoting cell types. This suggests a potential mechanism for the better prognosis and immunotherapy response observed in the low-risk group. Moreover, functional enrichment analysis revealed significant differences between the high-risk and low-risk groups in biological processes related to immune responses, such as regulation of cytotoxicity, immune response modulation, and immune activation. The low-risk group exhibited markedly better immune activity. The hallmark pathways positively correlated with MVPRS were predominantly identified as oncogenic pathways, including ANGIOGENESIS, NOTCH SIGNALING, EPITHELIAL MESENCHYMAL TRANSITION, GLYCOLYSIS, MYOGENESIS, HYPOXIA, COAGULATION, MTORC1 SIGNALING and HEDGEHOG SIGNALING. Previous studies have shown that these pathways are frequently overactivated in various cancers. They are known to drive tumor cell proliferation, invasion, and metastasis, and are often linked to poor prognosis (83–86). In contrast, pathways primarily associated with immune responses, such as interferon response, inflammatory response, and the complement system, are negatively correlated with MVPRS (87–89). The differences in these pathways may account for the variations in immune therapy responses and prognoses between different risk groups. Targeting these aberrantly activated pathways could offer a potential strategy to suppress TNBC progression.

Our results showed that the prognostic signature, termed MVPRS, constructed by using characteristic genes of myCAF, VSMC, and pericyte, can predict whether patients will benefit from immunotherapy (11–13, 90). Stromal cells, such as myCAF, VSMC, and pericyte, significantly influence the effectiveness of immunotherapy in the tumor microenvironment by promoting tumor growth, immune suppression, and vascular remodeling. Myofibroblasts also suppress antitumor immune responses by secreting immunosuppressive factors such as TGF-β and IL-11, rendering the TME immunosuppressive (18). VSCM and pericytes further reduce immune cell infiltration and function by promoting aberrant tumor angiogenesis and a hypoxic microenvironment, thereby weakening the effectiveness of immunotherapy (13, 26, 38). These results further demonstrate that MVPRS can serve as an effective indicator for predicting immune therapy response, offering robust support for identifying potential beneficiaries of immunotherapy in clinical practice.

Our results showed that TNBC has a distinct communication pattern compared with normal tissue. In TNBC, signalling through the LAMININ, FN1, and COLLAGEN pathways was significantly enhanced in myCAF, VSMC, and pericyte cells. The LAMININ pathway involves laminin and its associated signaling mechanisms. Laminin, widely present in the cellular matrix of adult tissues, plays a critical role in cell anchoring. By binding to integrin receptors, it activates signaling pathways such as Ras/Raf/MAPK and RhoGAP, thereby regulating cell growth, proliferation, and migration. This pathway is essential for normal developmental processes in the body (91). Extensive evidence indicates that the aberrant expression of the LAMININ pathway is associated with various cancers, including colorectal cancer, liver cancer, lung cancer, and pancreatic cancer (92). Tumors regulate the PI3K/Akt signaling pathway through laminin to influence the proliferation, invasion, and metastatic phenotypes of tumor cells (93). Fibronectin, a key adhesion molecule in the extracellular matrix, plays a critical role in the FN1 pathway by binding to integrin receptors, thereby regulating cellular adhesion, migration, and proliferation. In tumors, the FN1 pathway is upregulated, and the interaction between FN1 and its receptors activates downstream signaling pathways, such as FAK and PI3K/Akt. This activation enhances the survival and invasive capabilities of tumor cells (94, 95). Under normal conditions, collagen binds to integrins, activating signaling pathways such as FAK/Src, PI3K/Akt, MEK/ERK, and Rho/ROCK, which promote processes like cytoskeletal rearrangement, cell survival, and proliferation. However, in the TME, the interaction between collagen and integrins accelerates tumor progression. For example, in fibrotic livers, the upregulation of integrin expression, upon binding with collagen, activates the PI3K/Akt/mTOR and FAK/ERK pathways, thereby promoting the invasion and growth of hepatocellular carcinoma (96). These studies suggest that these pathways may play a key role in the occurrence and progression of TNBC, and further related research is expected to find new targets (**Figure 9A**).

In the constructed nine-gene MVPRS model, *WFDC1*, *ISCU*, *FN1*, and *NOTCH3* demonstrated significant prognostic value. Specifically, ISCU was associated with favorable prognosis, whereas *WFDC1*, *FN1*, and *NOTCH3* were linked to poor prognosis. Subsequently, we performed an intersection analysis between these genes, the differentially expressed genes from the TCGA-TNBC dataset, and the differentially expressed genes from the GSE76250 dataset, ultimately identifying the *FN1* gene. The *FN1* gene encodes fibronectin, which is involved in cell adhesion and migration processes, including wound healing, blood coagulation, fibrosis, and metastasis (97). Wang H et al. identifies FN1 as an independent prognostic factor for OS and disease-free survival (DFS), and found that it is upregulated in gastric cancer tumors (98). FN1 is associated with biological characteristics essential for tumors, such as sustaining proliferative signalling, stimulating angiogenesis, promoting invasion and metastasis, and modulating antitumor immunity (99). Zhang XX et al. finds that *FN1* overexpression is associated with poor prognosis in breast cancer (100). As expected, our *in vitro* experiments confirmed that *FN1* expression in TNBC tissues and cell lines was significantly higher than in normal tissues and cell lines, and *FN1* downregulation inhibited the proliferation and migration phenotypes of TNBC cells while promoting cell apoptosis. Therefore, FN1 holds promise as a potential therapeutic target for TNBC.

Unlike other studies on prognostic signatures, we focused on cell populations within the TNBC microenvironment that exhibit frequent cell communication and strong interaction intensity, and are closely associated with TNBC prognosis. We constructed a prognostic signature that can predict the prognosis and immunotherapy response of TNBC patients. The strength of this study comes from the use of a robust machine learning framework, which guarantees the high reliability of the MVPRS in terms of prediction accuracy and clinical applicability. Meanwhile, we integrated scRNA-seq, extensive transcriptomic, and clinical data to gain a multidimensional understanding of the heterogeneity of the TNBC microenvironment. However, there are still some limitations in this study. Although we evaluated the predictive performance of the MVPRS signature in both the training and validation sets, further validation through large-scale, multi-center prospective studies is needed to confirm our findings. Additionally, further *in vitro* and *in vivo* experiments are required to gain a deeper understanding of the molecular mechanisms of *FN1* in TNBC progression, providing valuable theoretical foundations and practical references for TNBC research and treatment.

## 5 Conclusions

In this study, we constructed a prognostic model closely associated with stromal cell subpopulations (myCAF, VSMC, pericyte), which has the potential to serve as a promising tool for prognosis prediction and personalized medicine in TNBC patients. Additionally, we integrated single-cell transcriptomics and bulk transcriptomics approaches, providing new insights into the molecular mechanisms underlying TNBC initiation and progression. We also validated the expression and regulatory role of the key prognostic gene *FN1* in TNBC progression in TNBC cell lines. In summary, the findings of this study not only enhance our understanding of prognosis prediction and immunotherapy response in TNBC patients but also offer hope for therapeutic strategies targeting stromal cell subpopulations, providing a theoretical basis and reference for TNBC research and treatment.

## Data Availability

The original contributions presented in the study are included in the article/**Supplementary Material**. Further inquiries can be directed to the corresponding authors.
