# Supplementary material for: Construction of a stromal cell-related prognostic signature based on a 101-combination machine learning framework for predicting prognosis and immunotherapy response in triple-negative breast cancer
Source: Front Immunol. 2025 May 14;16:1544348. doi: 10.3389/fimmu.2025.1544348 (PMC12116347; doi:10.3389/fimmu.2025.1544348)
Supplement: Supplementary file 1 [file DataSheet1.docx]

Fig2.R

# scRNA

sce <- readRDS("~/my_prepare/lfr_finally/Breastcancer_EMBOJ/new_analyse_24_4_17/sce_primary_er_her2_tnbc_normal_lastFilter_24_4_17.rds")

sce <- NormalizeData(sce, normalization.method = "LogNormalize",

scale.factor = 10000)

GetAssay(sce,assay = "RNA")

sce <- FindVariableFeatures(sce,

selection.method = "vst", nfeatures = 3000)

sce <- ScaleData(sce)

sce <- RunPCA(object = sce, pc.genes = VariableFeatures(sce))

res.used <- 1.0

ElbowPlot(sce,ndims = 50)

sce <- FindNeighbors(sce, dims = 1:20)

sce <- FindClusters(object = sce, verbose = T, resolution = res.used)

# sce <- FindClusters(object = sce, verbose = T, resolution = 0.5)

set.seed(123)

sce <- RunUMAP(object = sce, dims = 1:20, do.fast = TRUE)

sce <- RunTSNE(object = sce, dims = 1:20, do.fast = TRUE)

# DimPlot(sce,reduction = "umap",label=T,raster=FALSE)

# DimPlot(sce,reduction = "umap",label=T, group.by = "orig.ident")

table(sce@meta.data$seurat_clusters)

p1=DimPlot(sce,group.by = "seurat_clusters",label = T,raster=FALSE)

p2=DimPlot(sce,group.by = "orig.ident",label = T,raster=FALSE)+NoLegend()

p3=DimPlot(sce,group.by = "Tissue",label = T,raster=FALSE)

p1+p2+p3

p1=DimPlot(sce,group.by = "seurat_clusters",label = T,raster=FALSE,reduction = "tsne")+NoLegend()

p2=DimPlot(sce,group.by = "orig.ident",label = T,raster=FALSE,reduction = "tsne")+NoLegend()

p3=DimPlot(sce,group.by = "Tissue",label = T,raster=FALSE,reduction = "tsne")

p1+p2+p3

# 注释看看

all_nonimmune=list(

immune=c("PTPRC"),

tcell=c("CD3D","CD3E"),

nk=c("NCAM1","GNLY","NKG7","KLRD1"),

Stromal=c("DCN","COL1A2","COL1A1","LUM","PDGFRA","PDGFRB"

),

Plasma_bcell=c("MZB1","IGHG4"),

Myepithelials=c("KRT14","ACTA2"),

myeloid=c("CD68","CD163","LYZ","SPP1","CST3",

"LST1","LILR82",

"C1QC","C1QA",

"TREM2"),

epi=c("CD24","EPCAM","KRT19","KRT7","KRT8","KRT18"

# ,"KRT5"

),

edo=c("PECAM1","VWF","ENG","CDH5","PLVAP"), # 内皮

CD20_bcell=c("MS4A1","CD79A")

)

th=theme(axis.text.x = element_text(angle = 90,

vjust = 0.5, hjust=0.5))

p <- DotPlot(sce, features = all_nonimmune,

assay='RNA')+ th

p

# 注释一下

celltype=data.frame(ClusterID=0:48,

celltype="Epithelials")

celltype[celltype$ClusterID %in% c(3,32),2]='Edothelials' #

celltype[celltype$ClusterID %in% c(0,8,39,41),2]='T_NK' #

celltype[celltype$ClusterID %in% c(23),2]='Naive_Bcells' #

celltype[celltype$ClusterID %in% c(22,48),2]='Plasma_Bcells' #

celltype[celltype$ClusterID %in% c(14,16,38),2]='Myeloids'

celltype[celltype$ClusterID %in% c(40),2]='Mast'

celltype[celltype$ClusterID %in% c(6,9,13,30,46),2]='Fibroblasts'

celltype[celltype$ClusterID %in% c(34),2]='Myepithelials' # 即epi+fib

celltype[celltype$ClusterID %in% c(19),2]='Undefined'

celltype[celltype$ClusterID %in% c(1,2,4,5,7,10,11,12,15,17,18,20,21,24,25,26,27,28,29,

31,33,35,36,37,42,43,44,45,47),2]='Epithelials'

head(celltype)

celltype

table(celltype$celltype)

sce@meta.data$firstannotation = "NA"

for(i in 1:nrow(celltype)){

sce@meta.data[which(sce@meta.data$seurat_clusters == celltype$ClusterID[i]),'firstannotation'] <- celltype$celltype[i]}

table(sce@meta.data$firstannotation)

DimPlot(sce, reduction = "tsne",cols = mycolor2,group.by = "firstannotation", label = TRUE,pt.size = 0.5,label.box = T,raster = FALSE)

# plot1cell

library(plot1cell)

#

sce <- readRDS("~/my_prepare/lfr_finally/Breastcancer_EMBOJ/new_analyse_24_4_17/sce_primary_er_her2_tnbc_normal_第一次注释_24_4_18.rds")

load("~/my_prepare/lfr_finally/Breastcancer_EMBOJ/new_analyse_24_4_17/phe_大群与T_NK_myeloids_Bcell整合_24_6_17.Rdata")

sce@meta.data=merge_meta

#

head(sce@meta.data)

#

DimPlot(sce, reduction = "tsne",group.by = "firstannotation_modify", label = TRUE,pt.size = 0.5,label.box = T,raster = FALSE)

DimPlot(sce, reduction = "umap",group.by = "firstannotation_modify", label = TRUE,pt.size = 0.5,label.box = T,raster = FALSE)

DimPlot(sce, reduction = "umap",group.by = "Tissue", label = TRUE,pt.size = 0.5,label.box = T,raster = FALSE)

#

head(sce,2)

Idents(sce)=sce$firstannotation_modify

###Check and see the meta data info on your Seurat object

colnames(sce@meta.data)

# turn tsne/umap

source("../出图_24_9_3/plot_circlize.R")

###Prepare data for ploting

circ_data <- prepare_circlize_data(sce, scale = 0.8 )

colnames(circ_data)

#

set.seed(1234)

#

#设置需要的颜色

mycolors <-c('#E64A35','#4DBBD4' ,'#01A187' ,'#6BD66B','#3C5588' ,'#F29F80' ,

'#7F2268','#91D0C1','#bebcdf')

mycolors <-c('#E64A35','#4DBBD4' ,'#01A187' ,'#6BD66B','#3C5588' ,'#F29F80' ,

'#7F2268','#91D0C1','#FFCC4F')

cluster_colors<-mycolors

group_colors<-rand_color(length(names(table(sce$Tissue)))) #分组

# "#145779FF" "#8A04D0FF" "#467F08FF" "#B6D028FF"

# "#A008CAFF" "#CD590FFF" "#DF83D8FF" "#DFC5FBFF"

rep_colors<-rand_color(length(names(table(sce$orig.ident))))

###plot and save figures

plot_circlize(circ_data,do.label = T, pt.size = 0.02,

col.use = cluster_colors ,bg.color = '#f9f9f9e5', #'#f8f2e4'

kde2d.n = 200, repel = T, label.cex = 1.2)

add_track(circ_data, group = "Tissue", colors = group_colors, track_num = 2)

add_track(circ_data, group = "orig.ident",colors = rep_colors, track_num = 3)

# 修改plot_circlize.R脚本可以达到修改字体大小，以及对tsne进行画图，默认是umap

# 对tsne结果进行可视化

# 添加亚群可视化结果

sce_fib <- readRDS("~/my_prepare/lfr_finally/Breastcancer_EMBOJ/new_analyse_24_4_17/sce_primary_er_her2_tnbc_normal_fib_24_4_19.rds")

DimPlot(sce_fib, reduction = "tsne",group.by = "fibannotation", label = TRUE,pt.size = 0.5,label.box = T,raster = FALSE)

DimPlot(sce_fib, reduction = "umap",group.by = "fibannotation", label = TRUE,pt.size = 0.5,label.box = T,raster = FALSE)

sce_bcell <- readRDS("~/my_prepare/lfr_finally/Breastcancer_EMBOJ/new_analyse_24_4_17/sce_bcell_all_24_6_16.rds")

#

DimPlot(sce_bcell, reduction = "tsne",group.by = "bcellannotation", label = TRUE,pt.size = 0.5,label.box = T,raster = FALSE)

DimPlot(sce_bcell, reduction = "umap",group.by = "bcellannotation", label = TRUE,pt.size = 0.5,label.box = T,raster = FALSE)

sce_NK <- readRDS("~/my_prepare/lfr_finally/Breastcancer_EMBOJ/new_analyse_24_4_17/sce_从T里面分出NK_24_6_16.rds")

#

DimPlot(sce_NK, reduction = "tsne",group.by = "t_nkcellannotation", label = TRUE,pt.size = 0.5,raster = FALSE)

# 这边需要把T细胞注释的结果映射到T_NK注释中

load("~/my_prepare/lfr_finally/Breastcancer_EMBOJ/new_analyse_24_4_17/phe_T_NK整合_24_6_17.Rdata")

sce_NK@meta.data=merge_meta

DimPlot(sce_NK, reduction = "tsne",group.by = "t_nk_integrated_6_17", label = TRUE,pt.size = 0.5,raster = FALSE)

DimPlot(sce_NK, reduction = "umap",group.by = "t_nk_integrated_6_17", label = TRUE,pt.size = 0.5,raster = FALSE)

#

sce_myeloids <- readRDS("~/my_prepare/lfr_finally/Breastcancer_EMBOJ/new_analyse_24_4_17/sce_myeloids_24_9_5.rds")

DimPlot(sce_myeloids, reduction = "tsne",group.by = "myeloidannotation", label = TRUE,pt.size = 0.5,raster = FALSE)

DimPlot(sce_myeloids, reduction = "umap",group.by = "myeloidannotation", label = TRUE,pt.size = 0.5,raster = FALSE)

# 组成list

sub.celltype_list=list(sce_fib,sce_bcell,sce_NK,sce_myeloids)

names(sub.celltype_list)=c("Fib","Bcell","T_NK","Myeloids")

save(sub.celltype_list,file = '../出图_24_9_3/fig1_绘图需要.Rdata')

# 添加四周亚型umap图

my36colors <-c('#E5D2DD', '#53A85F', '#F1BB72', '#F3B1A0', '#D6E7A3', '#57C3F3', '#476D87',

'#E95C59', '#E59CC4', '#AB3282', '#23452F', '#BD956A', '#8C549C', '#585658',

'#9FA3A8', '#E0D4CA', '#5F3D69', '#C5DEBA', '#58A4C3', '#E4C755', '#F7F398',

'#AA9A59', '#E63863', '#E39A35', '#C1E6F3', '#6778AE', '#91D0BE', '#B53E2B',

'#712820', '#DCC1DD', '#CCE0F5', '#CCC9E6', '#625D9E', '#68A180', '#3A6963',

'#968175'

)

###Fibroblast subtypes

Fibroblast <- sub.celltype_list$Fib

Idents(Fibroblast) <- "fibannotation"

subcolors <- my36colors[1:nlevels(Fibroblast)]

#subcolors <- c('#bff542','#83f78f','#EBA1A2','#D70016','#eab3fc','#83b1f7','#D70016','#eab3fc','#83b1f7')

Fibroblast_meta<-get_metadata(Fibroblast, color = subcolors)

Fibroblast_meta %>%

dplyr::group_by(fibannotation) %>%

summarise(x = median(x),y = median(y)) -> centers_Fib

points(Fibroblast_meta$x*0.32-1.3,Fibroblast_meta$y*0.32-0.74, pch = 19, col = alpha(Fibroblast_meta$Colors,0.5), cex = 0.1); # x减的越小，越靠右边,y减的越小，越靠上

text(centers_Fib$x*0.32-1.3,centers_Fib$y*0.32-0.74, labels=centers_Fib$fibannotation,

cex = 0.8 # 字体大小

, col = 'black')

# 注意这里的subcolors 可以自定义，也可以每次都使用my36colors 中的颜色，但是一定要注意以下2点

#T subtypes

T.sub <- sub.celltype_list$T_NK

Idents(T.sub) <- "t_nk_integrated_6_17"

subcolors <- my36colors[1:nlevels(T.sub)]

T_meta<-get_metadata(T.sub, color = subcolors)

T_meta %>%

dplyr::group_by(t_nk_integrated_6_17) %>%

summarise(x = median(x = x),y = median(x = y)) -> centers_T

points(T_meta$x*0.32+1.2,T_meta$y*0.32+0.73, pch = 19, col = alpha(T_meta$Colors,0.5), cex = 0.1);

text(centers_T$x*0.32+1.2,centers_T$y*0.32+0.73, labels=centers_T$t_nk_integrated_6_17, cex = 0.6, col = 'black')

#Myeloid subtypes

Myeloid.sub <- sub.celltype_list$Myeloids

Idents(Myeloid.sub)="myeloidannotation"

my36colors <-c('#E5D2DD', '#53A85F', '#F1BB72', '#F3B1A0', '#D6E7A3', '#57C3F3', '#476D87',

'#E95C59', '#E59CC4', '#AB3282', '#23452F', '#BD956A', '#8C549C', '#585658',

'#9FA3A8', '#E0D4CA', '#5F3D69', '#C5DEBA', '#58A4C3', '#E4C755', '#F7F398',

'#AA9A59', '#E63863', '#E39A35', '#C1E6F3', '#6778AE', '#91D0BE', '#B53E2B',

'#712820', '#DCC1DD', '#CCE0F5', '#CCC9E6', '#625D9E', '#68A180', '#3A6963',

'#968175'

)

subcolors <- my36colors[1:nlevels(Myeloid.sub)]

# subcolors <- c('#D6E7A3','#E5D2DD', '#53A85F', '#F1BB72','#57C3F3', '#F3B1A0', '#476D87')

Myeloid_meta<-get_metadata(Myeloid.sub, color = subcolors)

Myeloid_meta %>%

dplyr::group_by(myeloidannotation) %>%

summarise(x = median(x = x),y = median(x = y)) -> centers_Mye

points(Myeloid_meta$x*0.32-1.2,Myeloid_meta$y*0.32+0.73, pch = 19, col = alpha(Myeloid_meta$Colors,0.5), cex = 0.1);

text(centers_Mye$x*0.32-1.2,centers_Mye$y*0.32+0.73, labels=centers_Mye$myeloidannotation, cex = 0.6, col = 'black')

##B subtype

Epi.sub <- sub.celltype_list$Bcell

Idents(Epi.sub)="bcellannotation"

subcolors <- my36colors[1:nlevels(Epi.sub)]

Epi_meta<-get_metadata(Epi.sub, color = subcolors)

Epi_meta %>%

dplyr::group_by(bcellannotation) %>%

summarise(x = median(x = x),y = median(x = y)) -> centers_Epi

points(Epi_meta$x*0.3+1.2,Epi_meta$y*0.3-0.73, pch = 19, col = alpha(Epi_meta$Colors,0.5), cex = 0.1);

text(centers_Epi$x*0.3+1.2,centers_Epi$y*0.3-0.73, labels=centers_Epi$bcellannotation, cex = 0.6, col = 'black')

#

# 3 ，添加四周umap的title 和 track的legend

#

# （1）添加，优化四周umap的title ，注意位置和大小

title_text <- function(x0, y0, x1, y1, text, rectArgs = NULL, textArgs = NULL) {

center <- c(mean(c(x0, x1)), mean(c(y0, y1)))

do.call('rect', c(list(xleft = x0, ybottom = y0, xright = x1, ytop = y1), rectArgs))

do.call('text', c(list(x = center[1], y = center[2], labels = text), textArgs))

}

title_text(x0 = -1.35, x1 = -1.05, y0 = -1.06, y1=-1, text = 'Stromal',

rectArgs = list(border='#F9F2E4',lwd=0.5),

textArgs = list(col='black',cex = 1))

title_text(x0 = 1.05, x1 = 1.35, y0 = -1.06, y1=-1, text = 'Bcells',

rectArgs = list(border='#F9F2E4',lwd=0.5),

textArgs = list(col='black',cex = 1))

title_text(x0 = -1.35, x1 = -1.05, y0 = 1.06, y1=1, text = 'Myeloids',

rectArgs = list(border='#F9F2E4',lwd=0.5),

textArgs = list(col='black',cex = 1))

title_text(x0 = 1.05, x1 = 1.35, y0 = 1.06, y1=1, text = 'T_NK cells',

rectArgs = list(border='#F9F2E4',lwd=0.5),

textArgs = list(col='black',cex = 1))

# （2）添加track的legend

#plot group#

col_use<-c('#00288A','#DD001F','#84D000','#00CB47','#947F00','#006234')

col_use=c('#E64A35','#4DBBD4' ,'#01A187' ,'#6BD66B','#3C5588' ,'#F29F80' ,

'#7F2268','#91D0C1','#FFCC4F')

# mycolors <-c('#E64A35','#4DBBD4' ,'#01A187' ,'#6BD66B','#3C5588' ,'#F29F80' ,

# '#7F2268','#91D0C1','#FFCC4F')

cc<-get_metadata(sce, color = col_use)

cc %>%

dplyr::group_by(Tissue) %>%

summarise(x = median(x = x),y = median(x = y)) -> centers

# group_colors<-rand_color(length(names(table(sce$Tissue)))) #分组 ,颜色前面搞过了

col_group<-c("#145779FF","#8A04D0FF","#467F08FF","#B6D028FF")

lgd_points = Legend(labels = names(table(cc$Tissue)), type = "points",

title_position = "topleft",

title = "Group",

title_gp = gpar(col='black',fontsize = 7, fontface='bold'),

legend_gp = gpar(col = col_group),

labels_gp = gpar(col='black',fontsize = 5),

grid_height = unit(2, "mm"),

grid_width = unit(2, "mm"),

background = col_group)

draw(lgd_points, x = unit(15, "mm"), y = unit(50, "mm"),

just = c("right", "bottom"))

fig2_plot_circlize.R

#%%%%%%%%%%%%%%%%%%%%%%%%%%%%%%%%%%%%%%%%%%%%%%%%%%%%%%%%%%%%%%%%%%%%%%%%%%%%%%%

# Functions

#%%%%%%%%%%%%%%%%%%%%%%%%%%%%%%%%%%%%%%%%%%%%%%%%%%%%%%%%%%%%%%%%%%%%%%%%%%%%%%%

#' Convert coordinates

#'

#' This function converts the Cartesian coordinates to Polar coordinates.

#' Input data can be the coordinates from tSNE or UMAP. It outputs a matrix with

#' polar coordinates.

#'

#' @param coord_data Cartesian coordinates from tSNE, UMAP, etc.

#' @param zoom Value from c(0,1) to adjust the coordinates.

#' @return A matrix with polar coordinates

#' @export

transform_coordinates <- function(

coord_data,

zoom

){

center_data<-coord_data-mean(c(min(coord_data),max(coord_data)))

max_data<-max(center_data)

new_data<-center_data*zoom/max_data

new_data

}

#' Get metadata from a Seurat object

#'

#' This function extracts the metadata from a Seurat object and transforms the

#' UMAP/tSNE coordinates.

#'

#' @param seu_obj SeuratObject

#' @param reductions reductions methods, e.g."umap" or "tsne".

#' @param color Colors assigned to the cell clusters

#' @param coord_scale value from c(0,1) to adjust the UMAP/tSNE coordinates.

#' @return A metadata dataframe

#' @export

get_metadata <- function(

seu_obj,

reductions = "umap",

coord_scale = 0.8,

color

){

metadata<-seu_obj@meta.data

metadata$Cluster<-seu_obj@active.ident

metadata$dim1<-as.numeric(seu_obj[[reductions]]@cell.embeddings[,1])

metadata$dim2<-as.numeric(seu_obj[[reductions]]@cell.embeddings[,2])

metadata$x<-transform_coordinates(metadata$dim1, zoom = coord_scale)

metadata$y<-transform_coordinates(metadata$dim2, zoom = coord_scale)

color_df<-data.frame(Cluster=levels(seu_obj), Colors=color)

cellnames<-rownames(metadata)

metadata$cells<-rownames(metadata)

metadata<-merge(metadata, color_df, by='Cluster')

rownames(metadata)<-metadata$cells

metadata<-metadata[cellnames,]

metadata

}

#' Make count matrix for the selected markers

#'

#' This function labels the cells based on their expression levels of the selected

#' marker genes.

#'

#' @param seu_obj SeuratObject

#' @param features Selected marker genes

#' @return A dataframe with cells labeled by marker genes

#' @export

mk_marker_ct <- function(

seu_obj,

features

){

dat <- Seurat::FetchData(seu_obj, vars = features)

ori_names <- rownames(dat)

zero_ct <- dat[rowSums(dat)==0,]

non_zero <- dat[rowSums(dat)!=0,]

max_genes <- colnames(non_zero)[max.col(non_zero,ties.method="first")]

non_zero <- data.frame(cells=rownames(non_zero), genes=max_genes)

zero_ct <- data.frame(cells=rownames(zero_ct), genes='No_expr')

all_cells <- rbind(non_zero, zero_ct)

rownames(all_cells) <- all_cells$cells

all_cells <- all_cells[ori_names,]

all_cells

}

#' Create a dataframe for color mapping

#'

#' This function assigns a color for each value in a vector

#'

#' @param group Group to be assigned color

#' @return A dataframe with colors assigned to groups

#' @export

mk_color_table <- function(group){

n=length(group)

colors=scales::hue_pal()(n)

color_table <- data.frame(group, colors)

color_table

}

#' Order the cells from each cluster

#'

#' This function orders the cells from each cluster by giving a value from

#' 1 to max

#' @param dat Data input.

#' @return An vector with ordered cells

#' @export

cell_order <- function(dat){

celltypes <- names(table(dat$Cluster))

new_dat <- list()

for (i in 1:length(celltypes)){

dat$Cluster<-as.character(dat$Cluster)

dat1<-dat[dat$Cluster==celltypes[i],]

dat1$x_polar<-1:nrow(dat1)

new_dat[[i]]<-dat1

}

new_dat<-do.call('rbind', new_dat)

new_dat

}

#' Create a segment for each element in a group

#'

#' This function creates a segment for each element within a group

#' @param dat Data input.

#' @param group The group name

#' @return An vector with ordered cells

#' @export

get_segment <- function(

dat,

group

){

dat<-dat[order(dat[,group],decreasing = F), ]

rownames(dat)<-1:nrow(dat)

dat<-dat[!duplicated(dat[,group]),]

dat_seg<-as.integer(rownames(dat))

dat_seg

}

#' Prepare circlize data for plotting

#'

#' This function creates a segment for each element within a group

#' @param seu_obj Seurat object

#' @param scale Scale factor to zoom in our zoom out the tSNE/UMAP proportionally

#' @return A data frame for plotting

#' @export

prepare_circlize_data <- function(

seu_obj,

scale =0.8

){

celltypes<-levels(seu_obj)

cell_colors <- scales::hue_pal()(length(celltypes))

data_plot <- get_metadata(seu_obj, color = cell_colors, coord_scale = scale)

data_plot <- cell_order(data_plot)

data_plot$x_polar2 <- log10(data_plot$x_polar)

data_plot

}

#' Generate a circlize plot outside the tSNE/UMAP

#'

#' This function generates a circlize plot outside the tSNE/UMAP

#'

#' @param data_plot Data frame prepared by the prepare_circlize_data function

#' @param do.label Whether to label the clusters

#' @param contour.levels Which contour line to be drawn on the plot. Value: 0-1

#' @param bg.color Canvas background color

#' @param label.cex Label font size

#' @param pt.size Point size of the graph

#' @param kde2d.n Number of grid points in each direction. A kde2d parameter

#' @param contour.nlevels Total number of levels in contour

#' @param col.use Colors used to label the cell type

#' @param repel Whether or not to repel the cell type names on umap

#' @return Return a circlize plot

#' @export

plot_circlize <- function(

data_plot,

do.label = T,

contour.levels = c(0.2,0.3),

pt.size = 0.5,

kde2d.n = 1000,

contour.nlevels = 100,

bg.color='#F9F2E4',

col.use=NULL,

label.cex = 0.5,

repel=FALSE

) {

data_plot %>%

dplyr::group_by(Cluster) %>%

summarise(x = median(x = x), y = median(x = y)) -> centers

z <- MASS::kde2d(data_plot$x, data_plot$y, n=kde2d.n)

celltypes<-names(table(data_plot$Cluster))

cell_colors <- scales::hue_pal()(length(celltypes))

if(!is.null(col.use)){

cell_colors=col.use

col_df<-data.frame(Cluster=celltypes, color2=col.use)

cells_order<-rownames(data_plot)

data_plot<-merge(data_plot, col_df, by="Cluster")

rownames(data_plot)<-data_plot$cells

data_plot<-data_plot[cells_order,]

data_plot$Colors<-data_plot$color2

}

circos.clear()

par(bg = bg.color)

circos.par(cell.padding=c(0,0,0,0), track.margin=c(0.01,0),"track.height" = 0.01, gap.degree =c(rep(2, (length(celltypes)-1)),12),points.overflow.warning=FALSE)

circos.initialize(sectors = data_plot$Cluster, x = data_plot$x_polar2)

circos.track(data_plot$Cluster, data_plot$x_polar2, y=data_plot$dim2, bg.border=NA,panel.fun = function(x, y) {

circos.text(CELL_META$xcenter,

CELL_META$cell.ylim[2]+ mm_y(4),

CELL_META$sector.index,

cex=0.7, col = 'black', facing = "bending.inside", niceFacing = T)

circos.axis(labels.cex = 0.3, col = 'black', labels.col = 'black')

})

for(i in 1:length(celltypes)){

dd<-data_plot[data_plot$Cluster==celltypes[i],]

circos.segments(x0 = min(dd$x_polar2), y0 = 0, x1 = max(dd$x_polar2), y1 = 0, col = cell_colors[i], lwd=3, sector.index = celltypes[i])

}

text(x = 1, y=0.1, labels = "Cluster", cex = 0.4, col = 'black',srt=-90)

points(data_plot$x,data_plot$y, pch = 19, col = alpha(data_plot$Colors,0.2), cex = pt.size);

contour(z, drawlabels=F, nlevels= 100, levels = contour.levels,col = '#ae9c76', add=TRUE)

if(do.label){

if(repel){

textplot(x=centers$x, y=centers$y, words = centers$Cluster,cex = label.cex, new = F,show.lines=F)

} else {

text(centers$x,centers$y, labels=centers$Cluster, cex = label.cex, col = 'black')

}

}

}

#' Add tracks to the circlize plot

#'

#' This function allows users to add more tracks onto the circlize plot

#' @param data_plot Data for circlize plot

#' @param group The group to be shown on the new track

#' @param colors Color palette to color the group

#' @param track_lwd The width of the track. Default:3

#' @param track_num Which number this track is? Value is integer and starts with 2 for the 2nd track, track_num=3 for the 3rd track, etc...

#' @return A new circlize track adding to the current circlize plot

#' @export

add_track <- function(

data_plot,

group,

track_num,

track_lwd = 3,

colors = NULL

){

if(track_num<2){

stop("The first track is the cluster track. Please change the track_num to a value greater than 1")

}

circos.track(data_plot$Cluster, data_plot$x_polar2, y=data_plot$dim2, bg.border=NA)

celltypes<-names(table(data_plot$Cluster))

group_names<-names(table(data_plot[,group]))

if(is.null(colors)){

col_group = scales::hue_pal()(length(group_names))

} else {

col_group = colors

}

names(col_group) <- group_names

for(i in 1:length(celltypes)) {

data_plot_cl <- data_plot[data_plot$Cluster==celltypes[i],]

group_names_cl <- names(table(data_plot_cl[,group]))

col_group_cl <- as.character(col_group[group_names_cl])

dat_seg <- get_segment(data_plot_cl, group = group)

dat_seg2 <- c(dat_seg[-1]-1, nrow(data_plot_cl))

scale_factor<-max(data_plot_cl$x_polar2)/nrow(data_plot_cl)

dat_seg<-scale_factor*dat_seg

dat_seg2<-scale_factor*dat_seg2

circos.segments(x0 = dat_seg, y0 = 0, x1 = dat_seg2, y1 = 0, col = col_group_cl, sector.index = celltypes[i], lwd=track_lwd)

}

text(x = (1-0.03*(track_num-1)), y=0.1, labels = group, cex = 0.4, col = 'black',srt=-90)

}

#

Fig3.R

distribution_OR <- function(

meta_data,

celltype_column,

celltype_level = NULL,

condition_column,

condition_level = NULL

){

library(tidyverse)

colnames(meta_data)[which(colnames(meta_data) == celltype_column)] = "celltypE"

colnames(meta_data)[which(colnames(meta_data) == condition_column)] = "conditioN"

if(is.null(celltype_level)){

meta_data$celltypE = as.character(meta_data$celltypE)

meta_data$celltypE = factor(meta_data$celltypE,levels = sort(unique(meta_data$celltypE)))

} else {

meta_data$celltypE = factor(meta_data$celltypE,levels = celltype_level)

}

if(is.null(condition_level)) {

meta_data$conditioN = as.character(meta_data$conditioN)

meta_data$conditioN = factor(meta_data$conditioN,levels = sort(unique(meta_data$conditioN)))

} else {

meta_data$conditioN = factor(meta_data$conditioN,levels = condition_level)

}

count.dist = as.data.frame(table(meta_data$celltypE,meta_data$conditioN))

count.dist = spread(count.dist,key = Var2,value = Freq)

rownames(count.dist) = count.dist$Var1

count.dist$Var1 =NULL

count.dist = as.matrix(count.dist)

######################

#

library(data.table)

sum.col <- colSums(count.dist)

sum.row <- rowSums(count.dist)

count.dist.DT <- as.data.frame(count.dist)

setDT(count.dist.DT,keep.rownames=T)

count.dist.DT.melt <- data.table::melt(count.dist.DT,id.vars="rn")

colnames(count.dist.DT.melt) <- c("rid","cid","count")

library(plyr)

test.res <- as.data.table(

ldply(

seq_len(nrow(count.dist.DT.melt)), function(i){

this.row <- count.dist.DT.melt$rid[i]

this.col <- count.dist.DT.melt$cid[i]

this.c <- count.dist.DT.melt$count[i]

this.m <- matrix(

c(this.c,

sum.row[this.row]-this.c,

sum.col[this.col]-this.c,

sum(sum.col)-sum.row[this.row]-sum.col[this.col]+this.c),

ncol=2)

# # this celltype | not this celltype

# this tissue| a | c

#not this tissue| b | d

#阈值不固定

tmp.res <- fisher.test(this.m)

data.frame(

rid=this.row,

cid=this.col,

p.value=tmp.res$p.value,

OR=tmp.res$estimate # 约为 a*d / b*c

)

}

)

)

test.res <- merge(count.dist.DT.melt,test.res,by=c("rid","cid"))

test.res[,adj.p.value:=p.adjust(p.value,"BH")]

test.res = as.data.frame(test.res)

#############################################################

dist.p <- reshape2::dcast(test.res,rid~cid,value.var="p.value")

dist.OR <- reshape2::dcast(test.res,rid~cid,value.var="OR")

dist.p.adj <- reshape2::dcast(test.res,rid~cid,value.var="adj.p.value")

rownames(dist.p) = dist.p$rid

rownames(dist.OR) = dist.OR$rid

rownames(dist.p.adj) = dist.p.adj$rid

dist.p$rid=NULL

dist.OR$rid=NULL

dist.p.adj$rid=NULL

return(list(

"dist.p"=dist.p,

"dist.OR"=dist.OR,

"dist.p.adj"=dist.p.adj))

}

#### cellchat

sce=CreateSeuratObject(counts = sce@assays$RNA@counts,meta.data = sce@meta.data)

DefaultAssay(sce)

#

scRNAlist=sce

DimPlot(scRNAlist,reduction = "tsne",label = T)

table(scRNAlist@active.ident)

table(scRNAlist@meta.data$forth_integrated_bcell_6_17) ## 28种细胞类型

Idents(scRNAlist)=scRNAlist$forth_integrated_bcell_6_17

data.input <- scRNAlist@assays$RNA@data

table(scRNAlist@meta.data$Tissue)

# ER HER2 Normal TNBC

# 67668 46078 54614 21951

meta = scRNAlist@meta.data # a dataframe with rownames containing cell mata data

unique(meta$forth_integrated_bcell_6_17) # check the cell labels 也是28种细胞类型

# cell_type就是labels

colnames(meta)[17]="labels"

## 创建CellChat 对象

cellchat <- createCellChat(object = data.input, meta = meta, group.by = "labels")

cellchat <- addMeta(cellchat, meta = meta)

cellchat <- setIdent(cellchat, ident.use = "labels") # set "labels" as default cell identity

levels(cellchat@idents) # show factor levels of the cell labels

groupSize <- as.numeric(table(cellchat@idents)) # number of cells in each cell group

## 设置配体受体交互数据库

## 我们的数据库 CellChatDB 是一个手动整理的文献支持的配体受体在人和小鼠中的交互数据库。

## 小鼠中的CellChatDB包含2，021个经验证的分子相互作用，包括60%的自分泌/旁分泌信号相互作用、21%的细胞外基质（ECM）受体相互作用和19%的细胞-细胞接触相互作用

## 人的CellChatDB包含1，939个经验证的分子相互作用，包括61.8%的自分泌/旁分泌信号相互作用、21.7%的细胞外基质（ECM）受体相互作用和16.5%的细胞-细胞接触相互作用

CellChatDB <- CellChatDB.human

showDatabaseCategory(CellChatDB)

# Show the structure of the database

dplyr::glimpse(CellChatDB$interaction)

colnames(CellChatDB$interaction)

table(CellChatDB$interaction$annotation)

# Cell-Cell Contact ECM-Receptor Secreted Signaling

# 319 421 1199

CellChatDB$interaction[1:4,1:4]

head(CellChatDB$cofactor)

head(CellChatDB$complex)

head(CellChatDB$geneInfo)

unique(CellChatDB$interaction$annotation)

# [1] "Secreted Signaling" "ECM-Receptor"

# [3] "Cell-Cell Contact"

CellChatDB.use <- CellChatDB # simply use the default CellChatDB

# set the used database in the object

cellchat@DB <- CellChatDB.use ##

##预处理用于细胞通信分析的表达数据

cellchat <- subsetData(cellchat) # 取出表达数据

# cellchat <- subsetData(cellchat，features = NULL) # 这边可以选择感兴趣的基因

## 不想改成多线程

future::plan("multisession", workers = 1) # do parallel

cellchat <- identifyOverExpressedGenes(cellchat) ## 寻找高表达基因

cellchat <- identifyOverExpressedInteractions(cellchat) ## 寻找高表达的通路

future::plan("multisession", workers = 1)

# cellchat <- projectData(cellchat, PPI.human) ##投影倒PPI

cellchat <- projectData(cellchat, PPI.human)

## 我们这边是基因，但是有的时候基因跟蛋白质不一定对得上，还有一个可能是基因有的不会翻译成蛋白质

## 这边做了一个投影到PPI相当于做了一个翻译的过程

# 第二部分

## 细胞通信网络的推断

## 计算通信概率并推断cellchat网络

cellchat <- computeCommunProb(cellchat, raw.use = TRUE)

?computeCommunProb

## 这边raw.use 如果选FALSE,则这一步是用上面计算的蛋白质的结果计算

## 如果raw.use选TRUE，则这一步是用基因的矩阵计算

# Filter out the cell-cell communication if there are only few number of cells in certain cell groups

## 在computeCommunProb中，我们提供了一个选项，用于使用其他方法

## 默认情况下，CellChat 使用一种统计学上强大的均值方法，称为"trimean"，

## "trimean"大约是25%的截断平均值，这意味着如果一组表达细胞的百分比低于25%，则平均基因表达为零【为0的这些基因就不会再纳入细胞通讯的一个计算】。

## 要使用 10% 截断的平均值，用户可以设置type = "truncatedMean"和对trim = 0.1。

cellchat <- filterCommunication(cellchat, min.cells = 10) #去掉通讯数量很少的细胞

df.net <- subsetCommunication(cellchat) ##将细胞通讯预测结果以数据框的形式取出

# df.net <- subsetCommunication(cellchat) ##返回一个数据框架，该数据框架由配体/受体级别的所有推断细胞通信组成。设置slot.name = "netP"可以在信号通路级别访问推断的通信

# df.netp <- subsetCommunication(cellchat,solt.name="netP") ##只取通路，数据结构更简单

levels(cellchat@idents)

# 推测的每个"配体-受体"对的细胞间通信网络和每个"信号通路"分别存储在“net”和“netP”槽中。

## 在信号通路级别推断细胞-细胞通信

## CellChat 通过总结与每个信号通路相关的所有配体-受体相互作用的通信概率，来计算信号通路级别上的通信概率。

## NB：每个配体受体对和每个信号通路的推断细胞间通信网络分别存储在插槽"net"和"netP"中。

cellchat <- computeCommunProbPathway(cellchat)

# 计算整合的细胞通信网络

# 我们可以通过计算链接数或汇总通信概率来计算整合的细胞通信网络。用户还可以通过设置sources.use和targets.use`

cellchat <- aggregateNet(cellchat)

cellchat@netP$pathways ##看看有哪些信号通路

# [1] "COLLAGEN" "LAMININ" "FN1" "MIF" "CD99" "MK" "CXCL"

# [8] "APP" "THBS" "ANGPTL" "CCL" "NOTCH" "PTN" "TENASCIN"

# [15] "FGF" "MPZ" "GAS" "ESAM" "PERIOSTIN" "SEMA3" "GRN"

# [22] "CD46" "PDGF" "PROS" "GALECTIN" "ADGRE5" "ANGPT" "SELL"

# [29] "NT" "TWEAK" "TGFb" "CADM" "SEMA5" "JAM" "ITGB2"

# [36] "EGF" "HSPG" "BMP" "NEGR" "IGF" "AGRN"

head(cellchat@LR$LRsig) ##看看具体的配受体情况

# 我们还可以可视化整合的细胞通信网络。例如，使用圆图显示任意两个细胞组之间的相互作用次数或总交互强度（比重）。

groupSize <- as.numeric(table(cellchat@idents))

par(mfrow = c(1,2), xpd=TRUE) ##设置图片的一个布局

netVisual_circle(cellchat@net$count, vertex.weight = groupSize, weight.scale = T,

label.edge= F, title.name = "Number of interactions") ##互作的数量

netVisual_circle(cellchat@net$weight, vertex.weight = groupSize, weight.scale = T,

label.edge= F, title.name = "Interaction weights/strength") ##互作的强度

#

## 互作数量和重要性图

## 由于细胞通信网络复杂，

## 我们可以检查每个细胞组发送的信号。在这里，我们还控制参数edge.weight.max，以便我们可以比较不同网络之间的边缘权重。

#

# load("~/my_prepare/lfr_finally/Breastcancer_EMBOJ/emboj_预后模型/cellchat/cellchat_all/cellchat_all.Rdata") # 这是原来拿所有组计算的，包括er,her2,tnbc,normal

# KM

#

rm(list=ls())

gc()

#

#

load("~/my_prepare/lfr_finally/Breastcancer_EMBOJ/emboj_预后模型/ssgsea/new/cellMarker_ssGSEA_全部乳腺癌亚型_24_7_19.Rdata") # 制备的cellMarker

load("~/my_prepare/lfr_finally/Breastcancer_EMBOJ/emboj_预后模型/101机器学习/Mime1包/mydata/GSE58812_清洗好的data.Rdata")

expr=as.matrix(exp)

gsva_data <- gsva(expr,cellMarker, method = "ssgsea",abs.ranking = TRUE) # 默认参数kcdf = "Gaussian"，适用于对数转换的microarray、RNA-seq的log-CPMs、log-RPKMs或log-TPMs。当输入表达的矩阵是RNA-seq的raw Count时，这个参数应该设置为kcdf = "Poisson"。

a <- gsva_data %>% t() %>% as.data.frame()

results=a

# 用我们整理好的预后信息

head(cli)

# 使用sub函数提取冒号后面的值

cli$OS <- sub(".*: ", "", cli$characteristics_ch1.4)

cli$OS.time <- sub(".*: ", "", cli$characteristics_ch1.5)

#

cli=cli[,c(3,4)]

head(cli)

#

# sub 函数用于字符串替换。

# "*.: " 匹配任何字符直到最后一个冒号和空格。

# "" 替换匹配的部分为空字符串，即删除匹配的部分。

sur_data=cli

sur_data$OS.time=as.numeric(sur_data$OS.time)

sur_data$OS=as.numeric(sur_data$OS)

# #去掉生存信息不全或者生存时间小于30天的样本，样本纳排标准不唯一，且差别很大.

sur_data =sur_data[sur_data$OS.time >= 30,]

sur_data = sur_data[!(is.na(sur_data$OS.time)|is.na(sur_data$OS)),]

sur_data[1:4,1:2]

results[1:4,1:4]

results2=as.data.frame(results)

sur_data2=sur_data[rownames(sur_data) %in% rownames(results2),]

results3=results2[rownames(results2) %in% rownames(sur_data2),]

results3$bcr_patient_barcode=rownames(results3)

sur_data2$bcr_patient_barcode=rownames(sur_data2)

#

aimplot <- left_join(x=sur_data2,y=results3,by='bcr_patient_barcode')

save(aimplot,file = "../../../Breastcancer_EMBOJ/emboj_预后模型/ssgsea/new/aimplot_细胞评分结果.Rdata")

# 最佳分割点

colnames(aimplot)[7]="EMT_likeCAF"

res.cut <- surv_cutpoint(aimplot, time = "OS.time", event = "OS",

variables = c("Pericyte",

"myCAF","VSMC","CD8_STMN1",

"Macrophage","iCAF",

"EMT_likeCAF","Edothelials","cDC3" ,

"Plasma_Bcells","apCAF",

"Myepithelials","cDC2",

"cDC1","Memory_Bcells","CD4_Tem",

"CD4_Treg","Monocyte","Mast",

"CD8_Teff","CD4_CXCL13","NK",

"NAF","Tact_IFI6","Naive_Bcells",

"CD4_HSPA1A","pDC" )) #

summary(res.cut)

# 使用surv_categorize()函数根据最佳截断值对数据进行分组（高表达/低表达组）：

res.cut2 <- surv_categorize(res.cut)

head(res.cut2)

table(res.cut2$Pericyte)

table(res.cut2$myCAF)

table(res.cut2$VSMC)

table(res.cut2$CD8_STMN1)

# 配色

# RColorBrewer中的调色板

palette = "Set2" # Set1, Set2, Paired 等都是不错的选择

# 使用 ggsci 的科学期刊配色：

library(ggsci)

palette = "npg" # 使用 Nature 期刊风格配色

# 根据最佳截断点绘制生存曲线：

fit <- survfit(Surv(OS.time, OS) ~ Pericyte, data = res.cut2)

ggsurvplot(fit, data = res.cut2,

conf.int = TRUE, pval = TRUE,

surv.median.line = "hv",

risk.table = TRUE, palette = "hue")

ggsurvplot(fit, data = res.cut2,

conf.int = TRUE, pval = TRUE,

surv.median.line = "hv", # 中位生存线

# risk.table = TRUE,

palette = c("blue","purple")

)

ggsurvplot(fit, data = res.cut2,

conf.int = TRUE,

pval = TRUE,

surv.median.line = "hv",

# risk.table = TRUE,

# risk.table.height = 0.25, # 调整风险表高度

# risk.table.col = "strata", # 风险表按分层显示颜色

palette = "npg", # 配色方案

ggtheme = theme_minimal() # 使用干净的主题

)

#

#

Fig4.R

# 火山图

load("~/my_prepare/lfr_finally/Breastcancer_EMBOJ/emboj_预后模型/wgcna/new/inputgene_24_7_19.Rdata")

# 输入文件

all.markers = markers_genes %>% dplyr::filter(p_val<0.05)

#

# top5= all.markers %>% group_by(cluster) %>% top_n(n = 5, wt = avg_log2FC)

top10= all.markers %>% group_by(cluster) %>% top_n(n = 10, wt = avg_log2FC)

top10=as.data.frame(top10)

head(top10)

# p_val avg_log2FC pct.1 pct.2 p_val_adj cluster gene

# 1 0 3.413885 0.312 0.023 0 iCAF PLA2G2A

# 2 0 3.086529 0.576 0.208 0 iCAF CXCL14

# 3 0 3.033794 0.703 0.308 0 iCAF CFD

# 4 0 2.613782 0.348 0.041 0 iCAF PI16

# 5 0 2.173342 0.609 0.283 0 iCAF IGFBP6

# 6 0 2.129693 0.451 0.084 0 iCAF TNXB

library('ggplot2')

library('dplyr')

library('ggrepel')

library('ggpubr')

#

df=all.markers

#

head(df)

# p_val avg_log2FC pct.1 pct.2 p_val_adj cluster gene

# PLA2G2A 0 3.413885 0.312 0.023 0 iCAF PLA2G2A

# CXCL14 0 3.086529 0.576 0.208 0 iCAF CXCL14

# CFD 0 3.033794 0.703 0.308 0 iCAF CFD

# PI16 0 2.613782 0.348 0.041 0 iCAF PI16

# IGFBP6 0 2.173342 0.609 0.283 0 iCAF IGFBP6

# TNXB 0 2.129693 0.451 0.084 0 iCAF TNXB

#添加显著性标签：

df$label <- ifelse(df$p_val_adj<0.05,"adjust P-val<0.05","adjust P-val>=0.05")

head(df)

#

#获取每个cluster中表达差异最显著的10个基因；

table(df$cluster)

top10sigiCAF <- dplyr::filter(df,cluster=="iCAF") %>% distinct(gene,.keep_all = T) %>% top_n(10,abs(avg_log2FC))

head(top10sigiCAF)

#

top10sigmyCAF <- dplyr::filter(df,cluster=="myCAF") %>% distinct(gene,.keep_all = T) %>% top_n(10,abs(avg_log2FC))

head(top10sigmyCAF)

#

top10sigEMT_like <- dplyr::filter(df,cluster=="EMT_like CAF") %>% distinct(gene,.keep_all = T) %>% top_n(10,abs(avg_log2FC))

head(top10sigEMT_like)

#

top10sigVSMC <- dplyr::filter(df,cluster=="VSMC") %>% distinct(gene,.keep_all = T) %>% top_n(10,abs(avg_log2FC))

head(top10sigVSMC)

#

top10sigUndefined_fib <- dplyr::filter(df,cluster=="Undefined_fib") %>% distinct(gene,.keep_all = T) %>% top_n(10,abs(avg_log2FC))

head(top10sigUndefined_fib)

#

top10sigapCAF <- dplyr::filter(df,cluster=="apCAF") %>% distinct(gene,.keep_all = T) %>% top_n(10,abs(avg_log2FC))

head(top10sigapCAF)

#

top10sigUndefined_CAF <- dplyr::filter(df,cluster=="Undefined_CAF") %>% distinct(gene,.keep_all = T) %>% top_n(10,abs(avg_log2FC))

head(top10sigUndefined_CAF)

#

top10sigPericyte <- dplyr::filter(df,cluster=="Pericyte") %>% distinct(gene,.keep_all = T) %>% top_n(10,abs(avg_log2FC))

head(top10sigPericyte)

#

top10sigNAF <- dplyr::filter(df,cluster=="NAF") %>% distinct(gene,.keep_all = T) %>% top_n(10,abs(avg_log2FC))

head(top10sigNAF)

#

#将提取所有cluster的Top10基因表格合并：

top10sig <- rbind(top10sigiCAF,top10sigmyCAF,top10sigEMT_like,top10sigVSMC,top10sigUndefined_fib,

top10sigapCAF,top10sigUndefined_CAF,top10sigPericyte,top10sigNAF)

top10sig <- rbind(top10sigmyCAF,top10sigVSMC,top10sigPericyte)

# 筛选df

df <- df %>%

dplyr::filter(cluster %in% c("myCAF", "VSMC", "Pericyte"))

#新增一列，将Top10的差异基因标记为2，其他的标记为1；

df$size <- case_when(!(df$gene %in% top10sig$gene)~ 1,

df$gene %in% top10sig$gene ~ 2)

#提取非Top10的基因表格；

dt <- dplyr::filter(df,size==1)

head(dt)

#

# 然后是绘图第一步！分别绘制需要带geneID标签和不需要带标签的两个火山图并叠加起来。

#绘制每个Cluster Top10以外基因的散点火山图：

p <- ggplot()+

geom_jitter(data = dt,

aes(x = cluster, y = avg_log2FC, color = label),

size = 0.85,

width =0.4)

p

#叠加每个Cluster Top10基因散点(将散点适当放大强调）：

p <- ggplot()+

geom_jitter(data = dt,

aes(x = cluster, y = avg_log2FC, color = label),

size = 0.85,

width =0.4)+

geom_jitter(data = top10sig,

aes(x = cluster, y = avg_log2FC, color = label),

size = 1,

width =0.4)

p

# 接着开始第二步，画背景的灰色柱子，并把散点叠上去。

#根据图p中log2FC区间确定背景柱长度：

dfbar<-data.frame(x=c("iCAF","myCAF","EMT_like CAF","VSMC","Undefined_fib","apCAF","Undefined_CAF","Pericyte","NAF"),

y=c(4,4,4,4,1,5,5,5,4.5))

dfbar<-data.frame(x=c("myCAF","VSMC","Pericyte"),

y=c(4,4,5))

# dfbar1<-data.frame(x=c(0,1,2,3,4,5,6,7,8),

# y=c(-1.05,-1.1,-1.3,-1.3,-1.8,-1.55,-1.3,-1.9,-0.85))

#绘制背景柱：

p1 <- ggplot()+

geom_col(data = dfbar,

mapping = aes(x = x,y = y),

fill = "#dcdcdc",alpha = 0.6)

# +

# geom_col(data = dfbar1,

# mapping = aes(x = x,y = y),

# fill = "#dcdcdc",alpha = 0.6)

p1

#把散点火山图叠加到背景柱上：

pal_npg("nrc",alpha = 0.9)(10)

# [1] "#E64B35E5" "#4DBBD5E5" "#00A087E5" "#3C5488E5" "#F39B7FE5" "#8491B4E5" "#91D1C2E5"

# [8] "#DC0000E5" "#7E6148E5" "#B09C85E5"

p2 <- ggplot()+

geom_col(data = dfbar,

mapping = aes(x = x,y = y),

fill = "#dcdcdc",alpha = 0.6)+

# geom_col(data = dfbar1,

# mapping = aes(x = x,y = y),

# fill = "#dcdcdc",alpha = 0.6)+

geom_jitter(data = dt,

aes(x = cluster, y = avg_log2FC, color = label),

size = 0.85,

width =0.4)+

geom_jitter(data = top10sig,

aes(x = cluster, y = avg_log2FC, color = label),

size = 1,

width =0.4)+

scale_color_manual(values = c("adjust P-val<0.05" = "#E64B35E5", "adjust P-val>=0.05" = "#4DBBD5E5"))

# scale_color_brewer(palette = "Set2")

# scale_color_viridis_d(option = "plasma") # 选择一种 `viridis` 渐变配色

p2

# 最后是第三步！绘制cluster色块并进行叠加：

#添加X轴的cluster色块标签：

# dfcol<-data.frame(x=c(1:9),

# y=0,

# label=c(0:8))

# mycol <- c("#E64B357F","#4DBBD57F","#00A0877F","#3C54887F","#F39B7F7F","#8491B47F","#91D1C27F","#DC00007F","#7E61487F")

#

my36colors <-c('#E5D2DD', '#53A85F', '#F1BB72', '#F3B1A0', '#D6E7A3', '#57C3F3', '#476D87',

'#E95C59', '#E59CC4', '#AB3282', '#23452F', '#BD956A', '#8C549C', '#585658',

'#9FA3A8', '#E0D4CA', '#5F3D69', '#C5DEBA', '#58A4C3', '#E4C755', '#F7F398',

'#AA9A59', '#E63863', '#E39A35', '#C1E6F3', '#6778AE', '#91D0BE', '#B53E2B',

'#712820', '#DCC1DD', '#CCE0F5', '#CCC9E6', '#625D9E', '#68A180', '#3A6963',

'#968175'

)

dfcol<-data.frame(x=c(1:3),

y=0,

label=c(0:2))

mycol <- c("#53A85F","#F3B1A0","#E95C59")

p3 <- p2 + geom_tile(data = dfcol,

aes(x=x,y=y),

height=0.4,

color = "black",

fill = mycol,

alpha = 1, # 调方块颜色深度

show.legend = F)

p3

# 现在这张火山图就算初步画完，最后剩下一些geneID标签的添加、主题美化等常规操作。

#给每个Cluster差异表达前Top10基因加上标签：

p4 <- p3+

geom_text_repel(

data=top10sig,

aes(x=cluster,y=avg_log2FC,label=gene),

force = 1.2,

arrow = arrow(length = unit(0.008, "npc"),

type = "open", ends = "last")

)

p4

# 富集

load("~/my_prepare/lfr_finally/Breastcancer_EMBOJ/emboj_预后模型/wgcna/new/inputgene_24_7_19.Rdata")

# 输入文件

all.markers = markers_genes %>% dplyr::filter(p_val<0.05)

#

# top5= all.markers %>% group_by(cluster) %>% top_n(n = 5, wt = avg_log2FC)

top10= all.markers %>% group_by(cluster) %>% top_n(n = 10, wt = avg_log2FC)

top10=as.data.frame(top10)

head(top10)

# 筛选下，只要三群

# all.markers_MVP=all.markers %>% filter(cluster==c("myCAF","VSMC","Pericyte"))

table(all.markers$cluster)

# iCAF myCAF EMT_like CAF VSMC Undefined_fib apCAF

# 361 291 464 289 116 402

# Undefined_CAF Pericyte NAF

# 310 188 1099

top150= all.markers %>% group_by(cluster) %>% top_n(n = 50, wt = avg_log2FC)

table(top150$cluster)

all.markers=top150

VSMC=all.markers[all.markers$cluster=='VSMC',]$gene

Pericyte=all.markers[all.markers$cluster=='Pericyte',]$gene

myCAFs=all.markers[all.markers$cluster=='myCAF',]$gene

# total <- list(VSMC=VSMC,Pericyte=Pericyte,myCAFs=myCAFs)

# 多分组富集分析

all.markers_MVP=all.markers %>% dplyr::filter(cluster %in% c("myCAF","VSMC","Pericyte"))

#

library(clusterProfiler)

library(ggplot2)

# 构建分组，转化genesymbol-gene ID。

df_sig=all.markers_MVP

group <- data.frame(gene=df_sig$gene,

group=df_sig$cluster)

Gene_ID <- bitr(df_sig$gene, fromType="SYMBOL",

toType="ENTREZID",

OrgDb="org.Hs.eg.db")

#构建文件并分析

data <- merge(Gene_ID,group,by.x='SYMBOL',by.y='gene')

# 富集分析，去除冗杂terms！

data_GO <- compareCluster(

ENTREZID~group,

data=data,

fun="enrichGO",

OrgDb="org.Hs.eg.db",

ont = "BP",

pAdjustMethod = "BH",

pvalueCutoff = 0.05,

qvalueCutoff = 0.05

)

head(data_GO)

dotplot(data_GO, showCategory=10,font.size = 8)

data_Go_fil=data_GO@compareClusterResult

data_GO_sim <- simplify(data_GO, # 使用 simplify() 函数来简化基因本体（GO）数据，以减少冗余的 GO 术语【相似性大于0.7的term，被认为是冗余的】。

cutoff=0.7,

by="p.adjust",

select_fun=min)

dotplot(data_GO_sim, showCategory=10,font.size = 12)

data_GO_sim_fil <- data_GO_sim@compareClusterResult # 保存条目

save(data_GO_sim_fil,file = "../出图_24_9_3/fig3b三群细胞高表达基因富集.Rdata")

# 导出表格

load("~/my_prepare/lfr_finally/Breastcancer_EMBOJ/emboj_预后模型/出图_24_9_3/fig3b三群细胞高表达基因富集.Rdata")

write.csv(data_GO_sim_fil,file = "../出图_24_9_3/附表8_三群细胞特征基因富集结果.csv")

# 到ggplot进行可视化编辑

# 每个分组按照p.dajust排序，取top10

top10_per_cluster <- data_GO_sim_fil %>%

group_by(Cluster) %>% # 按 Cluster 分组

slice_min(order_by = p.adjust, n = 10) # 选取每组 p.adjust 最小的前 10 项

#

df_GO=top10_per_cluster

df_GO$Description <- str_wrap(df_GO$Description, width = 45) # 超过指定长度，自动换行

library(forcats)

df_GO$Description <- as.factor(df_GO$Description)

df_GO$Description <- fct_inorder(df_GO$Description)

#

ggplot(df_GO, aes(Cluster, Description)) +

geom_point(aes(fill=p.adjust, size=Count), shape=21)+

theme_bw()+

theme(axis.text.x=element_text(angle=90,hjust = 1,vjust=0.5),

axis.text = element_text(color = 'black', size = 10))+

scale_fill_gradient(low="red",high="#4DBBD5E5")+

labs(x=NULL,y=NULL)

ggplot(df_GO, aes(Cluster, Description)) +

geom_point(aes(color=p.adjust, size=Count), shape=16)+

theme_bw()+

theme(axis.text.x=element_text(angle=90,hjust = 1,vjust=0.5),

axis.text = element_text(color = 'black', size = 12))+

scale_color_gradient(low="red",high="blue")+

labs(x=NULL,y=NULL)

# +

# coord_flip() # 将图形的 x 轴和 y 轴进行翻转

# wgcna

load("~/my_prepare/lfr_finally/Breastcancer_EMBOJ/emboj_预后模型/101机器学习/Mime1包/mydata/GSE58812_清洗好的data.Rdata")

load("~/my_prepare/lfr_finally/Breastcancer_EMBOJ/emboj_预后模型/wgcna/new/分型信息.Rdata")

# load("~/my_prepare/lfr_finally/Breastcancer_EMBOJ/emboj_预后模型/wgcna/new/inputgene_24_7_19.Rdata")

exp_filter=exp

exp_filter[1:4,1:4]

m.mad <- apply(exp_filter,1,mad)

dataExprVar <- exp_filter[which(m.mad >

max(quantile(m.mad, probs=seq(0, 1, 0.25))[2],0.01)),]

data.mat=t(dataExprVar)

dim(data.mat) # [1] 107 15141

# 要更改为行为样本，列为基因

# 然后只保留MP相关基因

data.mat=data.mat[,colnames(data.mat)%in%geneset1$gene]

dim(data.mat) # [1] 107 319

# 下面过滤异常样本

library(WGCNA)

gsg <- goodSamplesGenes(data.mat, verbose = 3)

gsg$allOK

# TRUE

# 如果返回为true,证明没有缺失值，可以直接进行下一步

# 如果为false,则需要用以下代码进行删除缺失值

# 如果存在异常样本或基因

if (!gsg$allOK) {

# 异常的基因

if (sum(!gsg$goodGenes)>0)

printFlush(paste("Removing genes:",

paste(names(data.mat)[!gsg$goodGenes], collapse = ",")));

# 异常的样本

if (sum(!gsg$goodSamples)>0)

printFlush(paste("Removing samples:",

paste(rownames(data.mat)[!gsg$goodSamples], collapse = ",")));

# 删除异常的样本和基因

data.mat = data.mat[gsg$goodSamples, gsg$goodGenes]

}

# 下一步：聚类所有样本，观察是否有离群样本或者异常样本

# 删除剪切线以下的样本，查看图片，选取离群值

# 如果不想删除可以将cutHeight设置高些

# 绘制样本聚类图

sampleTree <- hclust(dist(data.mat), method = "average")

plot(sampleTree, main = "Sample clustering to detect outliers", sub="", xlab="")

# 根据上图，设置一个剪切线，将离群样本删除

# Plot a line to show the cut

abline(h = 25, col = "red");

# Determine cluster under the line

clust = cutreeStatic(sampleTree, cutHeight = 25, minSize = 10)

table(clust)

# 赋值，省的后面改代码

datExpr0=data.mat

outSamples <- datExpr0[(clust==0),]

rownames(outSamples)

# clust 1 contains the samples we want to keep.

keepSamples <- (clust==1)

datExpr <- datExpr0[keepSamples, ]

sampleTree2 <- hclust(dist(datExpr), method = "average");

par(cex = 0.5);

par(mar = c(0,6,2,0))

plot(sampleTree2, main = "Sample clustering to detect outliers", sub="", xlab="", cex.lab = 1.5,

cex.axis = 1.5, cex.main = 2)

# 加入临床特征，前面准备好了

# 有一点需要注意，这里能作为输入的必须为数值型特征。

# 把high改为1，low改为0

head(a)

# a=a[,-1]

# MP_score MP_group

# GSM1419942 2.548510 high

# GSM1419943 2.216109 low

# GSM1419944 2.081702 low

# GSM1419945 2.649311 high

# GSM1419946 2.475124 high

# GSM1419947 2.511488 high

a$group=NA

a$group[which(str_detect(a$MP_group, "^high"))] <- "1"

a$group[which(str_detect(a$MP_group, "^low"))] <- "0"

table(a$group)

# 0 1

# 54 53

datTraits=a

datTraits[,3]=as.numeric(datTraits[,3])

head(datTraits)

# MP_score MP_group group

# GSM1419942 2.548510 high 1

# GSM1419943 2.216109 low 0

# GSM1419944 2.081702 low 0

# GSM1419945 2.649311 high 1

# GSM1419946 2.475124 high 1

# GSM1419947 2.511488 high 1

#

identical(rownames(datExpr),rownames(datTraits))

#

datTraits=datTraits[rownames(datTraits)%in%rownames(datExpr),]

identical(rownames(datExpr),rownames(datTraits))

# [1] FALSE

# datExpr=datExpr[rownames(datExpr)%in%rownames(datTraits),]

# identical(rownames(datExpr),rownames(datTraits))

# # [1] TRUE

datTraits=datTraits[3]

rownames(datTraits)

# collectGarbage()

# 增加性状信息后，再次聚类

sampleTree2=hclust(dist(datExpr),method = "average")

# 绘图

# Convert traits to a color representation: white means low, red means high, grey means missing entry

traitColors <- numbers2colors(datTraits, signed = FALSE);

# Plot the sample dendrogram and the colors underneath.

plotDendroAndColors(sampleTree2, traitColors,

groupLabels = names(datTraits),

main = "Sample dendrogram and trait heatmap")

save(datExpr0,datExpr, datTraits, file = "../../Breastcancer_EMBOJ/emboj_预后模型/wgcna/new/GEO-01-dataInput.RData")

# 2、网路构建和模块检测

# 2.1 选择软阈值

# 使用函数**pickSoftThreshold()**选择适当的软阈值。

rm(list = ls())

options(stringsAsFactors = F)

library(WGCNA)

load("~/my_prepare/lfr_finally/Breastcancer_EMBOJ/emboj_预后模型/wgcna/new/GEO-01-dataInput.RData")

# Choose a set of soft-thresholding powers

powers <- c(c(1:10), seq(from = 12, to=20, by=2))

# Call the network topology analysis function

sft <- pickSoftThreshold(datExpr, powerVector = powers, verbose = 5)

# Plot the results:

par(mar = c(3,6,2,1))

par(mfrow = c(1,2))

cex1 = 0.9 #可以修改

# 拟合指数与power值散点图，无标度拓扑拟合指数

# 一般选择在0.9以上的，第一个达到0.9以上的数值

# Scale-free topology fit index as a function of the soft-thresholding power

plot(sft$fitIndices[,1], -sign(sft$fitIndices[,3])*sft$fitIndices[,2],

xlab="Soft Threshold (power)",

ylab="Scale Free Topology Model Fit,signed R^2",

type="n", # n表示不绘图

main = paste("Scale independence"))

text(sft$fitIndices[,1], -sign(sft$fitIndices[,3])*sft$fitIndices[,2],

labels=powers, cex=cex1, col="red")

# best

sft$powerEstimate

# this line corresponds to using an R^2 cut-off of h

abline(h=0.9,col="red") # 这边h设置为cex1的值

# 越平滑越好

# 平均连通性与power值散点图

# Mean connectivity as a function of the soft-thresholding power

plot(sft$fitIndices[,1], sft$fitIndices[,5],

xlab="Soft Threshold (power)",

ylab="Mean Connectivity",

type="n",

main = paste("Mean connectivity"))

text(sft$fitIndices[,1], sft$fitIndices[,5], labels=powers, cex=cex1,col="red")

abline(h=10,col="red")

# 其中sft$powerEstimate是推荐的最优软阈值，最优值为4。左图y轴是无标度拓扑拟合指数，右图y轴是平均连通度。

# 其中横坐标为软阈值的梯度，第一幅图的纵坐标为无标度网络适应系数，越大越好；第二幅图的纵坐标为节点的平均连通度，越小越好。

sft$powerEstimate

## 如果推荐的最优软阈值为NA，则表面系统无法给出合适的软阈值，这时候就需要自己挑选软阈值。

sft

softpower=sft$powerEstimate

adjacency=adjacency(datExpr,power = softpower)

softpower

# TOM矩阵

TOM=TOMsimilarity(adjacency)

dissTOM=1-TOM

# 基因聚类

geneTree=hclust(as.dist(dissTOM),method = "average")

plot(geneTree,xlab="",sub="",main="Gene clustering on TOM-based dissimilarity",

labels=FALSE,hang=0.04)

# 动态剪切模块识别

minModuleSize=20 # 最小单个模块包含的基因数

dynamicMods=cutreeDynamic(dendro = geneTree,distM = dissTOM,

deepSplit = 2,pamRespectsDendro = FALSE,

minClusterSize = minModuleSize)

table(dynamicMods)

dynamicColors=labels2colors(dynamicMods)

table(dynamicColors)

plotDendroAndColors(geneTree,dynamicColors,"Dynamic Tree Cut",

dendroLabels = FALSE,hang=0.1,

addGuide = TRUE,guideHang = 0.1,

main="Gene dendrogram and module colors")

net <- blockwiseModules(datExpr, power = 3,

maxBlockSize = 2000,# 最大模块数量

TOMType = "unsigned",

minModuleSize = 20, #用于模块检测的最小模块尺

reassignThreshold = 0,

mergeCutHeight = 0.25, # 用于模块合并的树形图切割高度

numericLabels = TRUE, pamRespectsDendro = FALSE,

saveTOMs = TRUE,

saveTOMFileBase = "../../../Breastcancer_EMBOJ/emboj_预后模型/wgcna/new/TNBCTOM",

verbose = 3)

# minModuleSize表示用于模块检测的最小模块尺寸。mergeCutHeight表示用于模块合并的树形图切割高度。这两个值越大，模块越少。saveTOMFileBase用来设置数据保存位置及名称。

table(net$colors)

# 0 1

# 91 228

# 查看划分的模块数和每个模块里面包含的基因个数。0代表无法识别的基因数。

# 用于模块识别的分层聚类树形图存储在net$dendprograms[[1]]中。可以与模型颜色分配一起显示。

sizeGrWindow(12, 9)

# Convert labels to colors for plotting

mergedColors <- labels2colors(net$colors)

# Plot the dendrogram and the module colors underneath

plotDendroAndColors(net$dendrograms[[1]], mergedColors[net$blockGenes[[1]]],

"Module colors",

dendroLabels = FALSE, hang = 0.1,

addGuide = TRUE, guideHang = 0.04)

# 除此之外，还有分布网络构建和模块检测，以及处理大数据集的：分块网络构建和模块检测。

# 保存参数

moduleLabels <- net$colors

moduleColors <- labels2colors(net$colors)

MEs <- net$MEs;

geneTree <- net$dendrograms[[1]];

save(MEs, moduleLabels, moduleColors, geneTree,

file = "../../Breastcancer_EMBOJ/emboj_预后模型/wgcna/new/GEO-02-networkConstruction-auto.RData")

# 3、将模块与表型数据关联并识别重要基因

# 3.1 模块与性状关系图

rm(list = ls())

options(stringsAsFactors = F)

library(WGCNA)

load("~/my_prepare/lfr_finally/Breastcancer_EMBOJ/emboj_预后模型/wgcna/new/GEO-01-dataInput.RData")

load("~/my_prepare/lfr_finally/Breastcancer_EMBOJ/emboj_预后模型/wgcna/new/GEO-02-networkConstruction-auto.RData")

# Define numbers of genes and samples

nGenes <- ncol(datExpr)

nSamples <- nrow(datExpr)

# Recalculate MEs with color labels

MEs0 <- moduleEigengenes(datExpr, moduleColors)$eigengenes

MEs <- orderMEs(MEs0)

moduleTraitCor <- cor(MEs, datTraits, use = "p");

moduleTraitPvalue <- corPvalueStudent(moduleTraitCor, nSamples)

sizeGrWindow(10,6)

# Will display correlations and their p-values

textMatrix <- paste(signif(moduleTraitCor, 2), "\n(",

signif(moduleTraitPvalue, 1), ")", sep = "")

dim(textMatrix) <- dim(moduleTraitCor)

par(mar = c(6, 10, 3, 3))

# Display the correlation values within a heatmap plot

labeledHeatmap(Matrix = moduleTraitCor,

xLabels = names(datTraits),

yLabels = names(MEs),

ySymbols = names(MEs),

colorLabels = FALSE,

colors = blueWhiteRed(50),

textMatrix = textMatrix,

setStdMargins = FALSE,

cex.text = 0.5,

cex.lab = 0.8,

zlim = c(-1,1),

main = paste("Module-trait relationships"))

# 图中展示了3个模块与1种性状的关系，其中红色代表模块与性状正相关，蓝色则代表负相关。

# 我们要选择与性状最相关的模块，那么就是MEturquoise对应的模块，其P值为0.65。

# 3、青色模块与MP评分的关系（聚类图和热图）

# 使用plotEigengeneNetworks函数再次验证青色模块与肿瘤是否有关联。

# Recalculate module eigengenes

MEs <- moduleEigengenes(datExpr, moduleColors)$eigengenes

# 注意：记得修改代码，选择你感兴趣的性状

samples <- as.data.frame(datTraits$group);

names(samples) <- "samples"

# Add the weight to existing module eigengenes

MET <- orderMEs(cbind(MEs, samples))

# Plot the relationships among the eigengenes and the trait

sizeGrWindow(5,9)

par(cex = 0.9)

plotEigengeneNetworks(MET, "", marDendro = c(0,4,1,2), marHeatmap = c(4,5,1,2),

cex.lab = 0.8, xLabelsAngle = 90)

# 聚类图中可以看出samples与MEturquiose高度相关，在热图中青色模块与samples也有显著相关性。说明青色模块就是与肿瘤最相关的模块。

# 也可以将聚类图和热图分开展示。

# Plot the dendrogram

sizeGrWindow(6,6)

par(cex = 1.0)

plotEigengeneNetworks(MET, "Eigengene dendrogram", marDendro = c(0,4,2,0),

plotHeatmaps = FALSE)

# Plot the heatmap matrix (note: this plot will overwrite the dendrogram plot)

par(cex = 1.0)

plotEigengeneNetworks(MET, "Eigengene adjacency heatmap", marHeatmap = c(3,4,2,2),

plotDendrograms = FALSE, xLabelsAngle = 90)

# 4、青色模块中MP的GS与MM的相关性（散点图）

# 我们将基因显著性 (Gene Significance, GS) 定义为基因与性状之间相关性的（绝对值），以此量化单个基因与我们感兴趣的性状（权重）之间的关联。

# 对于每个模块，我们还定义了一个定量指标模块成员(module membership, MM），即模块特征基因与基因表达谱的相关性。这样我们就可以量化阵列上所有基因与每个模块的相似性。代码实现如下：

samples <- as.data.frame(datTraits$group);

names(samples) <- "samples"

# names (colors) of the modules

modNames <- substring(names(MEs), 3)

geneModuleMembership <- as.data.frame(cor(datExpr, MEs, use = "p"));

MMPvalue <- as.data.frame(corPvalueStudent(as.matrix(geneModuleMembership), nSamples))

names(geneModuleMembership) <- paste("MM", modNames, sep="")

names(MMPvalue) <- paste("p.MM", modNames, sep="")

geneTraitSignificance <- as.data.frame(cor(datExpr, samples, use = "p"))

GSPvalue <- as.data.frame(corPvalueStudent(as.matrix(geneTraitSignificance), nSamples))

names(geneTraitSignificance) <- paste("GS.", names(samples), sep="")

names(GSPvalue) <- paste("p.GS.", names(samples), sep="")

# 注意：其中前两行用来选择你感兴趣的性状，记得修改成对应的数据。

# 针对感兴趣的模块，将GS与MM值可视化，代码如下：

modNames

# [1] "blue" "grey" "turquoise"

module <- "blue"

column <- match(module, modNames);

moduleGenes <- moduleColors==module;

sizeGrWindow(7, 7);

par(mfrow = c(1,1));

# [1] "#E64B35E5" "#4DBBD5E5" "#00A087E5" "#3C5488E5" "#F39B7FE5" "#8491B4E5" "#91D1C2E5"

# [8] "#DC0000E5" "#7E6148E5" "#B09C85E5"

verboseScatterplot(abs(geneModuleMembership[moduleGenes, column]),

abs(geneTraitSignificance[moduleGenes, 1]),

xlab = paste("Module Membership in", module, "module"),

ylab = "Gene significance for sample type",

main = paste("Module membership vs. gene significance\n"),

cex.main = 1.2, cex.lab = 1.2, cex.axis = 1.2, col = "#029AE5E5")

verboseScatterplot(abs(geneModuleMembership[moduleGenes, column]),

abs(geneTraitSignificance[moduleGenes, 1]),

xlab = paste("Module Membership in", module, "module"),

ylab = "Gene significance for sample type",

main = paste("Module membership vs. gene significance\n"),

cex.main = 1.2, cex.lab = 1.2, cex.axis = 1.2, col = "turquoise")

# 注意：module对应的是你感兴趣模块的颜色，记得修改图中的文字。

# 再注意：verboseScatterplot函数中的col本来是“col = module”，但是由于黄色太不明显了，我就用另一种黄色代替了。

# 图中的每一个点代表一个基因。显然，图中GS 和 MM 高度相关，说明与肿瘤高度显著相关的基因往往也是黄色模块中最重要（核心）的元素。

#

# 5、保存与肿瘤最相关的基因

colnames(datExpr)

table(moduleGenes)

sel_genes1 <- colnames(datExpr)[moduleColors=="turquoise"]

sel_genes2 <- colnames(datExpr)[moduleColors=="blue"]

# write.table(sel_genes, file = "../../../../Breastcancer_EMBOJ/emboj_预后模型/wgcna/module_gene_name.txt", quote = F,

# row.names = F,col.names = F)

sel_genes=c(sel_genes1,sel_genes2)

save(sel_genes,sel_genes1,sel_genes2,file = "../../Breastcancer_EMBOJ/emboj_预后模型/wgcna/new/最相关模块基因.Rdata")

# 单因素cox

load("~/my_prepare/lfr_finally/Breastcancer_EMBOJ/emboj_预后模型/wgcna/new/最相关模块基因.Rdata")

# 单因素回归

# 通过一个for循环对所有目标基因进行回归分析，并且以dataframe的形式对结果进行输出：

load("~/my_prepare/lfr_finally/Breastcancer_EMBOJ/emboj_预后模型/101机器学习/Mime1包/mydata/GSE58812_不分割log的清洗好的data.Rdata")

Train[1:4,1:4]

sigGenes=c("OS.time","OS")

hub_gene=c(sel_genes,sigGenes)

Train=Train[,colnames(Train)%in%hub_gene]

data=Train

data$OS.time=as.numeric(data$OS.time)

data$OS=as.numeric(data$OS)

result=data.frame()

for(i in colnames(data[,3:ncol(data)])){

cox<- coxph(Surv(OS.time, OS) ~ get(i), data = data)

coxSummary = summary(cox)

result=rbind(result,

cbind(id=i,

HR=coxSummary$conf.int[,"exp(coef)"],

HR.95L=coxSummary$conf.int[,"lower .95"],

HR.95H=coxSummary$conf.int[,"upper .95"],

pvalue=coxSummary$coefficients[,"Pr(>|z|)"]))

}

result[,2:5] <-apply(result[,2:5],2,as.numeric)

# 通过P值以及HR对有预后意义的基因进行筛选

table(result$pvalue<0.05)

# 森林图绘制：#读取输入文件

head(outTab)

data=outTab

# 将第一列变成行名：

rownames(data) <- data$id

data <- data[,-1]

head(data)

data$HR=as.numeric(data$HR)

data$HR.95L=as.numeric(data$HR.95L)

data$HR.95H=as.numeric(data$HR.95H)

data$pvalue=as.numeric(data$pvalue)

# 列名不能自行修改，如果完全不懂代码的小白，最好完全照着我的数据，保持列名与我的一致，不然容易报错。

rt <- data

hr <- sprintf("%.3f",rt$"HR")

hrLow <- sprintf("%.3f",rt$"HR.95L")

hrHigh <- sprintf("%.3f",rt$"HR.95H")

Hazard.ratio <- paste0(hr,"(",hrLow,"-",hrHigh,")")

pVal <- ifelse(rt$pvalue<0.001, "<0.001", sprintf("%.3f", rt$pvalue))

gene <- rownames(rt)

n <- nrow(rt)

nRow <- n+1

ylim <- c(1,nRow)

layout(matrix(c(1,2),nc=2),width=c(3,2.5))

xlim = c(0,3)

par(mar=c(4,2.5,2,1))

plot(1,xlim=xlim,ylim=ylim,type="n",axes=F,xlab="",ylab="")

text.cex=0.8

text(0,n:1,gene,adj=0,cex=text.cex)

text(1.5-0.5*0.2,n:1,pVal,adj=1,cex=text.cex);text(1.5-0.5*0.2,n+1,'pvalue',cex=text.cex,font=2,adj=1)

text(3,n:1,Hazard.ratio,adj=1,cex=text.cex);text(3,n+1,'Hazard ratio',cex=text.cex,font=2,adj=1,)

par(mar=c(4,1,2,1),mgp=c(2,0.5,0))

xlim = c(0,max(as.numeric(hrLow),as.numeric(hrHigh)))

plot(1,xlim=xlim,ylim=ylim,type="n",axes=F,ylab="",xaxs="i",xlab="Hazard ratio")

arrows(as.numeric(hrLow),n:1,as.numeric(hrHigh),n:1,angle=90,code=3,length=0.05,col="darkblue",lwd=2.5)

abline(v=1,col="black",lty=2,lwd=2)

boxcolor = ifelse((as.numeric(hr)>1)&(pVal<0.05),'red3',"green3")

points(as.numeric(hr), n:1, pch = 15, col = boxcolor, cex=1.3)

axis(1)

# 设置表格内容

tabletext <- cbind(

c("Gene", outTab$id),

c("HR", sprintf("%.3f", outTab$HR)),

c("95% CI", paste(sprintf("%.3f", outTab$HR.95L), "-", sprintf("%.3f", outTab$HR.95H))),

c("P value", sprintf("%.3e", outTab$pvalue))

)

forestplot(

tabletext,

mean = c(NA, outTab$HR),

lower = c(NA, outTab$HR.95L),

upper = c(NA, outTab$HR.95H),

zero = 1, # 指定零点线

boxsize = 0.3, # 方块大小

lineheight = unit(1.5, "cm"), # 每一行的高度

col = fpColors(box = color_palette, lines = "#6666FF", zero = "gray50"), # 设置颜色：显著性HR点用 `color_palette` 动态分配，线条为深蓝色，零点线为灰色

xlog = FALSE, # 对数尺度展示

ci.vertices = TRUE, # 显示置信区间顶点

ci.vertices.height = 0.1, # 置信区间顶点高度

title = "Enhanced Forest Plot of HR with 95% CI",

graphwidth = unit(8, "cm"), # 增加图形宽度

xticks = c(0,0.5, 1, 2, 4, 8,10,12), # 自定义X轴刻度

txt_gp = fpTxtGp(

label = gpar(fontsize = 12, fontface = "bold"), # 基因名称加粗

ticks = gpar(fontsize = 10), # 刻度字体大小

xlab = gpar(fontsize = 12) # X轴标签字体

),

hrzl_lines = gpar(col = "#444444"), # 水平线颜色

grid = gpar(lty = 2, col = "#CCCCCC"), # 添加背景网格线

clip = c(0, 10), # 设置置信区间的上下限

is.summary = FALSE, # 取消汇总线

new_page = TRUE # 在新页面上绘制

)

forestplot(

tabletext, # 包含文本的矩阵，用于显示森林图左侧的基因信息、HR值、95% CI 和 p 值等

mean = c(NA, outTab$HR), # 中心点，显示HR值，第一项NA是为了对齐表头

lower = c(NA, outTab$HR.95L), # 置信区间的下限值

upper = c(NA, outTab$HR.95H), # 置信区间的上限值

zero = 1, # 指定零点线为1（HR=1是分界点，表示无效假设）

boxsize = 0.3, # 设置方块大小，表示HR的可视化点

lineheight = unit(1.5, "cm"), # 每一行的高度，确保表格和点之间有足够的间距

col = fpColors(box = color_palette, lines = "darkblue", zero = "gray50"), # 设置颜色：显著性HR点用 `color_palette` 动态分配，线条为深蓝色，零点线为灰色

xlog = FALSE, # 设置X轴为线性尺度而不是对数尺度

ci.vertices = TRUE, # 在置信区间末端显示顶点

ci.vertices.height = 0.1, # 设置置信区间顶点的高度

title = "Enhanced Forest Plot of HR with 95% CI", # 设置森林图的标题

graphwidth = unit(8, "cm"), # 调整图形的宽度为8厘米

xticks = c(0,0.5, 1, 2, 4, 8,10,11), # 设置X轴刻度范围，范围从0到5，步长为0.5

txt_gp = fpTxtGp(

label = gpar(fontsize = 12, fontface = "bold"), # 基因名称字体大小为12，并加粗

ticks = gpar(fontsize = 10), # X轴刻度字体大小

xlab = gpar(fontsize = 12) # X轴标签字体大小

),

hrzl_lines = gpar(col = "#444444"), # 水平线的颜色设置为灰色

grid = gpar(lty = 2, col = "#CCCCCC"), # 添加背景网格线，颜色为浅灰色，虚线样式

clip = c(0, 5), # 限制置信区间的显示范围，最小为0，最大为5

is.summary = FALSE, # 指定该图不是汇总图

new_page = TRUE # 在新页面上开始绘图

)

#

Fig5.R

# 模型

load("~/my_prepare/lfr_finally/Breastcancer_EMBOJ/emboj_预后模型/101机器学习/Mime1包/mydata/GSE58812_data.Rdata")

Train$OS.time=as.numeric(Train$OS.time)

Train=Train %>% filter(OS.time>200)

#

load("~/my_prepare/lfr_finally/Breastcancer_EMBOJ/emboj_预后模型/101机器学习/自己代码跑/traindataset.Rdata")

TrainDataset$OS.time=as.numeric(TrainDataset$OS.time)

TrainDataset=TrainDataset %>% filter(OS.time>200)

sigGenes=c("OS.time","OS")

hub_gene=c(sel_genes,sigGenes)

Train=Train[,colnames(Train)%in%hub_gene]

data=Train

data$OS.time=as.numeric(data$OS.time)

data$OS=as.numeric(data$OS)

result=data.frame()

for(i in colnames(data[,3:ncol(data)])){

cox<- coxph(Surv(OS.time, OS) ~ get(i), data = data)

coxSummary = summary(cox)

result=rbind(result,

cbind(id=i,

HR=coxSummary$conf.int[,"exp(coef)"],

HR.95L=coxSummary$conf.int[,"lower .95"],

HR.95H=coxSummary$conf.int[,"upper .95"],

pvalue=coxSummary$coefficients[,"Pr(>|z|)"]))

}

result[,2:5] <-apply(result[,2:5],2,as.numeric)

# 通过P值以及HR对有预后意义的基因进行筛选

table(result$pvalue<0.05)

# FALSE TRUE

# 235 24

# outTab=result[result$pvalue<0.01,]

outTab=result[result$pvalue<0.05,]

genes=outTab$id

genes <- c("OS.time", "OS", genes)

#

# save(genes,file = "../单因素回归/手动单因素结果.Rdata")

# 森林图绘制：#读取输入文件

head(outTab)

data=outTab

# 将第一列变成行名：

rownames(data) <- data$id

data <- data[,-1]

head(data)

data$HR=as.numeric(data$HR)

data$HR.95L=as.numeric(data$HR.95L)

data$HR.95H=as.numeric(data$HR.95H)

data$pvalue=as.numeric(data$pvalue)

# rt <- data

hr <- sprintf("%.3f",rt$"HR")

hrLow <- sprintf("%.3f",rt$"HR.95L")

hrHigh <- sprintf("%.3f",rt$"HR.95H")

Hazard.ratio <- paste0(hr,"(",hrLow,"-",hrHigh,")")

pVal <- ifelse(rt$pvalue<0.001, "<0.001", sprintf("%.3f", rt$pvalue))

gene <- rownames(rt)

n <- nrow(rt)

nRow <- n+1

ylim <- c(1,nRow)

layout(matrix(c(1,2),nc=2),width=c(3,2.5))

xlim = c(0,3)

par(mar=c(4,2.5,2,1))

plot(1,xlim=xlim,ylim=ylim,type="n",axes=F,xlab="",ylab="")

text.cex=0.8

text(0,n:1,gene,adj=0,cex=text.cex)

text(1.5-0.5*0.2,n:1,pVal,adj=1,cex=text.cex);text(1.5-0.5*0.2,n+1,'pvalue',cex=text.cex,font=2,adj=1)

text(3,n:1,Hazard.ratio,adj=1,cex=text.cex);text(3,n+1,'Hazard ratio',cex=text.cex,font=2,adj=1,)

par(mar=c(4,1,2,1),mgp=c(2,0.5,0))

xlim = c(0,max(as.numeric(hrLow),as.numeric(hrHigh)))

plot(1,xlim=xlim,ylim=ylim,type="n",axes=F,ylab="",xaxs="i",xlab="Hazard ratio")

arrows(as.numeric(hrLow),n:1,as.numeric(hrHigh),n:1,angle=90,code=3,length=0.05,col="darkblue",lwd=2.5)

abline(v=1,col="black",lty=2,lwd=2)

boxcolor = ifelse((as.numeric(hr)>1)&(pVal<0.05),'red3',"green3")

points(as.numeric(hr), n:1, pch = 15, col = boxcolor, cex=1.3)

axis(1)

#

# 提取相同基因

comgene=Reduce(intersect,list(colnames(Train),colnames(TrainDataset),colnames(testDatasetGeo135565)))

Train=Train[,comgene]

TrainDataset=TrainDataset[,comgene]

testDatasetGeo135565=testDatasetGeo135565[,comgene]

# 只保留单因素回归后的基因

Train=Train[,genes]

TrainDataset=TrainDataset[,genes]

testDatasetGeo135565=testDatasetGeo135565[,genes]

#

str(Train)

Train$OS.time=as.numeric(Train$OS.time)

Train$OS=as.numeric(Train$OS)

#

TrainDataset$OS.time=as.numeric(TrainDataset$OS.time)

TrainDataset$OS=as.numeric(TrainDataset$OS)

testDatasetGeo135565$OS.time=as.numeric(testDatasetGeo135565$OS.time)

testDatasetGeo135565$OS=as.numeric(testDatasetGeo135565$OS)

#

trainlist=list(Train=Train,Test=TrainDataset

# ,Test3=testDatasetGeo135565

)

#

result <- data.frame()

rf_nodesize <- 5

seed <- 123

#### 1-1.RSF #################

#################################################################

set.seed(seed)

fit <- rfsrc(Surv(OS.time,OS)~.,data = trainlist$Train,

ntree = 1000,nodesize = rf_nodesize,

splitrule = 'logrank',

importance = T,

proximity = T,

forest = T,

seed = seed)

best <- which.min(fit$err.rate)

set.seed(seed)

fit <- rfsrc(Surv(OS.time,OS)~.,data = trainlist$Train,

ntree = best,nodesize = rf_nodesize,

splitrule = 'logrank',

importance = T,

proximity = T,

forest = T,

seed = seed)

rs <- lapply(trainlist,function(x){cbind(x[,1:2],RS=predict(fit,newdata = x)$predicted)}) # 要改， list中一共有几个数据集就写几

rs[["Train"]]=rs[["Train"]][rs[["Train"]]$RS !="Inf",]

rs[["Test"]]=rs[["Test"]][rs[["Test"]]$RS!="Inf",]

rs[["Train"]]=rs[["Train"]][rs[["Train"]]$RS !="-Inf",]

rs[["Test"]]=rs[["Test"]][rs[["Test"]]$RS!="-Inf",]

cc <- data.frame(Cindex=sapply(rs,function(x){as.numeric(summary(coxph(Surv(OS.time,OS)~RS,x))$concordance[1])}))%>%

rownames_to_column('ID')

cc$Model <- 'RSF'

result <- rbind(result,cc)

#################################################################

#### 1-2.rsf+Enet ################

#################################################################

#基因重要性

vi <- data.frame(imp=vimp.rfsrc(fit)$importance)

vi$imp <- (vi$imp-min(vi$imp))/(max(vi$imp)-min(vi$imp))

vi$ID <- rownames(vi)

#基因重要性可视化

pdf("rsf_highgene(rsf).pdf")

ggplot(vi,aes(imp,reorder(ID,imp)))+

geom_bar(stat = 'identity',fill='#FF9933',color='black',width=0.7)+

geom_vline(xintercept = 0.01,color='grey50',linetype=2)+

labs(x='Relative importance by Random Forest',y=NULL)+

theme_bw(base_rect_size = 1.5)+

theme(axis.text.x = element_text(size = 11,color='black'),

axis.text.y = element_text(size = 12,color='black'),

axis.title = element_text(size=13,color='black'),

legend.text = element_text(size=12,color='black'),

legend.title = element_text(size=13,color='black'))+

scale_y_discrete(expand = c(0.03,0.03))+

scale_x_continuous(expand = c(0.01,0.01))

dev.off()

#提取重要性大于0.01的基因

rid <- rownames(vi)[vi$imp>0.01]

train2 <- Train[,c('OS.time','OS',rid)] # 这边记得要改 训练集叫啥，就写啥

trainlist2 <- lapply(trainlist,function(x){x[,c('OS.time','OS',rid)]})

x1 <- as.matrix(train2[,rid])

x2 <- as.matrix(Surv(train2$OS.time,train2$OS))

#利用循环探索Enet最佳模型

for (alpha in seq(0,1,0.1)) {

set.seed(seed)

fit = cv.glmnet(x1, x2,family = "cox",alpha=alpha,nfolds = 10)

rs <- lapply(trainlist2,function(x){cbind(x[,1:2],RS=as.numeric(predict(fit,type='link',newx=as.matrix(x[,-c(1,2)]),s=fit$lambda.min)))}) # 要改

rs[["Train"]]=rs[["Train"]][rs[["Train"]]$RS !="Inf",]

rs[["Test"]]=rs[["Test"]][rs[["Test"]]$RS!="Inf",]

rs[["Train"]]=rs[["Train"]][rs[["Train"]]$RS !="-Inf",]

rs[["Test"]]=rs[["Test"]][rs[["Test"]]$RS!="-Inf",]

cc <- data.frame(Cindex=sapply(rs,function(x){as.numeric(summary(coxph(Surv(OS.time,OS)~RS,x))$concordance[1])}))%>%

rownames_to_column('ID')

cc$Model <- paste0('RSF + Enet','[α=',alpha,']')

result <- rbind(result,cc)

}

#利用交叉验证探索Enet最佳模型

set.seed(seed)

modelexp=as.matrix(trainlist2$Train[,c(3:ncol(trainlist2$Train))])

modelstat=Surv(trainlist2$Train$OS.time,trainlist2$Train$OS)

Enetmodel <- glmnet(modelexp,modelstat,family = 'cox',nfolds=10)

Enetmodel_cv<-cv.glmnet(modelexp,modelstat,family = 'cox',nfolds=10)

#建立最优模型

model_fit<-glmnet(modelexp,modelstat,family = 'cox',nfolds=10,keep=T,lambda = Enetmodel_cv$lambda.min)

rs <- lapply(trainlist2,function(x){cbind(x[,1:2],RS=as.numeric(predict(model_fit,type='link',newx=as.matrix(x[,-c(1,2)]),s=model_fit$lambda.min)))}) # 要改

cc <- data.frame(Cindex=sapply(rs,function(x){as.numeric(summary(coxph(Surv(OS.time,OS)~RS,x))$concordance[1])}))%>%

rownames_to_column('ID')

cc$Model <- paste0('RSF + Enet','[lambda=',round(Enetmodel_cv$lambda.min,3),']')

result <- rbind(result,cc)

####################################################################

####### 1-3.rsf+stepcox ####### #####

####################################################################

for (direction in c("both", "backward", "forward")) {

fit <- step(coxph(Surv(OS.time,OS)~.,train2),direction = direction)

rs <- lapply(trainlist2,function(x){cbind(x[,1:2],RS=predict(fit,type = 'risk',newdata = x))}) # 要改

rs[["Train"]]=rs[["Train"]][rs[["Train"]]$RS !="Inf",]

rs[["Test"]]=rs[["Test"]][rs[["Test"]]$RS!="Inf",]

rs[["Train"]]=rs[["Train"]][rs[["Train"]]$RS !="-Inf",]

rs[["Test"]]=rs[["Test"]][rs[["Test"]]$RS!="-Inf",]

cc <- data.frame(Cindex=sapply(rs,function(x){as.numeric(summary(coxph(Surv(OS.time,OS)~RS,x))$concordance[1])}))%>%

rownames_to_column('ID')

cc$Model <- paste0('RSF + StepCox','[',direction,']')

result <- rbind(result,cc)

}

#################################################################

#### 1-4.rsf+CoxBoost #### #######

#################################################################

set.seed(seed)

#计算最佳penalty

pen <- optimCoxBoostPenalty(train2[,'OS.time'],train2[,'OS'],as.matrix(train2[,-c(1,2)]),

trace=TRUE,start.penalty=500,parallel = T)

#计算最佳stepno

cv.res <- cv.CoxBoost(train2[,'OS.time'],train2[,'OS'],as.matrix(train2[,-c(1,2)]),

maxstepno=500,K=10,type="verweij",penalty=pen$penalty)

#构建最佳模型

fit <- CoxBoost(train2[,'OS.time'],train2[,'OS'],as.matrix(train2[,-c(1,2)]),

stepno=cv.res$optimal.step,penalty=pen$penalty)

rs <- lapply(trainlist2,function(x){cbind(x[,1:2],RS=as.numeric(predict(fit,newdata=x[,-c(1,2)], newtime=x[,1], newstatus=x[,2], type="lp")))}) # 要改

rs[["Train"]]=rs[["Train"]][rs[["Train"]]$RS !="Inf",]

rs[["Test"]]=rs[["Test"]][rs[["Test"]]$RS!="Inf",]

rs[["Train"]]=rs[["Train"]][rs[["Train"]]$RS !="-Inf",]

rs[["Test"]]=rs[["Test"]][rs[["Test"]]$RS!="-Inf",]

cc <- data.frame(Cindex=sapply(rs,function(x){as.numeric(summary(coxph(Surv(OS.time,OS)~RS,x))$concordance[1])}))%>%

rownames_to_column('ID')

cc$Model <- paste0('RSF + CoxBoost')

result <- rbind(result,cc)

#################################################################

#### 1-5.rsf+plsRcox #############

#################################################################

set.seed(seed)

#建立专用矩阵

model_exp=data.frame(train2[,-c(1:2)])

model_time=train2$OS.time

model_stat=train2$OS

#建立模型

model<-plsRcox(model_exp,time = model_time,event = model_stat,nt=10)

#进行交叉验证

cv.model<-cv.plsRcox(list(x=model_exp,time=model_time,status=model_stat),nt=5,verbose = T)

#构建最优模型

model<-plsRcox(model_exp,

time = model_time,

event = model_stat,

nt=cv.model$lambda.min5,

alpha.pvals.expli = 0.05,

sparse = T,

pvals.expli = T)

rs <- lapply(trainlist2,function(x){cbind(x[,1:2],RS=as.numeric(predict(model,type="lp",newdata=x[,-c(1,2)])))}) # 要改

rs[["Train"]]=rs[["Train"]][rs[["Train"]]$RS !="Inf",]

rs[["Test"]]=rs[["Test"]][rs[["Test"]]$RS!="Inf",]

rs[["Train"]]=rs[["Train"]][rs[["Train"]]$RS !="-Inf",]

rs[["Test"]]=rs[["Test"]][rs[["Test"]]$RS!="-Inf",]

cc <- data.frame(Cindex=sapply(rs,function(x){as.numeric(summary(coxph(Surv(OS.time,OS)~RS,x))$concordance[1])}))%>%

rownames_to_column('ID')

cc$Model <- paste0('RSF + plsRcox')

result <- rbind(result,cc)

#################################################################

#### 1-6.rsf+superpc #############

#################################################################

data <- list(x=t(train2[,-c(1,2)]),y=train2$OS.time,censoring.status=train2$OS,featurenames=colnames(train2)[-c(1,2)])

set.seed(seed)

fit <- superpc.train(data = data,type = 'survival',s0.perc = 0.5)

cv.fit <- superpc.cv(fit,data,n.threshold = 20,

n.fold = 10,

n.components=3,

min.features=5,

max.features=nrow(data$x),

compute.fullcv= TRUE,

compute.preval=TRUE)

rs <- lapply(trainlist2,function(w){

test <- list(x=t(w[,-c(1,2)]),y=w$OS.time,censoring.status=w$OS,featurenames=colnames(w)[-c(1,2)])

ff <- superpc.predict(fit,data,test,threshold = cv.fit$thresholds[which.max(cv.fit[["scor"]][1,])],n.components = 1)

rr <- as.numeric(ff$v.pred)

rr2 <- cbind(w[,1:2],RS=rr)

return(rr2)

})

rs[["Train"]]=rs[["Train"]][rs[["Train"]]$RS !="Inf",]

rs[["Test"]]=rs[["Test"]][rs[["Test"]]$RS!="Inf",]

rs[["Train"]]=rs[["Train"]][rs[["Train"]]$RS !="-Inf",]

rs[["Test"]]=rs[["Test"]][rs[["Test"]]$RS!="-Inf",]

cc <- data.frame(Cindex=sapply(rs,function(x){as.numeric(summary(coxph(Surv(OS.time,OS)~RS,x))$concordance[1])}))%>%

rownames_to_column('ID')

cc$Model <- paste0('RSF + SuperPC')

result <- rbind(result,cc)

#################################################################

#### 1-7.rsf+gbm #################

#################################################################

set.seed(seed)

fit <- gbm(formula = Surv(OS.time,OS)~.,

data = train2,

distribution = 'coxph',

n.minobsinnode = 10,

n.cores = 1,

n.trees = 1000,

shrinkage = 0.005,

interaction.depth = 2,

cv.folds = 5)

#构建最优模型

best <- which.min(fit$cv.error)

set.seed(seed)

fit <- gbm(formula = Surv(OS.time,OS)~.,

data = train2,

distribution = 'coxph',

n.trees = best,

interaction.depth = 3,

n.minobsinnode = 10,

shrinkage = 0.001,

cv.folds = 5,

n.cores = 1)

rs <- lapply(trainlist2,function(x){cbind(x[,1:2],RS=as.numeric(predict(fit,x,n.trees = best,type = 'link')))}) # 要改

rs[["Train"]]=rs[["Train"]][rs[["Train"]]$RS !="Inf",]

rs[["Test"]]=rs[["Test"]][rs[["Test"]]$RS!="Inf",]

rs[["Train"]]=rs[["Train"]][rs[["Train"]]$RS !="-Inf",]

rs[["Test"]]=rs[["Test"]][rs[["Test"]]$RS!="-Inf",]

cc <- data.frame(Cindex=sapply(rs,function(x){as.numeric(summary(coxph(Surv(OS.time,OS)~RS,x))$concordance[1])}))%>%

rownames_to_column('ID')

cc$Model <- paste0('RSF + GBM')

result <- rbind(result,cc)

#################################################################

#### 1-8.rsf+survivalsvm #########

#################################################################

set.seed(seed)

fit = survivalsvm(Surv(OS.time,OS)~., data= train2, gamma.mu = 2)

rs <- lapply(trainlist2,function(x){cbind(x[,1:2],RS=as.numeric(predict(fit, x)$predicted))}) # 要改

cc <- data.frame(Cindex=sapply(rs,function(x){as.numeric(summary(coxph(Surv(OS.time,OS)~RS,x))$concordance[1])}))%>%

rownames_to_column('ID')

cc$Model <- paste0('RSF + survival-SVM')

result <- rbind(result,cc)

#################################################################

#### 1-9.rsf+Ridge ###############

#################################################################

set.seed(seed)

modelexp=as.matrix(train2[,c(3:ncol(train2))])

#利用循环探索Ridge最佳模型

for (alpha in seq(0,1,0.1)) {

set.seed(seed)

model <- glmnet(modelexp,train2$OS,family = 'binomial',alpha = alpha,nfolds=10)

model_cv<-cv.glmnet(modelexp,train2$OS,family = 'binomial',alpha =alpha,nfolds=10)

fit<-glmnet(modelexp,train2$OS,family = 'binomial',alpha = alpha,nfolds=10,keep=T,lambda = model_cv$lambda.min)

rs <- lapply(trainlist2,function(x){cbind(x[,1:2],RS=as.numeric(predict(fit,type="response",newx=as.matrix(x[,-c(1,2)]))))}) # 要改

rs[["Train"]]=rs[["Train"]][rs[["Train"]]$RS !="Inf",]

rs[["Test"]]=rs[["Test"]][rs[["Test"]]$RS!="Inf",]

rs[["Train"]]=rs[["Train"]][rs[["Train"]]$RS !="-Inf",]

rs[["Test"]]=rs[["Test"]][rs[["Test"]]$RS!="-Inf",]

cc <- data.frame(Cindex=sapply(rs,function(x){as.numeric(summary(coxph(Surv(OS.time,OS)~RS,x))$concordance[1])}))%>%

rownames_to_column('ID')

cc$Model <- paste0('RSF + Ridge','[α=',alpha,']')

result <- rbind(result,cc)

}

# #################################################################

# #### 1-10.rsf+obliqueRSF #########

# #################################################################

# set.seed(seed)

# model<-orsf(data = train2,n_tree = 100,formula = Surv(OS.time,OS)~.)

# rs <- lapply(trainlist2,function(x){cbind(x[,1:2],RS=as.numeric(predict(model, new_data=x,pred_type = "risk")[,1]))}) # 要改

# rs[["Train"]]=rs[["Train"]][rs[["Train"]]$RS !="Inf",]

# rs[["Test"]]=rs[["Test"]][rs[["Test"]]$RS!="Inf",]

# rs[["Train"]]=rs[["Train"]][rs[["Train"]]$RS !="-Inf",]

# rs[["Test"]]=rs[["Test"]][rs[["Test"]]$RS!="-Inf",]

# cc <- data.frame(Cindex=sapply(rs,function(x){as.numeric(summary(coxph(Surv(OS.time,OS)~RS,x))$concordance[1])}))%>%

# rownames_to_column('ID')

# cc$Model <- paste0('RSF + obliqueRSF')

# result <- rbind(result,cc)

#################################################################

#### 1-11.rsf+xgboost ############

#################################################################

set.seed(seed)

#建立专用矩阵

model_mat<-xgb.DMatrix(data = as.matrix(train2[,-c(1:2)]),label=train2$OS.time)

#构建参数

object<-list(bojective="surivival:cox",

booster="gbtree",

eval_metric="cox-nloglik",

eta=0.01,

max_depth=3,

subsample=1,

colsample_bytree=1,

gamma=0.5)

#构建模型

model<-xgb.train(params=object,data = model_mat,nrounds = 100,watchlist = list(val2=model_mat),early_stopping_rounds = 10)

rs <- lapply(trainlist2,function(x){cbind(x[,1:2],RS=as.numeric(predict(model, newdata=as.matrix(x[,-c(1:2)]))))}) # 要改

rs[["Train"]]=rs[["Train"]][rs[["Train"]]$RS !="Inf",]

rs[["Test"]]=rs[["Test"]][rs[["Test"]]$RS!="Inf",]

rs[["Train"]]=rs[["Train"]][rs[["Train"]]$RS !="-Inf",]

rs[["Test"]]=rs[["Test"]][rs[["Test"]]$RS!="-Inf",]

cc <- data.frame(Cindex=sapply(rs,function(x){as.numeric(summary(coxph(Surv(OS.time,OS)~RS,x))$concordance[1])}))%>%

rownames_to_column('ID')

cc$Model <- paste0('RSF + xgboost')

result <- rbind(result,cc)

#################################################################

#### 1-12.rsf+CForest#############

#################################################################

set.seed(seed)

model<-party::cforest(Surv(OS.time,OS)~.,data=train2,controls = cforest_unbiased(ntree=50))

rs <- lapply(trainlist2,function(x){cbind(x[,1:2],RS=as.numeric(predict(model, newdata=x,type = "response")))})

rs[["Train"]]=rs[["Train"]][rs[["Train"]]$RS !="Inf",]

rs[["Test"]]=rs[["Test"]][rs[["Test"]]$RS!="Inf",]

rs[["Train"]]=rs[["Train"]][rs[["Train"]]$RS !="-Inf",]

rs[["Test"]]=rs[["Test"]][rs[["Test"]]$RS!="-Inf",]

cc <- data.frame(Cindex=sapply(rs,function(x){as.numeric(summary(coxph(Surv(OS.time,OS)~RS,x))$concordance[1])}))%>%

rownames_to_column('ID')

cc$Model <- paste0('RSF + CForest')

result <- rbind(result,cc)

#################################################################

#### 1-13.rsf+CTree###############

#################################################################

set.seed(seed)

#建立模型

model<-ctree(Surv(OS.time,OS)~.,data=train2)

#计算变量重要性

rs <- lapply(trainlist2,function(x){cbind(x[,1:2],RS=as.numeric(predict(model, newdata=x,type = "response")))})

rs[["Train"]]=rs[["Train"]][rs[["Train"]]$RS !="Inf",]

rs[["Test"]]=rs[["Test"]][rs[["Test"]]$RS!="Inf",]

rs[["Train"]]=rs[["Train"]][rs[["Train"]]$RS !="-Inf",]

rs[["Test"]]=rs[["Test"]][rs[["Test"]]$RS!="-Inf",]

cc <- data.frame(Cindex=sapply(rs,function(x){as.numeric(summary(coxph(Surv(OS.time,OS)~RS,x))$concordance[1])}))%>%

rownames_to_column('ID')

cc$Model <- paste0('RSF + CTree')

result <- rbind(result,cc)

#################################################################

#### 2-1.Enet ####################

#################################################################

modelexp=as.matrix(Train[,c(3:ncol(Train))]) # 要改，train

modelstat=Surv(Train$OS.time,Train$OS)

for (alpha in seq(0,1,0.1)) {

set.seed(seed)

model <- glmnet(modelexp,modelstat,family = 'cox',alpha = alpha,nfolds=10)

model_cv<-cv.glmnet(modelexp,modelstat,family = 'cox',alpha = alpha,nfolds=10)

fit <-glmnet(modelexp,modelstat,family = 'cox',alpha =alpha,nfolds=10,keep=T,lambda = model_cv$lambda.min)

rs <- lapply(trainlist,function(x){cbind(x[,1:2],RS=as.numeric(predict(fit,type='link',newx=as.matrix(x[,-c(1,2)]),s=fit$lambda.min)))})

rs[["Train"]]=rs[["Train"]][rs[["Train"]]$RS !="Inf",]

rs[["Test"]]=rs[["Test"]][rs[["Test"]]$RS!="Inf",]

rs[["Train"]]=rs[["Train"]][rs[["Train"]]$RS !="-Inf",]

rs[["Test"]]=rs[["Test"]][rs[["Test"]]$RS!="-Inf",]

cc <- data.frame(Cindex=sapply(rs,function(x){as.numeric(summary(coxph(Surv(OS.time,OS)~RS,x))$concordance[1])}))%>%

rownames_to_column('ID')

cc$Model <- paste0('Enet','[α=',alpha,']')

result <- rbind(result,cc)

}

#################################################################

#### 2-2.Lasso+RSF################

#################################################################

set.seed(seed)

fit = cv.glmnet(modelexp, modelstat,family = "cox")

coef.min = coef(fit, s = "lambda.min")

index=which(coef.min!=0)

actCoef=coef.min[index]

rid=row.names(coef.min)[index] # lasso筛选了

# 提取最佳模型的系数

best_model_coef <- coef(fit, s = fit$lambda.min)

# 10 x 1 sparse Matrix of class "dgCMatrix"

# 1

# BGN 0.05777262

# GADD45B .

# JUN 0.41937235

# MMP14 .

# NOTCH3 0.24966085

# PMEPA1 0.27412902

# PPDPF .

# SDC1 0.05284677

# TBX2 0.25064196

# UQCRQ 0.49472101

# rid <- coef.min@Dimnames[[1]] # 这取得是全部基因，没有筛选

train2 <- Train[,c('OS.time','OS',rid)] # 要改

trainlist2 <- lapply(trainlist,function(x){x[,c('OS.time','OS',rid)]})

set.seed(seed)

fit <- rfsrc(Surv(OS.time,OS)~.,data = train2,

ntree = 1000,nodesize = rf_nodesize,##该值建议多调整

splitrule = 'logrank',

importance = T,

proximity = T,

forest = T,

seed = seed)

best <- which.min(fit$err.rate)

set.seed(seed)

fit <- rfsrc(Surv(OS.time,OS)~.,data = train2,

ntree = best,nodesize = rf_nodesize,##该值建议多调整

splitrule = 'logrank',

importance = T,

proximity = T,

forest = T,

seed = seed)

rs <- lapply(trainlist2,function(x){cbind(x[,1:2],RS=predict(fit,newdata = x)$predicted)})

rs[["Train"]]=rs[["Train"]][rs[["Train"]]$RS !="Inf",]

rs[["Test"]]=rs[["Test"]][rs[["Test"]]$RS!="Inf",]

rs[["Train"]]=rs[["Train"]][rs[["Train"]]$RS !="-Inf",]

rs[["Test"]]=rs[["Test"]][rs[["Test"]]$RS!="-Inf",]

cc <- data.frame(Cindex=sapply(rs,function(x){as.numeric(summary(coxph(Surv(OS.time,OS)~RS,x))$concordance[1])}))%>%

rownames_to_column('ID')

cc$Model <- 'Lasso + RSF'

result <- rbind(result,cc)

##################################################################

#### 2-3.Lasso+StepCox ############

##################################################################

for (direction in c("both", "backward", "forward")) {

fit <- step(coxph(Surv(OS.time,OS)~.,train2),direction = direction)

rs <- lapply(trainlist2,function(x){cbind(x[,1:2],RS=predict(fit,type = 'risk',newdata = x))})

rs[["Train"]]=rs[["Train"]][rs[["Train"]]$RS !="Inf",]

rs[["Test"]]=rs[["Test"]][rs[["Test"]]$RS!="Inf",]

rs[["Train"]]=rs[["Train"]][rs[["Train"]]$RS !="-Inf",]

rs[["Test"]]=rs[["Test"]][rs[["Test"]]$RS!="-Inf",]

cc <- data.frame(Cindex=sapply(rs,function(x){as.numeric(summary(coxph(Surv(OS.time,OS)~RS,x))$concordance[1])}))%>%

rownames_to_column('ID')

cc$Model <- paste0('Lasso + StepCox','[',direction,']')

result <- rbind(result,cc)

}

####################################################################

#### 2-4.Lasso+CoxBoost #############

####################################################################

set.seed(seed)

#计算最佳penalty

modelpen<-optimCoxBoostPenalty(time = train2$OS.time,

status = train2$OS,

as.matrix(train2[,-c(1:2)]),

trace = T,

parallel = T)

#计算最佳stepno

cvmodel<-cv.CoxBoost(time = train2$OS.time,

status = train2$OS,

as.matrix(train2[,-c(1:2)]),

maxstepno = 100,

K = 3,

type = "verweij",

penalty=modelpen$penalty)

#构建CoxBoost模型

fit<-CoxBoost(time = train2$OS.time,

status = train2$OS,

as.matrix(train2[,-c(1:2)]),

stepno = cvmodel$optimal.step,

penalty = modelpen$penalty)

rs <- lapply(trainlist2,function(x){cbind(x[,1:2],RS=as.numeric(predict(fit,newdata=x[,-c(1,2)], newtime=x[,1], newstatus=x[,2], type="lp")))})

rs[["Train"]]=rs[["Train"]][rs[["Train"]]$RS !="Inf",]

rs[["Test"]]=rs[["Test"]][rs[["Test"]]$RS!="Inf",]

rs[["Train"]]=rs[["Train"]][rs[["Train"]]$RS !="-Inf",]

rs[["Test"]]=rs[["Test"]][rs[["Test"]]$RS!="-Inf",]

cc <- data.frame(Cindex=sapply(rs,function(x){as.numeric(summary(coxph(Surv(OS.time,OS)~RS,x))$concordance[1])}))%>%

rownames_to_column('ID')

cc$Model <- paste0('Lasso + CoxBoost')

result <- rbind(result,cc)

##################################################################

#### 2-5.Lasso+plsRcox ############

##################################################################

set.seed(seed)

#建立专用矩阵

model_exp=data.frame(train2[,-c(1:2)])

model_time=train2$OS.time

model_stat=train2$OS

#建立模型

model<-plsRcox(model_exp,time = model_time,event = model_stat,nt=10)

#进行交叉验证

cv.model<-cv.plsRcox(list(x=model_exp,time=model_time,status=model_stat),nt=5,verbose = F)

#构建最优模型

cv.plsRcox.res=cv.plsRcox(list(x=model_exp,time=model_time,status=model_stat),nt=5,verbose = F)

fit <- plsRcox(model_exp,

time = model_time,

event = model_stat,

nt=cv.model$lambda.min5,

alpha.pvals.expli = 0.05,

sparse = T,

pvals.expli = T)

rs <- lapply(trainlist2,function(x){cbind(x[,1:2],RS=as.numeric(predict(fit,type="lp",newdata=x[,-c(1,2)])))})

rs[["Train"]]=rs[["Train"]][rs[["Train"]]$RS !="Inf",]

rs[["Test"]]=rs[["Test"]][rs[["Test"]]$RS!="Inf",]

rs[["Train"]]=rs[["Train"]][rs[["Train"]]$RS !="-Inf",]

rs[["Test"]]=rs[["Test"]][rs[["Test"]]$RS!="-Inf",]

cc <- data.frame(Cindex=sapply(rs,function(x){as.numeric(summary(coxph(Surv(OS.time,OS)~RS,x))$concordance[1])}))%>%

rownames_to_column('ID')

cc$Model <- paste0('Lasso + plsRcox')

result <- rbind(result,cc)

##################################################################

#### 2-6.Lasso+superpc ############

##################################################################

data <- list(x=t(train2[,-c(1,2)]),y=train2$OS.time,censoring.status=train2$OS,featurenames=colnames(train2)[-c(1,2)])

set.seed(seed)

fit <- superpc.train(data = data,type = 'survival',s0.perc = 0.5)

cv.fit <- superpc.cv(fit,data,n.threshold = 20,

n.fold = 10,

n.components=3,

min.features=5,

max.features=nrow(data$x),

compute.fullcv= TRUE,

compute.preval=TRUE)

rs <- lapply(trainlist2,function(w){

test <- list(x=t(w[,-c(1,2)]),y=w$OS.time,censoring.status=w$OS,featurenames=colnames(w)[-c(1,2)])

ff <- superpc.predict(fit,data,test,threshold = cv.fit$thresholds[which.max(cv.fit[["scor"]][1,])],n.components = 1)

rr <- as.numeric(ff$v.pred)

rr2 <- cbind(w[,1:2],RS=rr)

return(rr2)

})

rs[["Train"]]=rs[["Train"]][rs[["Train"]]$RS !="Inf",]

rs[["Test"]]=rs[["Test"]][rs[["Test"]]$RS!="Inf",]

rs[["Train"]]=rs[["Train"]][rs[["Train"]]$RS !="-Inf",]

rs[["Test"]]=rs[["Test"]][rs[["Test"]]$RS!="-Inf",]

cc <- data.frame(Cindex=sapply(rs,function(x){as.numeric(summary(coxph(Surv(OS.time,OS)~RS,x))$concordance[1])}))%>%

rownames_to_column('ID')

cc$Model <- paste0('Lasso + SuperPC')

result <- rbind(result,cc)

#################################################################

#### 2-7.Lasso+gbm ###############

#################################################################

set.seed(seed)

fit <- gbm(formula = Surv(OS.time,OS)~.,data = train2,distribution = 'coxph',

n.trees = 1000,

interaction.depth = 3,

n.minobsinnode = 10,

shrinkage = 0.001,

cv.folds = 5,n.cores = 1)

best <- which.min(fit$cv.error)

set.seed(seed)

fit <- gbm(formula = Surv(OS.time,OS)~.,data = train2,distribution = 'coxph',

n.trees = best,

interaction.depth = 3,

n.minobsinnode = 10,

shrinkage = 0.001,

cv.folds = 5,n.cores = 1)

rs <- lapply(trainlist2,function(x){cbind(x[,1:2],RS=as.numeric(predict(fit,x,n.trees = best,type = 'link')))})

rs[["Train"]]=rs[["Train"]][rs[["Train"]]$RS !="Inf",]

rs[["Test"]]=rs[["Test"]][rs[["Test"]]$RS!="Inf",]

rs[["Train"]]=rs[["Train"]][rs[["Train"]]$RS !="-Inf",]

rs[["Test"]]=rs[["Test"]][rs[["Test"]]$RS!="-Inf",]

cc <- data.frame(Cindex=sapply(rs,function(x){as.numeric(summary(coxph(Surv(OS.time,OS)~RS,x))$concordance[1])}))%>%

rownames_to_column('ID')

cc$Model <- paste0('Lasso + GBM')

result <- rbind(result,cc)

#####################################################################

#### 2-8.Lasso+survivalsvm ###########

#####################################################################

set.seed(seed)

fit = survivalsvm(Surv(OS.time,OS)~., data= train2, gamma.mu = 2)

rs <- lapply(trainlist2,function(x){cbind(x[,1:2],RS=as.numeric(predict(fit, x)$predicted))})

rs[["Train"]]=rs[["Train"]][rs[["Train"]]$RS !="Inf",]

rs[["Test"]]=rs[["Test"]][rs[["Test"]]$RS!="Inf",]

rs[["Train"]]=rs[["Train"]][rs[["Train"]]$RS !="-Inf",]

rs[["Test"]]=rs[["Test"]][rs[["Test"]]$RS!="-Inf",]

cc <- data.frame(Cindex=sapply(rs,function(x){as.numeric(summary(coxph(Surv(OS.time,OS)~RS,x))$concordance[1])}))%>%

rownames_to_column('ID')

cc$Model <- paste0('Lasso + survival-SVM')

result <- rbind(result,cc)

#################################################################

#### 2-9.Lasso+Ridge #############

#################################################################

set.seed(seed)

modelexp=as.matrix(train2[,c(3:ncol(train2))])

#利用循环探索Ridge最佳模型

for (alpha in seq(0,1,0.1)) {

set.seed(seed)

model <- glmnet(modelexp,train2$OS,family = 'binomial',alpha = alpha,nfolds=10)

model_cv<-cv.glmnet(modelexp,train2$OS,family = 'binomial',alpha =alpha,nfolds=10)

fit<-glmnet(modelexp,train2$OS,family = 'binomial',alpha = alpha,nfolds=10,keep=T,lambda = model_cv$lambda.min)

rs <- lapply(trainlist2,function(x){cbind(x[,1:2],RS=as.numeric(predict(fit,type="response",newx=as.matrix(x[,-c(1,2)]))))})

rs[["Train"]]=rs[["Train"]][rs[["Train"]]$RS !="Inf",]

rs[["Test"]]=rs[["Test"]][rs[["Test"]]$RS!="Inf",]

rs[["Train"]]=rs[["Train"]][rs[["Train"]]$RS !="-Inf",]

rs[["Test"]]=rs[["Test"]][rs[["Test"]]$RS!="-Inf",]

cc <- data.frame(Cindex=sapply(rs,function(x){as.numeric(summary(coxph(Surv(OS.time,OS)~RS,x))$concordance[1])}))%>%

rownames_to_column('ID')

cc$Model <- paste0('Lasso + Ridge','[α=',alpha,']')

result <- rbind(result,cc)

}

# ###################################################################

# #### 2-10.Lasso+obliqueRSF #########

# ###################################################################

# set.seed(seed)

# model<-orsf(data = train2,n_tree = 100,formula = Surv(OS.time,OS)~.)

# rs <- lapply(trainlist2,function(x){cbind(x[,1:2],RS=as.numeric(predict(model, new_data=x,pred_type = "risk")[,1]))})

# rs[["Train"]]=rs[["Train"]][rs[["Train"]]$RS !="Inf",]

# rs[["Test"]]=rs[["Test"]][rs[["Test"]]$RS!="Inf",]

# rs[["Train"]]=rs[["Train"]][rs[["Train"]]$RS !="-Inf",]

# rs[["Test"]]=rs[["Test"]][rs[["Test"]]$RS!="-Inf",]

# cc <- data.frame(Cindex=sapply(rs,function(x){as.numeric(summary(coxph(Surv(OS.time,OS)~RS,x))$concordance[1])}))%>%

# rownames_to_column('ID')

# cc$Model <- paste0('Lasso + obliqueRSF')

# result <- rbind(result,cc)

#################################################################

#### 2-11.Lasso+xgboost ##########

#################################################################

set.seed(seed)

#建立专用矩阵

model_mat<-xgb.DMatrix(data = as.matrix(train2[,-c(1:2)]),label=train2$OS.time)

#构建参数

object<-list(bojective="surivival:cox",

booster="gbtree",

eval_metric="cox-nloglik",

eta=0.01,

max_depth=3,

subsample=1,

colsample_bytree=1,

gamma=0.5)

#构建模型

model<-xgb.train(params=object,data = model_mat,nrounds = 100,watchlist = list(val2=model_mat),early_stopping_rounds = 10)

rs <- lapply(trainlist2,function(x){cbind(x[,1:2],RS=as.numeric(predict(model, newdata=as.matrix(x[,-c(1:2)]))))})

rs[["Train"]]=rs[["Train"]][rs[["Train"]]$RS !="Inf",]

rs[["Test"]]=rs[["Test"]][rs[["Test"]]$RS!="Inf",]

rs[["Train"]]=rs[["Train"]][rs[["Train"]]$RS !="-Inf",]

rs[["Test"]]=rs[["Test"]][rs[["Test"]]$RS!="-Inf",]

cc <- data.frame(Cindex=sapply(rs,function(x){as.numeric(summary(coxph(Surv(OS.time,OS)~RS,x))$concordance[1])}))%>%

rownames_to_column('ID')

cc$Model <- paste0('Lasso + xgboost')

result <- rbind(result,cc)

#################################################################

#### 2-12.Lasso+CForest###########

#################################################################

set.seed(seed)

model<-party::cforest(Surv(OS.time,OS)~.,data=train2,controls = cforest_unbiased(ntree=50))

rs <- lapply(trainlist2,function(x){cbind(x[,1:2],RS=as.numeric(predict(model, newdata=x,type = "response")))})

rs[["Train"]]=rs[["Train"]][rs[["Train"]]$RS !="Inf",]

rs[["Test"]]=rs[["Test"]][rs[["Test"]]$RS!="Inf",]

rs[["Train"]]=rs[["Train"]][rs[["Train"]]$RS !="-Inf",]

rs[["Test"]]=rs[["Test"]][rs[["Test"]]$RS!="-Inf",]

cc <- data.frame(Cindex=sapply(rs,function(x){as.numeric(summary(coxph(Surv(OS.time,OS)~RS,x))$concordance[1])}))%>%

rownames_to_column('ID')

cc$Model <- paste0('Lasso + CForest')

result <- rbind(result,cc)

#################################################################

#### 2-13.Lasso+CTree#############

#################################################################

set.seed(seed)

model<-ctree(Surv(OS.time,OS)~.,data=train2)

rs <- lapply(trainlist2,function(x){cbind(x[,1:2],RS=as.numeric(predict(model, newdata=x,type = "response")))})

rs[["Train"]]=rs[["Train"]][rs[["Train"]]$RS !="Inf",]

rs[["Test"]]=rs[["Test"]][rs[["Test"]]$RS!="Inf",]

rs[["Train"]]=rs[["Train"]][rs[["Train"]]$RS !="-Inf",]

rs[["Test"]]=rs[["Test"]][rs[["Test"]]$RS!="-Inf",]

cc <- data.frame(Cindex=sapply(rs,function(x){as.numeric(summary(coxph(Surv(OS.time,OS)~RS,x))$concordance[1])}))%>%

rownames_to_column('ID')

cc$Model <- paste0('Lasso + CTree')

result <- rbind(result,cc)

#################################################################

#### 3-1.StepCox #################

#################################################################

for (direction in c("both", "backward", "forward")) {

fit <- step(coxph(Surv(OS.time,OS)~.,Train),direction = direction) # 要改

rs <- lapply(trainlist,function(x){cbind(x[,1:2],RS=predict(fit,type = 'risk',newdata = x))})

rs[["Train"]]=rs[["Train"]][rs[["Train"]]$RS !="Inf",]

rs[["Test"]]=rs[["Test"]][rs[["Test"]]$RS!="Inf",]

rs[["Train"]]=rs[["Train"]][rs[["Train"]]$RS !="-Inf",]

rs[["Test"]]=rs[["Test"]][rs[["Test"]]$RS!="-Inf",]

cc <- data.frame(Cindex=sapply(rs,function(x){as.numeric(summary(coxph(Surv(OS.time,OS)~RS,x))$concordance[1])}))%>%

rownames_to_column('ID')

cc$Model <- paste0('StepCox','[',direction,']')

result <- rbind(result,cc)

}

#################################################################

#### 3-2.StepCox+RSF #############

#################################################################

for (direction in c("both", "backward")) {

fit <- step(coxph(Surv(OS.time,OS)~.,Train),direction = direction) # 要改

rid <- names(coef(fit))

train2 <- Train[,c('OS.time','OS',rid)] # 要改

trainlist2 <- lapply(trainlist,function(x){x[,c('OS.time','OS',rid)]})

set.seed(seed)

fit <- rfsrc(Surv(OS.time,OS)~.,data = train2,

ntree = 1000,nodesize = rf_nodesize,##该值建议多调整

splitrule = 'logrank',

importance = T,

proximity = T,

forest = T,

seed = seed)

best <- which.min(fit$err.rate)

set.seed(seed)

fit <- rfsrc(Surv(OS.time,OS)~.,data = train2,

ntree = best,nodesize = rf_nodesize,##该值建议多调整

splitrule = 'logrank',

importance = T,

proximity = T,

forest = T,

seed = seed)

rs <- lapply(trainlist2,function(x){cbind(x[,1:2],RS=predict(fit,newdata = x)$predicted)})

rs[["Train"]]=rs[["Train"]][rs[["Train"]]$RS !="Inf",]

rs[["Test"]]=rs[["Test"]][rs[["Test"]]$RS!="Inf",]

rs[["Train"]]=rs[["Train"]][rs[["Train"]]$RS !="-Inf",]

rs[["Test"]]=rs[["Test"]][rs[["Test"]]$RS!="-Inf",]

cc <- data.frame(Cindex=sapply(rs,function(x){as.numeric(summary(coxph(Surv(OS.time,OS)~RS,x))$concordance[1])}))%>%

rownames_to_column('ID')

cc$Model <- paste0('StepCox','[',direction,']',' + RSF')

result <- rbind(result,cc)

}

#################################################################

#### 3-3.StepCox+Enet ###########

#################################################################

for (direction in c("both", "backward")) {

fit <- step(coxph(Surv(OS.time,OS)~.,Train),direction = direction)

rid <- names(coef(fit))

train2 <- Train[,c('OS.time','OS',rid)]

trainlist2 <- lapply(trainlist,function(x){x[,c('OS.time','OS',rid)]})

x1 <- as.matrix(train2[,rid])

x2 <- as.matrix(Surv(train2$OS.time,train2$OS))

for (alpha in seq(0,1,0.1)) {

set.seed(seed)

fit = cv.glmnet(x1, x2,family = "cox",alpha=alpha,nfolds = 10)

rs <- lapply(trainlist2,function(x){cbind(x[,1:2],RS=as.numeric(predict(fit,type='link',newx=as.matrix(x[,-c(1,2)]),s=fit$lambda.min)))})

rs[["Train"]]=rs[["Train"]][rs[["Train"]]$RS !="Inf",]

rs[["Test"]]=rs[["Test"]][rs[["Test"]]$RS!="Inf",]

rs[["Train"]]=rs[["Train"]][rs[["Train"]]$RS !="-Inf",]

rs[["Test"]]=rs[["Test"]][rs[["Test"]]$RS!="-Inf",]

cc <- data.frame(Cindex=sapply(rs,function(x){as.numeric(summary(coxph(Surv(OS.time,OS)~RS,x))$concordance[1])}))%>%

rownames_to_column('ID')

cc$Model <- paste0('StepCox','[',direction,']',' + Enet','[α=',alpha,']')

result <- rbind(result,cc)

}

}

##################################################################

#### 3-4.StepCox+CoxBoost #########

##################################################################

for (direction in c("both", "backward")) {

fit <- step(coxph(Surv(OS.time,OS)~.,Train),direction = direction)

rid <- names(coef(fit))

train2 <- Train[,c('OS.time','OS',rid)]

trainlist2 <- lapply(trainlist,function(x){x[,c('OS.time','OS',rid)]})

set.seed(seed)

pen <- optimCoxBoostPenalty(train2[,'OS.time'],train2[,'OS'],as.matrix(train2[,-c(1,2)]),

trace=TRUE,start.penalty=500,parallel = T)

cv.res <- cv.CoxBoost(train2[,'OS.time'],train2[,'OS'],as.matrix(train2[,-c(1,2)]),

maxstepno=500,K=10,type="verweij",penalty=pen$penalty)

fit <- CoxBoost(train2[,'OS.time'],train2[,'OS'],as.matrix(train2[,-c(1,2)]),

stepno=cv.res$optimal.step,penalty=pen$penalty)

rs <- lapply(trainlist2,function(x){cbind(x[,1:2],RS=as.numeric(predict(fit,newdata=x[,-c(1,2)], newtime=x[,1], newstatus=x[,2], type="lp")))})

rs[["Train"]]=rs[["Train"]][rs[["Train"]]$RS !="Inf",]

rs[["Test"]]=rs[["Test"]][rs[["Test"]]$RS!="Inf",]

rs[["Train"]]=rs[["Train"]][rs[["Train"]]$RS !="-Inf",]

rs[["Test"]]=rs[["Test"]][rs[["Test"]]$RS!="-Inf",]

cc <- data.frame(Cindex=sapply(rs,function(x){as.numeric(summary(coxph(Surv(OS.time,OS)~RS,x))$concordance[1])}))%>%

rownames_to_column('ID')

cc$Model <- paste0('StepCox','[',direction,']',' + CoxBoost')

result <- rbind(result,cc)

}

#################################################################

#### 3-5.StepCox+plsRcox #########

#################################################################

for (direction in c("both", "backward")) {

fit <- step(coxph(Surv(OS.time,OS)~.,Train),direction = direction)

rid <- names(coef(fit))

train2 <- Train[,c('OS.time','OS',rid)]

trainlist2 <- lapply(trainlist,function(x){x[,c('OS.time','OS',rid)]})

set.seed(seed)

cv.plsRcox.res=cv.plsRcox(list(x=train2[,rid],time=train2$OS.time,status=train2$OS),nt=10,nfold = 10,verbose = F)

fit <- plsRcox(train2[,rid],time=train2$OS.time,event=train2$OS,nt=as.numeric(cv.plsRcox.res[5]))

rs <- lapply(trainlist2,function(x){cbind(x[,1:2],RS=as.numeric(predict(fit,type="lp",newdata=x[,-c(1,2)])))})

rs[["Train"]]=rs[["Train"]][rs[["Train"]]$RS !="Inf",]

rs[["Test"]]=rs[["Test"]][rs[["Test"]]$RS!="Inf",]

rs[["Train"]]=rs[["Train"]][rs[["Train"]]$RS !="-Inf",]

rs[["Test"]]=rs[["Test"]][rs[["Test"]]$RS!="-Inf",]

cc <- data.frame(Cindex=sapply(rs,function(x){as.numeric(summary(coxph(Surv(OS.time,OS)~RS,x))$concordance[1])}))%>%

rownames_to_column('ID')

cc$Model <- paste0('StepCox','[',direction,']',' + plsRcox')

result <- rbind(result,cc)

}

#################################################################

#### 3-6.StepCox+superpc #########

#################################################################

for (direction in c("both", "backward")) {

fit <- step(coxph(Surv(OS.time,OS)~.,Train),direction = direction)

rid <- names(coef(fit))

train2 <- Train[,c('OS.time','OS',rid)]

trainlist2 <- lapply(trainlist,function(x){x[,c('OS.time','OS',rid)]})

data <- list(x=t(train2[,-c(1,2)]),y=train2$OS.time,censoring.status=train2$OS,featurenames=colnames(train2)[-c(1,2)])

set.seed(seed)

fit <- superpc.train(data = data,type = 'survival',s0.perc = 0.5)

cv.fit <- superpc.cv(fit,data,n.threshold = 20,

n.fold = 5,

n.components=3,

min.features=1,

max.features=nrow(data$x),

compute.fullcv= TRUE,

compute.preval=TRUE)

rs <- lapply(trainlist2,function(w){

test <- list(x=t(w[,-c(1,2)]),y=w$OS.time,censoring.status=w$OS,featurenames=colnames(w)[-c(1,2)])

ff <- superpc.predict(fit,data,test,threshold = cv.fit$thresholds[which.max(cv.fit[["scor"]][1,])],n.components = 1)

rr <- as.numeric(ff$v.pred)

rr2 <- cbind(w[,1:2],RS=rr)

return(rr2)

})

rs[["Train"]]=rs[["Train"]][rs[["Train"]]$RS !="Inf",]

rs[["Test"]]=rs[["Test"]][rs[["Test"]]$RS!="Inf",]

rs[["Train"]]=rs[["Train"]][rs[["Train"]]$RS !="-Inf",]

rs[["Test"]]=rs[["Test"]][rs[["Test"]]$RS!="-Inf",]

cc <- data.frame(Cindex=sapply(rs,function(x){as.numeric(summary(coxph(Surv(OS.time,OS)~RS,x))$concordance[1])}))%>%

rownames_to_column('ID')

cc$Model <- paste0('StepCox','[',direction,']',' + SuperPC')

result <- rbind(result,cc)

}

#############################################################

#### 3-7.StepCox+gbm #########

#############################################################

for (direction in c("both", "backward")) {

fit <- step(coxph(Surv(OS.time,OS)~.,Train),direction = direction)

rid <- names(coef(fit))

train2 <- Train[,c('OS.time','OS',rid)]

trainlist2 <- lapply(trainlist,function(x){x[,c('OS.time','OS',rid)]})

set.seed(seed)

fit <- gbm(formula = Surv(OS.time,OS)~.,data = train2,distribution = 'coxph',

n.trees = 1000,

interaction.depth = 3,

n.minobsinnode = 10,

shrinkage = 0.001,

cv.folds = 5,n.cores = 1)

best <- which.min(fit$cv.error)

set.seed(seed)

fit <- gbm(formula = Surv(OS.time,OS)~.,data = train2,distribution = 'coxph',

n.trees = best,

interaction.depth = 3,

n.minobsinnode = 10,

shrinkage = 0.001,

cv.folds = 5,n.cores = 1)

rs <- lapply(trainlist2,function(x){cbind(x[,1:2],RS=as.numeric(predict(fit,x,n.trees = best,type = 'link')))})

rs[["Train"]]=rs[["Train"]][rs[["Train"]]$RS !="Inf",]

rs[["Test"]]=rs[["Test"]][rs[["Test"]]$RS!="Inf",]

rs[["Train"]]=rs[["Train"]][rs[["Train"]]$RS !="-Inf",]

rs[["Test"]]=rs[["Test"]][rs[["Test"]]$RS!="-Inf",]

cc <- data.frame(Cindex=sapply(rs,function(x){as.numeric(summary(coxph(Surv(OS.time,OS)~RS,x))$concordance[1])}))%>%

rownames_to_column('ID')

cc$Model <- paste0('StepCox','[',direction,']',' + GBM')

result <- rbind(result,cc)

}

#######################################################################

#### 3-8.StepCox+survival-SVM ##########

#######################################################################

for (direction in c("both", "backward")) {

#direction='both'

fit <- step(coxph(Surv(OS.time,OS)~.,Train),direction = direction)

rid <- names(coef(fit))

train2 <- Train[,c('OS.time','OS',rid)]

trainlist2 <- lapply(trainlist,function(x){x[,c('OS.time','OS',rid)]})

fit = survivalsvm(Surv(OS.time,OS)~., data= train2, gamma.mu = 1)

rs <- lapply(trainlist2,function(x){cbind(x[,1:2],RS=as.numeric(predict(fit, x)$predicted))})

rs[["Train"]]=rs[["Train"]][rs[["Train"]]$RS !="Inf",]

rs[["Test"]]=rs[["Test"]][rs[["Test"]]$RS!="Inf",]

rs[["Train"]]=rs[["Train"]][rs[["Train"]]$RS !="-Inf",]

rs[["Test"]]=rs[["Test"]][rs[["Test"]]$RS!="-Inf",]

cc <- data.frame(Cindex=sapply(rs,function(x){as.numeric(summary(coxph(Surv(OS.time,OS)~RS,x))$concordance[1])}))%>%

rownames_to_column('ID')

cc$Model <- paste0('StepCox','[',direction,']',' + survival-SVM')

result <- rbind(result,cc)

}

################################################################

#### 3-9.StepCox+Ridge ##########

################################################################

for (direction in c("both", "backward")) {

fit <- step(coxph(Surv(OS.time,OS)~.,Train),direction = direction)

rid <- names(coef(fit))

train2 <- Train[,c('OS.time','OS',rid)]

trainlist2 <- lapply(trainlist,function(x){x[,c('OS.time','OS',rid)]})

set.seed(seed)

modelexp=as.matrix(train2[,c(3:ncol(train2))])

#利用循环探索Ridge最佳模型

for (alpha in seq(0,1,0.1)) {

set.seed(seed)

model <- glmnet(modelexp,train2$OS,family = 'binomial',alpha = alpha,nfolds=10)

model_cv<-cv.glmnet(modelexp,train2$OS,family = 'binomial',alpha =alpha,nfolds=10)

fit<-glmnet(modelexp,train2$OS,family = 'binomial',alpha = alpha,nfolds=10,keep=T,lambda = model_cv$lambda.min)

rs <- lapply(trainlist2,function(x){cbind(x[,1:2],RS=as.numeric(predict(fit,type="response",newx=as.matrix(x[,-c(1,2)]))))})

rs[["Train"]]=rs[["Train"]][rs[["Train"]]$RS !="Inf",]

rs[["Test"]]=rs[["Test"]][rs[["Test"]]$RS!="Inf",]

rs[["Train"]]=rs[["Train"]][rs[["Train"]]$RS !="-Inf",]

rs[["Test"]]=rs[["Test"]][rs[["Test"]]$RS!="-Inf",]

cc <- data.frame(Cindex=sapply(rs,function(x){as.numeric(summary(coxph(Surv(OS.time,OS)~RS,x))$concordance[1])}))%>%

rownames_to_column('ID')

cc$Model <- paste0('StepCox','[',direction,']','+ Ridge','[α=',alpha,']')

result <- rbind(result,cc)

}

}

# #######################################################################

# #### 3-10.StepCox+obliqueRSF ###########

# #######################################################################

# for (direction in c("both", "backward")) {

# fit <- step(coxph(Surv(OS.time,OS)~.,Train),direction = direction)

# rid <- names(coef(fit))

# train2 <- Train[,c('OS.time','OS',rid)]

# trainlist2 <- lapply(trainlist,function(x){x[,c('OS.time','OS',rid)]})

# model<-orsf(data = train2,n_tree = 100,formula = Surv(OS.time,OS)~.)

# rs <- lapply(trainlist2,function(x){cbind(x[,1:2],RS=as.numeric(predict(model, new_data=x,pred_type = "risk")[,1]))})

# rs[["Train"]]=rs[["Train"]][rs[["Train"]]$RS !="Inf",]

# rs[["Test"]]=rs[["Test"]][rs[["Test"]]$RS!="Inf",]

# rs[["Train"]]=rs[["Train"]][rs[["Train"]]$RS !="-Inf",]

# rs[["Test"]]=rs[["Test"]][rs[["Test"]]$RS!="-Inf",]

# cc <- data.frame(Cindex=sapply(rs,function(x){as.numeric(summary(coxph(Surv(OS.time,OS)~RS,x))$concordance[1])}))%>%

# rownames_to_column('ID')

# cc$Model <- paste0('StepCox','[',direction,']', '+ obliqueRSF')

# result <- rbind(result,cc)

# }

###################################################################

#### 3-11.StepCox+xgboost ##########

###################################################################

for (direction in c("both", "backward")) {

#direction='both'

fit <- step(coxph(Surv(OS.time,OS)~.,Train),direction = direction)

rid <- names(coef(fit))

train2 <- Train[,c('OS.time','OS',rid)]

trainlist2 <- lapply(trainlist,function(x){x[,c('OS.time','OS',rid)]})

#建立专用矩阵

model_mat<-xgb.DMatrix(data = as.matrix(train2[,-c(1:2)]),label=train2$OS.time)

#构建参数

object<-list(bojective="surivival:cox",

booster="gbtree",

eval_metric="cox-nloglik",

eta=0.01,

max_depth=3,

subsample=1,

colsample_bytree=1,

gamma=0.5)

#构建模型

model<-xgb.train(params=object,data = model_mat,nrounds = 100,watchlist = list(val2=model_mat),early_stopping_rounds = 10)

rs <- lapply(trainlist2,function(x){cbind(x[,1:2],RS=as.numeric(predict(model, newdata=as.matrix(x[,-c(1:2)]))))})

rs[["Train"]]=rs[["Train"]][rs[["Train"]]$RS !="Inf",]

rs[["Test"]]=rs[["Test"]][rs[["Test"]]$RS!="Inf",]

rs[["Train"]]=rs[["Train"]][rs[["Train"]]$RS !="-Inf",]

rs[["Test"]]=rs[["Test"]][rs[["Test"]]$RS!="-Inf",]

cc <- data.frame(Cindex=sapply(rs,function(x){as.numeric(summary(coxph(Surv(OS.time,OS)~RS,x))$concordance[1])}))%>%

rownames_to_column('ID')

cc$Model <- paste0('StepCox','[',direction,']', '+ xgboost')

result <- rbind(result,cc)

}

###################################################################

#### 3-12.StepCox+CForest ##########

###################################################################

for (direction in c("both", "backward")) {

fit <- step(coxph(Surv(OS.time,OS)~.,Train),direction = direction)

rid <- names(coef(fit))

train2 <- Train[,c('OS.time','OS',rid)]

trainlist2 <- lapply(trainlist,function(x){x[,c('OS.time','OS',rid)]})

model<-party::cforest(Surv(OS.time,OS)~.,data=train2,controls = cforest_unbiased(ntree=50))

rs <- lapply(trainlist2,function(x){cbind(x[,1:2],RS=as.numeric(predict(model, newdata=x,type = "response")))})

rs[["Train"]]=rs[["Train"]][rs[["Train"]]$RS !="Inf",]

rs[["Test"]]=rs[["Test"]][rs[["Test"]]$RS!="Inf",]

rs[["Train"]]=rs[["Train"]][rs[["Train"]]$RS !="-Inf",]

rs[["Test"]]=rs[["Test"]][rs[["Test"]]$RS!="-Inf",]

cc <- data.frame(Cindex=sapply(rs,function(x){as.numeric(summary(coxph(Surv(OS.time,OS)~RS,x))$concordance[1])}))%>%

rownames_to_column('ID')

cc$Model <- paste0('StepCox','[',direction,']',' + CForest')

result <- rbind(result,cc)

}

#################################################################

#### 3-13.StepCox+CTree ##########

#################################################################

for (direction in c("both", "backward")) {

fit <- step(coxph(Surv(OS.time,OS)~.,Train),direction = direction)

rid <- names(coef(fit))

train2 <- Train[,c('OS.time','OS',rid)]

trainlist2 <- lapply(trainlist,function(x){x[,c('OS.time','OS',rid)]})

model<-ctree(Surv(OS.time,OS)~.,data=train2)

rs <- lapply(trainlist2,function(x){cbind(x[,1:2],RS=as.numeric(predict(model, newdata=x,type = "response")))})

rs[["Train"]]=rs[["Train"]][rs[["Train"]]$RS !="Inf",]

rs[["Test"]]=rs[["Test"]][rs[["Test"]]$RS!="Inf",]

rs[["Train"]]=rs[["Train"]][rs[["Train"]]$RS !="-Inf",]

rs[["Test"]]=rs[["Test"]][rs[["Test"]]$RS!="-Inf",]

cc <- data.frame(Cindex=sapply(rs,function(x){as.numeric(summary(coxph(Surv(OS.time,OS)~RS,x))$concordance[1])}))%>%

rownames_to_column('ID')

cc$Model <- paste0('StepCox','[',direction,']',' + CTree')

result <- rbind(result,cc)

}

############################################################

#### 4-1.CoxBoost ###########

############################################################

set.seed(seed)

pen <- optimCoxBoostPenalty(Train[,'OS.time'],Train[,'OS'],as.matrix(Train[,-c(1,2)]), # 要改

trace=TRUE,start.penalty=500,parallel = T)

cv.res <- cv.CoxBoost(Train[,'OS.time'],Train[,'OS'],as.matrix(Train[,-c(1,2)]),

maxstepno=500,K=10,type="verweij",penalty=pen$penalty)

fit <- CoxBoost(Train[,'OS.time'],Train[,'OS'],as.matrix(Train[,-c(1,2)]),

stepno=cv.res$optimal.step,penalty=pen$penalty)

rs <- lapply(trainlist,function(x){cbind(x[,1:2],RS=as.numeric(predict(fit,newdata=x[,-c(1,2)], newtime=x[,1], newstatus=x[,2], type="lp")))})

rs[["Train"]]=rs[["Train"]][rs[["Train"]]$RS !="Inf",]

rs[["Test"]]=rs[["Test"]][rs[["Test"]]$RS!="Inf",]

rs[["Train"]]=rs[["Train"]][rs[["Train"]]$RS !="-Inf",]

rs[["Test"]]=rs[["Test"]][rs[["Test"]]$RS!="-Inf",]

cc <- data.frame(Cindex=sapply(rs,function(x){as.numeric(summary(coxph(Surv(OS.time,OS)~RS,x))$concordance[1])}))%>%

rownames_to_column('ID')

cc$Model <- paste0('CoxBoost')

result <- rbind(result,cc)

###############################################################

#### 4-2.CoxBoost+Enet #########

###############################################################

rid <- names(coef(fit)[which(coef(fit)!=0)])

train2 <- Train[,c('OS.time','OS',rid)] # 要改

trainlist2 <- lapply(trainlist,function(x){x[,c('OS.time','OS',rid)]})

x1 <- as.matrix(train2[,rid])

x2 <- as.matrix(Surv(train2$OS.time,train2$OS))

for (alpha in seq(0,1,0.1)) {

set.seed(seed)

fit = cv.glmnet(x1, x2,family = "cox",alpha=alpha,nfolds = 10)

rs <- lapply(trainlist2,function(x){cbind(x[,1:2],RS=as.numeric(predict(fit,type='link',newx=as.matrix(x[,-c(1,2)]),s=fit$lambda.min)))})

rs[["Train"]]=rs[["Train"]][rs[["Train"]]$RS !="Inf",]

rs[["Test"]]=rs[["Test"]][rs[["Test"]]$RS!="Inf",]

rs[["Train"]]=rs[["Train"]][rs[["Train"]]$RS !="-Inf",]

rs[["Test"]]=rs[["Test"]][rs[["Test"]]$RS!="-Inf",]

cc <- data.frame(Cindex=sapply(rs,function(x){as.numeric(summary(coxph(Surv(OS.time,OS)~RS,x))$concordance[1])}))%>%

rownames_to_column('ID')

cc$Model <- paste0('CoxBoost + Enet','[α=',alpha,']')

result <- rbind(result,cc)

}

##################################################################

#### 4-3.CoxBoost+stepcox #########

##################################################################

for (direction in c("both", "backward", "forward")) {

fit <- step(coxph(Surv(OS.time,OS)~.,train2),direction = direction)

rs <- lapply(trainlist2,function(x){cbind(x[,1:2],RS=predict(fit,type = 'risk',newdata = x))})

rs[["Train"]]=rs[["Train"]][rs[["Train"]]$RS !="Inf",]

rs[["Test"]]=rs[["Test"]][rs[["Test"]]$RS!="Inf",]

rs[["Train"]]=rs[["Train"]][rs[["Train"]]$RS !="-Inf",]

rs[["Test"]]=rs[["Test"]][rs[["Test"]]$RS!="-Inf",]

cc <- data.frame(Cindex=sapply(rs,function(x){as.numeric(summary(coxph(Surv(OS.time,OS)~RS,x))$concordance[1])}))%>%

rownames_to_column('ID')

cc$Model <- paste0('CoxBoost + StepCox','[',direction,']')

result <- rbind(result,cc)

}

##############################################################

#### 4-4.CoxBoost+RSF #########

##############################################################

set.seed(seed)

fit <- rfsrc(Surv(OS.time,OS)~.,data = train2,

ntree = 1000,nodesize = rf_nodesize,##该值建议多调整

splitrule = 'logrank',

importance = T,

proximity = T,

forest = T,

seed = seed)

best <- which.min(fit$err.rate)

set.seed(seed)

fit <- rfsrc(Surv(OS.time,OS)~.,data = train2,

ntree = best,nodesize = rf_nodesize,##该值建议多调整

splitrule = 'logrank',

importance = T,

proximity = T,

forest = T,

seed = seed)

rs <- lapply(trainlist2,function(x){cbind(x[,1:2],RS=predict(fit,newdata = x)$predicted)})

rs[["Train"]]=rs[["Train"]][rs[["Train"]]$RS !="Inf",]

rs[["Test"]]=rs[["Test"]][rs[["Test"]]$RS!="Inf",]

rs[["Train"]]=rs[["Train"]][rs[["Train"]]$RS !="-Inf",]

rs[["Test"]]=rs[["Test"]][rs[["Test"]]$RS!="-Inf",]

cc <- data.frame(Cindex=sapply(rs,function(x){as.numeric(summary(coxph(Surv(OS.time,OS)~RS,x))$concordance[1])}))%>%

rownames_to_column('ID')

cc$Model <- 'CoxBoost + RSF'

result <- rbind(result,cc)

#############################################################

#### 4-5.rsf+plsRcox #########

#############################################################

set.seed(seed)

cv.plsRcox.res=cv.plsRcox(list(x=train2[,rid],time=train2$OS.time,status=train2$OS),nt=10,nfold = 10,verbose = F)

fit <- plsRcox(train2[,rid],time=train2$OS.time,event=train2$OS,nt=as.numeric(cv.plsRcox.res[5]))

rs <- lapply(trainlist2,function(x){cbind(x[,1:2],RS=as.numeric(predict(fit,type="lp",newdata=x[,-c(1,2)])))})

rs[["Train"]]=rs[["Train"]][rs[["Train"]]$RS !="Inf",]

rs[["Test"]]=rs[["Test"]][rs[["Test"]]$RS!="Inf",]

rs[["Train"]]=rs[["Train"]][rs[["Train"]]$RS !="-Inf",]

rs[["Test"]]=rs[["Test"]][rs[["Test"]]$RS!="-Inf",]

cc <- data.frame(Cindex=sapply(rs,function(x){as.numeric(summary(coxph(Surv(OS.time,OS)~RS,x))$concordance[1])}))%>%

rownames_to_column('ID')

cc$Model <- paste0('CoxBoost + plsRcox')

result <- rbind(result,cc)

##################################################################

#### 4-6.CoxBoost+superpc #########

##################################################################

data <- list(x=t(train2[,-c(1,2)]),y=train2$OS.time,censoring.status=train2$OS,featurenames=colnames(train2)[-c(1,2)])

set.seed(seed)

fit <- superpc.train(data = data,type = 'survival',s0.perc = 0.5) #default

cv.fit <- superpc.cv(fit,data,n.threshold = 20,

n.fold = 5,

n.components=3,

min.features=1,

max.features=nrow(data$x),

compute.fullcv= TRUE,

compute.preval=TRUE)

rs <- lapply(trainlist2,function(w){

test <- list(x=t(w[,-c(1,2)]),y=w$OS.time,censoring.status=w$OS,featurenames=colnames(w)[-c(1,2)])

ff <- superpc.predict(fit,data,test,threshold = cv.fit$thresholds[which.max(cv.fit[["scor"]][1,])],n.components = 1)

rr <- as.numeric(ff$v.pred)

rr2 <- cbind(w[,1:2],RS=rr)

return(rr2)

})

rs[["Train"]]=rs[["Train"]][rs[["Train"]]$RS !="Inf",]

rs[["Test"]]=rs[["Test"]][rs[["Test"]]$RS!="Inf",]

rs[["Train"]]=rs[["Train"]][rs[["Train"]]$RS !="-Inf",]

rs[["Test"]]=rs[["Test"]][rs[["Test"]]$RS!="-Inf",]

cc <- data.frame(Cindex=sapply(rs,function(x){as.numeric(summary(coxph(Surv(OS.time,OS)~RS,x))$concordance[1])}))%>%

rownames_to_column('ID')

cc$Model <- paste0('CoxBoost + SuperPC')

result <- rbind(result,cc)

##############################################################

#### 4-7.CoxBoost+gbm #########

##############################################################

set.seed(seed)

fit <- gbm(formula = Surv(OS.time,OS)~.,data = train2,distribution = 'coxph',

n.trees = 1000,

interaction.depth = 3,

n.minobsinnode = 10,

shrinkage = 0.001,

cv.folds = 3,n.cores = 1)

best <- which.min(fit$cv.error)

set.seed(seed)

fit <- gbm(formula = Surv(OS.time,OS)~.,data = train2,distribution = 'coxph',

n.trees = best,

interaction.depth = 3,

n.minobsinnode = 10,

shrinkage = 0.001,

cv.folds = 3,n.cores = 1)

rs <- lapply(trainlist2,function(x){cbind(x[,1:2],RS=as.numeric(predict(fit,x,n.trees = best,type = 'link')))})

rs[["Train"]]=rs[["Train"]][rs[["Train"]]$RS !="Inf",]

rs[["Test"]]=rs[["Test"]][rs[["Test"]]$RS!="Inf",]

rs[["Train"]]=rs[["Train"]][rs[["Train"]]$RS !="-Inf",]

rs[["Test"]]=rs[["Test"]][rs[["Test"]]$RS!="-Inf",]

cc <- data.frame(Cindex=sapply(rs,function(x){as.numeric(summary(coxph(Surv(OS.time,OS)~RS,x))$concordance[1])}))%>%

rownames_to_column('ID')

cc$Model <- paste0('CoxBoost + GBM')

result <- rbind(result,cc)

######################################################################

#### 4-8.CoxBoost+survivalsvm #########

######################################################################

fit = survivalsvm(Surv(OS.time,OS)~., data= train2, gamma.mu = 2)

rs <- lapply(trainlist2,function(x){cbind(x[,1:2],RS=as.numeric(predict(fit, x)$predicted))})

rs[["Train"]]=rs[["Train"]][rs[["Train"]]$RS !="Inf",]

rs[["Test"]]=rs[["Test"]][rs[["Test"]]$RS!="Inf",]

rs[["Train"]]=rs[["Train"]][rs[["Train"]]$RS !="-Inf",]

rs[["Test"]]=rs[["Test"]][rs[["Test"]]$RS!="-Inf",]

cc <- data.frame(Cindex=sapply(rs,function(x){as.numeric(summary(coxph(Surv(OS.time,OS)~RS,x))$concordance[1])}))%>%

rownames_to_column('ID')

cc$Model <- paste0('CoxBoost + survival-SVM')

result <- rbind(result,cc)

######################################################

#### 5.plsRcox#########

######################################################

set.seed(seed)

cv.plsRcox.res=cv.plsRcox(list(x=Train[,-c(1,2)],time=Train$OS.time,status=Train$OS),nt=10,nfold = 10,verbose = F) # 要改

fit <- plsRcox(Train[,-c(1,2)],time=Train$OS.time,event=Train$OS,nt=as.numeric(cv.plsRcox.res[5]))

rs <- lapply(trainlist,function(x){cbind(x[,1:2],RS=as.numeric(predict(fit,type="lp",newdata=x[,-c(1,2)])))})

rs[["Train"]]=rs[["Train"]][rs[["Train"]]$RS !="Inf",]

rs[["Test"]]=rs[["Test"]][rs[["Test"]]$RS!="Inf",]

rs[["Train"]]=rs[["Train"]][rs[["Train"]]$RS !="-Inf",]

rs[["Test"]]=rs[["Test"]][rs[["Test"]]$RS!="-Inf",]

cc <- data.frame(Cindex=sapply(rs,function(x){as.numeric(summary(coxph(Surv(OS.time,OS)~RS,x))$concordance[1])}))%>%

rownames_to_column('ID')

cc$Model <- paste0('plsRcox')

result <- rbind(result,cc)

######################################################

#### 6.superpc#########

######################################################

data <- list(x=t(Train[,-c(1,2)]),y=Train$OS.time,censoring.status=Train$OS,featurenames=colnames(Train)[-c(1,2)]) # 要改

set.seed(seed)

fit <- superpc.train(data = data,type = 'survival',s0.perc = 0.5) #default

cv.fit <- superpc.cv(fit,data,n.threshold = 20,#default

n.fold = 10,

n.components=3,

min.features=1,

max.features=nrow(data$x),

compute.fullcv= TRUE,

compute.preval=TRUE)

rs <- lapply(trainlist,function(w){

test <- list(x=t(w[,-c(1,2)]),y=w$OS.time,censoring.status=w$OS,featurenames=colnames(w)[-c(1,2)])

ff <- superpc.predict(fit,data,test,threshold = cv.fit$thresholds[which.max(cv.fit[["scor"]][1,])],n.components = 1)

rr <- as.numeric(ff$v.pred)

rr2 <- cbind(w[,1:2],RS=rr)

return(rr2)

})

rs[["Train"]]=rs[["Train"]][rs[["Train"]]$RS !="Inf",]

rs[["Test"]]=rs[["Test"]][rs[["Test"]]$RS!="Inf",]

rs[["Train"]]=rs[["Train"]][rs[["Train"]]$RS !="-Inf",]

rs[["Test"]]=rs[["Test"]][rs[["Test"]]$RS!="-Inf",]

cc <- data.frame(Cindex=sapply(rs,function(x){as.numeric(summary(coxph(Surv(OS.time,OS)~RS,x))$concordance[1])}))%>%

rownames_to_column('ID')

cc$Model <- paste0('SuperPC')

result <- rbind(result,cc)

###################################################

#### 7.GBM #########

###################################################

set.seed(seed)

fit <- gbm(formula = Surv(OS.time,OS)~.,data = Train,distribution = 'coxph', # 要改

n.trees = 1000,

interaction.depth = 3,

n.minobsinnode = 10,

shrinkage = 0.001,

cv.folds = 5,n.cores = 1)

# find index for number trees with minimum CV error

best <- which.min(fit$cv.error)

set.seed(seed)

fit <- gbm(formula = Surv(OS.time,OS)~.,data = Train,distribution = 'coxph',

n.trees = best,

interaction.depth = 3,

n.minobsinnode = 10,

shrinkage = 0.001,

cv.folds = 5,n.cores = 1)

rs <- lapply(trainlist,function(x){cbind(x[,1:2],RS=as.numeric(predict(fit,x,n.trees = best,type = 'link')))})

rs[["Train"]]=rs[["Train"]][rs[["Train"]]$RS !="Inf",]

rs[["Test"]]=rs[["Test"]][rs[["Test"]]$RS!="Inf",]

rs[["Train"]]=rs[["Train"]][rs[["Train"]]$RS !="-Inf",]

rs[["Test"]]=rs[["Test"]][rs[["Test"]]$RS!="-Inf",]

cc <- data.frame(Cindex=sapply(rs,function(x){as.numeric(summary(coxph(Surv(OS.time,OS)~RS,x))$concordance[1])}))%>%

rownames_to_column('ID')

cc$Model <- paste0('GBM')

result <- rbind(result,cc)

###########################################################

#### 8.survivalsvm #########

###########################################################

fit = survivalsvm(Surv(OS.time,OS)~., data= Train, gamma.mu = 2) # 要改

rs <- lapply(trainlist,function(x){cbind(x[,1:2],RS=as.numeric(predict(fit, x)$predicted))})

rs[["Train"]]=rs[["Train"]][rs[["Train"]]$RS !="Inf",]

rs[["Test"]]=rs[["Test"]][rs[["Test"]]$RS!="Inf",]

rs[["Train"]]=rs[["Train"]][rs[["Train"]]$RS !="-Inf",]

rs[["Test"]]=rs[["Test"]][rs[["Test"]]$RS!="-Inf",]

cc <- data.frame(Cindex=sapply(rs,function(x){as.numeric(summary(coxph(Surv(OS.time,OS)~RS,x))$concordance[1])}))%>%

rownames_to_column('ID')

cc$Model <- paste0('survival-SVM')

result <- rbind(result,cc)

#

save(result,file = "../101机器学习/不用封装的跑/最终版_用于画图的result_24_9_11.Rdata")

##

rf_nodesize <- 5

seed <- 123

#

set.seed(seed)

#

Train[1:4,1:5]

# OS.time OS BGN GADD45B JUN

# GSM1419944 1066 1 10.40476 8.875197 11.23499

# GSM1419946 422 1 11.61945 10.102057 10.67574

# GSM1419948 4517 0 10.01371 9.349203 10.28792

# GSM1419949 3070 0 11.24955 10.199535 11.24470

training=Train

#

set.seed(seed)

# 1，确定最优penalty

# 使用optimCoxBoostPenalty函数筛选当前数据的最优penalty ，将得到的pen$penalty 定为最终模型的参数

pen <- optimCoxBoostPenalty(training[,'OS.time'],

training[,'OS'],

as.matrix(training[,-c(1,2)]),

trace=TRUE,

start.penalty=500,

parallel = T)

pen$penalty

#[1] 5400

# 2，确定最优stepno

# 使用cv.CoxBoost函数确定最优stepno，取cv.res$optimal.step的值

#number of folds to be used for cross-validation

cv.res <- cv.CoxBoost(training[,'OS.time'],

training[,'OS'],

as.matrix(training[,-c(1,2)]),

maxstepno=500,

K=10,

type="verweij",

penalty= pen$penalty

# ,multicore=1

)

cv.res$optimal.step

#[1] 86

# 构建模型

# 使用上面得到的参数构建CoxBoost模型

fit <- CoxBoost(training[,'OS.time'],

training[,'OS'],

as.matrix(training[,-c(1,2)]),

stepno=cv.res$optimal.step,

penalty=pen$penalty)

summary(fit)

# 228 boosting steps resulting in 5 non-zero coefficients

# partial log-likelihood: -70.00765

#

# parameter estimates > 0:

# BGN, JUN, NOTCH3, PMEPA1, UQCRQ

# parameter estimates < 0:

plot(fit)

selected_vars_cb <- names(coef(fit))[coef(fit) != 0]

# 查看选择的变量

print(selected_vars_cb)

data=training

# 运行Enet[0]

data_coxboost <- data %>% dplyr::select(c("OS.time", "OS", selected_vars_cb))

#

x <- as.matrix(data_coxboost[,c(3:ncol(data_coxboost))]) # 预测变量矩阵（除去 OS.time 和 OS 列）

y <- Surv(data_coxboost$OS.time,data_coxboost$OS) # 生存对象

# 3. 使用 Elastic Net

alpha_value <- 0.5

# 使用 glmnet 进行模型拟合

enet_model <- glmnet(x, y, family = "cox", alpha = alpha_value,nfolds=10)

cv_enet <- cv.glmnet(x, y, family = "cox", alpha = alpha_value,nfolds=10)

cv_enet

best_lambda <- cv_enet$lambda.min

plot(cv_enet)

# coef_enet <- coef(enet_model, s = best_lambda)

# coef_enet=as.matrix(coef_enet)

# selected_vars_enet <- rownames(coef_enet)[coef_enet != 0]

# print(selected_vars_enet)

#此处使用lambda.min, 也可以尝试lambda.1se

coefficient <- coef(cv_enet, s = best_lambda)

#系数不等于0的为纳入的变量（基因）

Active.index <- which(as.numeric(coefficient) != 0)

Active.coefficient <- as.numeric(coefficient)[Active.index]

sig_gene_mult_cox <- rownames(coefficient)[Active.index]

#查看具体哪些基因

sig_gene_mult_cox

training_cox <- data_coxboost %>%

dplyr::select(OS,OS.time,sig_gene_mult_cox)

multiCox <- coxph(Surv(OS.time, OS) ~ ., data = training_cox)

coefficients <- coef(multiCox)

selected_vars_stepcox <- names(coefficients)

cat("Selected Variables after StepCox:", paste(selected_vars_stepcox, collapse = ", "), "\n")

print(coefficients)

#predict函数计算风险评分

riskScore=predict(multiCox,type="risk",newdata=training_cox)

riskScore<-as.data.frame(riskScore)

riskScore$sample <- rownames(riskScore)

head(riskScore,2)

#

riskScore_cli <- training_cox %>%

rownames_to_column("sample") %>%

inner_join(riskScore)

# 计算ROC

data=riskScore_cli

data$risk_score=data$riskScore

time_points <- c( 3, 5,7) * 365

roc_3 <- survivalROC(Stime = data$OS.time, status = data$OS, marker = data$risk_score, predict.time = time_points[1], method = "KM")

roc_5 <- survivalROC(Stime = data$OS.time, status = data$OS, marker = data$risk_score, predict.time = time_points[2], method = "KM")

roc_7 <- survivalROC(Stime = data$OS.time, status = data$OS, marker = data$risk_score, predict.time = time_points[3], method = "KM")

riskScore_cli$riskScore2 <- ifelse(riskScore_cli$riskScore > median(riskScore_cli$riskScore),

"High","Low")

#KM分析

fit2 <- survfit(Surv(OS.time, as.numeric(OS)) ~ riskScore2, data=riskScore_cli)

p2 <- ggsurvplot(fit2, data = riskScore_cli,

pval = T,

risk.table = T,

surv.median.line = "hv", #添加中位生存曲线

palette=c("red", "blue"), #更改线的颜色

legend.labs=c("High risk","Low risk"), #标签

legend.title="RiskScore",

title="Overall survival", #标题

ylab="Cumulative survival (percentage)",xlab = " Time (Days)", #更改横纵坐标

censor.shape = 124,censor.size = 2,conf.int = FALSE, #删失点的形状和大小 break.x.by = 720#横坐标间隔

)

p2

#

Train_riskScore_cli=riskScore_cli

# 验证集呢

traindataset_cox <- TrainDataset %>%

dplyr::select(OS,OS.time,sig_gene_mult_cox)

riskScore=predict(multiCox,type="risk",newdata=traindataset_cox)

riskScore<-as.data.frame(riskScore)

riskScore$sample <- rownames(riskScore)

head(riskScore,2)

#

riskScore_cli <- traindataset_cox %>%

rownames_to_column("sample") %>%

inner_join(riskScore)

data=riskScore_cli

data$risk_score=data$riskScore

time_points <- c(3, 5,7) * 365

roc_3 <- survivalROC(Stime = data$OS.time, status = data$OS, marker = data$risk_score, predict.time = time_points[1], method = "KM")

roc_5 <- survivalROC(Stime = data$OS.time, status = data$OS, marker = data$risk_score, predict.time = time_points[2], method = "KM")

roc_7 <- survivalROC(Stime = data$OS.time, status = data$OS, marker = data$risk_score, predict.time = time_points[3], method = "KM")

riskScore_cli$riskScore2 <- ifelse(riskScore_cli$riskScore > median(riskScore_cli$riskScore),

"High","Low")

#KM分析

fit2 <- survfit(Surv(OS.time, as.numeric(OS)) ~ riskScore2, data=riskScore_cli)

p2 <- ggsurvplot(fit2, data = riskScore_cli,

pval = T,

risk.table = T,

surv.median.line = "hv", #添加中位生存曲线

palette=c("red", "blue"), #更改线的颜色

legend.labs=c("High risk","Low risk"), #标签

legend.title="RiskScore",

title="Overall survival", #标题

ylab="Cumulative survival (percentage)",xlab = " Time (Days)", #更改横纵坐标

censor.shape = 124,censor.size = 2,conf.int = FALSE, #删失点的形状和大小 break.x.by = 720#横坐标间隔

)

p2

#

Fig6.R

####Fig5I####

{

##### 01-诺莫图 #####

#

# 需要修改, 全部改成因子类型

riskScore_cli2$Stage <- ifelse(riskScore_cli2$Stage %in% c("I","II"), "Low", "High")

riskScore_cli2$Tumor <- ifelse(riskScore_cli2$Tumor %in% c("T1","T4"), "High", "Low")

riskScore_cli2 <- Test_riskScore_cli_4

# 设置因子型变量

riskScore_cli2$Stage <- factor(riskScore_cli2$Stage, levels = c("High","Low" ))

levels(riskScore_cli2$Stage)

riskScore_cli2$Tumor <- factor(riskScore_cli2$Tumor, levels = c("High","Low" ))

levels(riskScore_cli2$Tumor)

riskScore_cli2$Metastasis <- factor(riskScore_cli2$Metastasis, levels = c("Yes","No"))

levels(riskScore_cli2$Metastasis)

riskScore_cli2$Weight <- factor(riskScore_cli2$Weight, levels = c("High","Low" ))

levels(riskScore_cli2$Weight)

riskScore_cli2$riskScore2 <- factor(riskScore_cli2$riskScore2, levels = c("High","Low" ))

levels(riskScore_cli2$riskScore2)

riskScore_cli2$Age <- factor(riskScore_cli2$Age, levels = c("High","Low" ))

levels(riskScore_cli2$Age)

colnames(riskScore_cli2)[c(5,6)] <- c( "OS", "OS.time")

riskScore_cli2$OS.time <- round(riskScore_cli2$OS.time/365, 2)

colnames(riskScore_cli2)

#

dd <- datadist(riskScore_cli2)

options(datadist="dd")

f <- psm(Surv(OS.time,OS) ~ Stage + Tumor + Weight + riskScore2 + Metastasis+age,

data = riskScore_cli2,dist='lognormal')

surv <- Survival(f) # 构建生存概率函数

## time是以”天“为单位,此处绘制1年，3,5年的生存概率

nom <- nomogram(f, fun=list(function(x) surv(1, x),

function(x) surv(3, x),

function(x) surv(5, x) ),

funlabel=c("1-year OS", "3-year OS",

"5-year OS"))

plot(nom, xfrac=.2)

# 接下来使用优美画法

library(regplot)

Cox_nomo2 <- cph(Surv(OS.time,OS) ~ Stage + Tumor + Weight + riskScore2 + Metastasis+age ,

data = riskScore_cli2,dist='lognormal', x=T, y=T)

regplot(Cox_nomo2,

# observation = riskScore_cli2[4,], #指定某一患者

interval ="confidence",

title="Nomogram",

plots=c("violin", "boxes"),

clickable = T,

failtime = c(1,3,5)) #设置随访时间1年、3年和5年

}

#### Fig5GH####

{

##### 02-COX曲线 #####

cox_need

library(tidyverse)

cox_need <- rownames_to_column(cox_need, var = "Variable")

cox_need$hazard_ratio <- paste0(cox_need$HR,"(",cox_need$ower_95,"-",cox_need$upper_95,")")

dat <- cox_need[,c(1,6,10,11,12,5)]

colnames(dat)=c("Variable","HR","CI5","CI95","HR (95% CI)","Pvalue")

dat = rbind(c("Names", NA,NA,NA,"Hazard Ratio(95% CI)", "p.value"),dat)

dat <- dat[c(1,7,6,4,5,3,2),]

dat$Variable <- c(NA,"Age","Weight","Stage","Tumor","Metastasis","RiskScore")

dat <- dat[,c(1:4,6,5)]

#画图

forestplot(dat[,c(1,5,6)], #显示表格的第1，5，6列内容

mean=dat[,2], #第2列为HR，变成森林图的方块

lower=dat[,3], upper=dat[,4], #第3列为5%CI，第4列为95%CI，将化作线段，穿过方块

zero=1, #零线或参考线位置为HR=1

boxsize=0.2, #设置方块大小

graph.pos=4,#将森林图插在第3列

xticks=c(-5,0,5,10,15) ,# 设置横轴数字

txt_gp=fpTxtGp (

label=gpar(cex=0.8) ,ticks=gpar(cex=0.6)

),#调整字体

# hrzl_lines=list("1" = gpar(lty=1,lwd=1.5),

# "2" = gpar(lty=1,lwd=1.5),

# "5"= gpar(lty=1,lwd=1.5)), # 在1,2,7行添加横线

col=fpColors ( box = 'red ' , #方块颜色

lines = ' black ' ,#置信区间横线颜色

zero = "grey" ),#参考线颜色

lwd.zero=1,#参考线宽度

lwd.ci=1.5, # 置信区间横线宽度

lty.ci=7 ,# 置信区间横线类型

ci.vertices.height=0.1, #置信区间横线两端竖线高度

title = "OS (Univariate Cox)"

)

# 单因素出完出多因素的

# coxph(Surv(futime, fustat) ~ Metastasis + RiskScore + Stage, data = Test_riskScore_cli_4)

colnames(Test_riskScore_cli_4)

res <- coxph(Surv(futime, fustat) ~ Metastasis + riskScore2 + Stage, data = Test_riskScore_cli_4)

summary(res)

mul_cox <- summary(res)

mul_HR<- round(mul_cox$coefficients[,2],2)

mul_Pvalue<- round(mul_cox$coefficients[,5],4)

mul_CI5<-round(mul_cox$conf.int[,3],2)

mul_CI95<-round(mul_cox$conf.int[,4],2)

mul_CI<-paste0(mul_HR,' (',mul_CI5,'-',mul_CI95,')')

Variable<-row.names(data.frame(mul_cox$coefficients))

mulcox_res<- data.frame(Variable,mul_HR,mul_CI5,mul_CI95,mul_CI,mul_Pvalue)

colnames(mulcox_res)=c("Variable","HR","CI5","CI95","HR (95% CI)","Pvalue")

View(mulcox_res)

library(forestplot)

#添加表头

dat=rbind(c("Variable", NA,NA,NA,"HR (95% CI)", "Pvalue"),mulcox_res)

dat <- dat[,c(1:4,6,5)]

dat[1,1] <- NA

dat[2,1] <- "Metastasis"

dat[3,1] <- "RiskScore"

dat[4,1] <- "Stage"

#画图

forestplot(dat[,c(1,5,6)], #显示表格的第1，5，6列内容

mean=dat[,2], #第2列为HR，变成森林图的方块

lower=dat[,3], upper=dat[,4], #第3列为5%CI，第4列为95%CI，将化作线段，穿过方块

zero=1, #零线或参考线位置为HR=1

boxsize=0.2, #设置方块大小

graph.pos=4,#将森林图插在第3列

xticks=c(-5,0,5,10,15) ,# 设置横轴数字

txt_gp=fpTxtGp (

label=gpar(cex=0.8) ,ticks=gpar(cex=0.6)

),#调整字体

# hrzl_lines=list("1" = gpar(lty=1,lwd=1.5),

# "2" = gpar(lty=1,lwd=1.5),

# "5"= gpar(lty=1,lwd=1.5)), # 在1,2,7行添加横线

col=fpColors ( box = 'red ' , #方块颜色

lines = ' black ' ,#置信区间横线颜色

zero = "grey" ),#参考线颜色

lwd.zero=1,#参考线宽度

lwd.ci=1.5, # 置信区间横线宽度

lty.ci=7 ,# 置信区间横线类型

ci.vertices.height=0.1, #置信区间横线两端竖线高度

title = "OS (Multivariate Cox)"

)

}

####Fig5J####

{

#####03-timeROC#####

install.packages('timeROC')

library(timeROC)

time_points <- c(1, 3, 5, 7) * 365.25 # 将年份转换为天数

data <- Test_riskScore_cli_4

data$OS.time <- data$futime

data$OS <- data$fustat

data$risk_score <- data$riskScore

# 计算 1 年、3 年、5 年的 ROC 曲线

roc_1 <- timeROC(T = data$OS.time, delta = data$OS, marker = data$risk_score, cause = 1, times = time_points[1])

roc_3 <- timeROC(T = data$OS.time, delta = data$OS, marker = data$risk_score, cause = 1, times = time_points[2])

roc_5 <- timeROC(T = data$OS.time, delta = data$OS, marker = data$risk_score, cause = 1, times = time_points[3])

roc_7 <- timeROC(T = data$OS.time, delta = data$OS, marker = data$risk_score, cause = 1, times = time_points[4])

roc_1$AUC;roc_3$AUC;roc_5$AUC;roc_7$AUC

data$fustat <- as.factor(data$fustat)

data$futime<- round(data$futime/365, 2)

## 构建timeROC

ROC <- timeROC(T=data$futime, #生存时间

delta=data$fustat, #生存状态

marker=data$riskScore, #计算timeROC的变量

cause=1, #阳性结局指标数值(1表示死亡)

weighting="marginal", #计算方法，默认为marginal

times=c(1, 3, 5, 7), #时间点，选取1年，3年和5年的生存率

iid=TRUE)

ROC

plot(ROC,

time=1, col="red", lty=1,lwd=2, title = "") #time是时间点，col是线条颜色、lty为图例线条类型、lwd为图例线条宽度

plot(ROC,

time=3, col="blue", add=TRUE, lty=1,lwd=2) #add指是否添加在上一张图中

plot(ROC,

time=5, col="orange", add=TRUE, lty=1,lwd=2)

plot(ROC,

time=7, col="yellow", add=TRUE, lty=1,lwd=2)

## 添加图例

legend("bottomright",#图例画在右下角

c(paste0("AUC at 1 year: ",round(ROC[["AUC_1"]][1],2)), #提取1年AUC构建图例标签

paste0("AUC at 3 year: ",round(ROC[["AUC_1"]][2],2)), #提取3年AUC构建图例标签

paste0("AUC at 5 year: ",round(ROC[["AUC_1"]][3],2)),#提取5年AUC构建图例标签

paste0("AUC at 7 year: ",round(ROC[["AUC_1"]][4],2))),#提取5年AUC构建图例标签

col=c("red",

"blue",

"orange",

"yellow"), #设置1，3，5年AUC图例标签的图例颜色，注意与曲线保持对应

lty=1,

lwd=2,

bty = "n" #o表示用框框把图例部分框起来，为默认。n表示不画框框

)

colnames(Test_riskScore_cli_4)

f <- coxph(Surv(futime, fustat)~riskScore + Stage + Metastasis, data = Test_riskScore_cli_4)

Test_riskScore_cli_4$riskscoreall <- predict(f, type ="lp")

data <- Test_riskScore_cli_4

data$fustat <- as.factor(data$fustat)

## 构建timeROC

ROC <- timeROC(T=data$futime, #生存时间

delta=data$fustat, #生存状态

marker=data$riskscoreall, #计算timeROC的变量

cause=1, #阳性结局指标数值(1表示死亡)

weighting="marginal", #计算方法，默认为marginal

times=c(1, 3, 5, 7)*365, #时间点，选取1年，3年和5年的生存率

iid=TRUE)

ROC

plot(ROC,

time=1*365, col="red", lty=1,lwd=2, title = "") #time是时间点，col是线条颜色、lty为图例线条类型、lwd为图例线条宽度

plot(ROC,

time=3*365, col="blue", add=TRUE, lty=1,lwd=2) #add指是否添加在上一张图中

plot(ROC,

time=5*365, col="orange", add=TRUE, lty=1,lwd=2)

plot(ROC,

time=7*365, col="yellow", add=TRUE, lty=1,lwd=2)

## 添加图例

legend("bottomright",#图例画在右下角

c(paste0("AUC at 1 year: ",round(ROC[["AUC_1"]][1],2)), #提取1年AUC构建图例标签

paste0("AUC at 3 year: ",round(ROC[["AUC_1"]][2],2)), #提取3年AUC构建图例标签

paste0("AUC at 5 year: ",round(ROC[["AUC_1"]][3],2)),#提取5年AUC构建图例标签

paste0("AUC at 7 year: ",round(ROC[["AUC_1"]][4],2))),#提取5年AUC构建图例标签

col=c("red",

"blue",

"orange",

"yellow"), #设置1，3，5年AUC图例标签的图例颜色，注意与曲线保持对应

lty=1,

lwd=2,

bty = "n" #o表示用框框把图例部分框起来，为默认。n表示不画框框

)

library(survival)

data$facriskscoreall <- ifelse(data$riskscoreall>=median(data$riskscoreall), "High", "Low")

data$facriskscoreall <- factor(data$facriskscoreall, levels = c("Low", "High"))

# cox <- coxph(Surv(futime,fustat)~riskScore2 + Metastasis + Stage, data = data)

# summary(cox)

cox <- coxph(Surv(futime, fustat)~riskScore2 + Stage + Metastasis, data = Test_riskScore_cli_4)

summary(cox)

# 然后基于riskscore和临床指标预测临床风险评分：

data$riskscore3 <- predict(cox, type = "lp")

head(data)

# riskscore2 OS.time OS riskscore3

# 1 1.634682 967 1 1.634682

# 2 1.634682 584 0 1.634682

# 3 1.634682 2654 0 1.634682

# 4 0.000000 754 1 0.000000

# 5 0.000000 2048 0 0.000000

# 6 1.634682 1027 0 1.634682

# 然后进行ROC分析：

#

# data$OS.time <- data$futime

# data$OS <- data$fustat

library(survivalROC)

ROC1<- survivalROC(Stime=data$OS.time,

status=data$OS,

marker = data$riskscore3,

predict.time =365*1, #1年生存率,也可改为3年生存率 365*3

method = "KM")

ROC1

ROC2<- survivalROC(Stime=data$OS.time,

status=data$OS,

marker = data$riskscore3,

predict.time =365*3, #1年生存率,也可改为3年生存率 365*3

method = "KM")

ROC2

###############画ROC3

ROC3<- survivalROC(Stime=data$OS.time,

status=data$OS,

marker = data$riskscore3,

predict.time =365*5, #1年生存率,也可改为3年生存率 365*3

method = "KM")

ROC3

#

ROC4<- survivalROC(Stime=data$OS.time,

status=data$OS,

marker = data$riskscore3,

predict.time =365*7, #1年生存率,也可改为3年生存率 365*3

method = "KM")

ROC4

# 画图所有代码在这里

{

plot(ROC1$FP, ROC1$TP, type="l", xlim=c(0,1), ylim=c(0,1),col="red",

xlab="False positive rate", ylab="True positive rate",

lwd = 2, cex.main=1, cex.lab=1, cex.axis=1.2, font=1.2)

abline(0,1)

aucText=paste0("1 years"," (AUC=",sprintf("%.3f",ROC1$AUC),")") #这个后面添加legend用

lines(ROC2$FP, ROC2$TP, type="l", xlim=c(0,1), ylim=c(0,1),col="orange",lwd = 2)

aucText3=paste0("3 years"," (AUC=",sprintf("%.3f",ROC2$AUC),")") #这个后面添加legend用

lines(ROC3$FP, ROC3$TP, type="l", xlim=c(0,1), ylim=c(0,1),col="cyan",lwd = 2)

aucText5=paste0("5 years"," (AUC=",sprintf("%.3f",ROC3$AUC),")") #这个后面添加legend用

lines(ROC4$FP, ROC4$TP, type="l", xlim=c(0,1), ylim=c(0,1),col="yellow",lwd = 2)

aucText7=paste0("7 years"," (AUC=",sprintf("%.3f",ROC4$AUC),")") #这个后面添加legend用

#添加legend

legend("bottomright", c(aucText,aucText3,aucText5,aucText7),

lwd=2,bty="n",col=c("red","orange","cyan","yellow"),cex=0.8)

}

ROC1$AUC;ROC2$AUC;ROC3$AUC;ROC4$AUC

plot(ROC1,

time=1*365, col="red", lty=1,lwd=2, title = "") #time是时间点，col是线条颜色、lty为图例线条类型、lwd为图例线条宽度

plot(ROC2,

time=3*365, col="blue", add=TRUE, lty=1,lwd=2) #add指是否添加在上一张图中

plot(ROC3,

time=5*365, col="orange", add=TRUE, lty=1,lwd=2)

plot(ROC4,

time=7*365, col="yellow", add=TRUE, lty=1,lwd=2)

## 添加图例

legend("bottomright",#图例画在右下角

c(paste0("AUC at 1 year: ",round(ROC[["AUC_1"]][1],2)), #提取1年AUC构建图例标签

paste0("AUC at 3 year: ",round(ROC[["AUC_1"]][2],2)), #提取3年AUC构建图例标签

paste0("AUC at 5 year: ",round(ROC[["AUC_1"]][3],2)),#提取5年AUC构建图例标签

paste0("AUC at 7 year: ",round(ROC[["AUC_1"]][4],2))),#提取5年AUC构建图例标签

col=c("red",

"blue",

"orange",

"yellow"), #设置1，3，5年AUC图例标签的图例颜色，注意与曲线保持对应

lty=1,

lwd=2,

bty = "n" #o表示用框框把图例部分框起来，为默认。n表示不画框框

)

}

####Fig5K####

{

#####04-C-index#####

# C-index

library(rms)

library(pec)

library(ggplot2)

riskScore_cli2$OS.time <- riskScore_cli2$OS.time2

###

models=list( Stage=cph(Surv(OS.time,OS)~Stage,data=riskScore_cli2,x=TRUE,y=TRUE,surv=T),

Tumor=cph(Surv(OS.time,OS)~Tumor,data=riskScore_cli2,x=TRUE,y=TRUE,surv=T),

Weight=cph(Surv(OS.time,OS)~Weight,data=riskScore_cli2,x=TRUE,y=TRUE,surv=T),

riskScore2=cph(Surv(OS.time,OS)~riskScore2,data=riskScore_cli2,x=TRUE,y=TRUE,surv=T),

Metastasis=cph(Surv(OS.time,OS)~Metastasis,data=riskScore_cli2,x=TRUE,y=TRUE,surv=T),

Age=cph(Surv(OS.time,OS)~Age,data=riskScore_cli2,x=TRUE,y=TRUE,surv=T),

riskscoreall=cph(Surv(OS.time,OS)~riskscoreall,data=riskScore_cli2,x=TRUE,y=TRUE,surv=T))

#time-cindex计算

times<-c(1,3,5,7)

cindex<-cindex(models,

formula=Surv(OS.time,OS)~1,

eval.times=times,

data=riskScore_cli2)

plot(cindex)

cindex$AppCindex

cindex_df<-data.frame(

Time=times,

do.call(cbind,cindex$AppCindex)

)

cindex_df

library(tidyr)

dat=pivot_longer(cindex_df,cols=2:7,

names_to="model",

values_to="cindex")

head(dat)

library(ggplot2)

ggplot(dat,aes(x=Time,y=cindex))+

geom_line(aes(color=model),linewidth=2)+

scale_color_brewer(palette="Set1")+

geom_hline(yintercept=0.5,linetype=4)+

ylim(0.4,1)+

labs(title="Time-dependentC-index",x="Time(years)",y="C-index")+

theme_bw()

library(survival)

data <- riskScore_cli2

cox <- coxph(Surv(OS.time,OS)~as.factor(riskScore2) + as.factor(Stage),data)

summary(cox)

# 然后基于riskscore和临床指标预测临床风险评分：

data$riskscoreall <- predict(cox, type = "lp")

head(data)

data$riskscoreall <- ifelse(data$riskscoreall >= median(data$riskscoreall), "High", "Low")

data$riskscoreall <- factor(data$riskscoreall, levels = c("Low","High"))

riskScore_cli2 <- data

models=list( Tumor=cph(Surv(OS.time,OS)~Tumor,data=riskScore_cli2,x=TRUE,y=TRUE,surv=T),

Weight=cph(Surv(OS.time,OS)~Weight,data=riskScore_cli2,x=TRUE,y=TRUE,surv=T),

riskScore2=cph(Surv(OS.time,OS)~riskScore2,data=riskScore_cli2,x=TRUE,y=TRUE,surv=T),

Metastasis=cph(Surv(OS.time,OS)~Metastasis,data=riskScore_cli2,x=TRUE,y=TRUE,surv=T),

Age=cph(Surv(OS.time,OS)~Age,data=riskScore_cli2,x=TRUE,y=TRUE,surv=T),

riskscoreall=cph(Surv(OS.time,OS)~riskscoreall,data=riskScore_cli2,x=TRUE,y=TRUE,surv=T))

#time-cindex计算

times<-c(1,3,5,7)

cindex<-cindex(models,

formula=Surv(OS.time,OS)~1,

eval.times=times,

data=riskScore_cli2)

plot(cindex)

cindex$AppCindex

cindex_df<-data.frame(

Time=times,

do.call(cbind,cindex$AppCindex)

)

cindex_df

library(tidyr)

dat=pivot_longer(cindex_df,cols=2:7,

names_to="model",

values_to="cindex")

head(dat)

library(ggplot2)

ggplot(dat,aes(x=Time,y=cindex))+

geom_line(aes(color=model),linewidth=2)+

scale_color_brewer(palette="Set1")+

geom_hline(yintercept=0.5,linetype=4)+

ylim(0.4,1)+

labs(title="Time-dependentC-index",x="Time(years)",y="C-index")+

theme_bw()

}

####Fig5L####

{

#####05-决策曲线 ######

library(rms)

Stage <- lrm(OS~Stage,riskScore_cli2)

Metastasis <- lrm(OS~Metastasis,riskScore_cli2)

Nomogram <- lrm(OS~Stage+Metastasis+riskScore2,riskScore_cli2)

d_train <- dca(Stage,Metastasis,Nomogram)

ggplot(d_train)

}

#

Fig 7.R

# 免疫浸润

load("~/my_prepare/lfr_finally/Breastcancer_EMBOJ/emboj_预后模型/101机器学习/得到模型后续分析/棒棒糖图展示风险评分与免疫浸润的关系_24_8_29.Rdata")

correlation[1:4,1:3] # 要下面这个表达矩阵即可

# 这个是前面计算相关性得到的，往前翻一下correlation就能找到了

data=as.data.frame(correlation)

colnames(data)=c("Cell","cor","pvalue")

data[1:4,1:3]

# 4 读取数据并根据数据确定图像数据

#

# 4.1定义颜色

#定义圆圈颜色的函数

p.col = c('gold','pink','orange','LimeGreen','darkgreen')

fcolor = function(x,p.col){

color = ifelse(x>0.8,p.col[1],ifelse(x>0.6,p.col[2],ifelse(x>0.4,p.col[3],

ifelse(x>0.2,p.col[4], p.col[5])

)))

return(color)

}

# 4.2定义棒棒糖圈大小

#定义设置圆圈大小的函数

p.cex = seq(2.5, 5.5, length=5)

fcex = function(x){

x=abs(x)

cex = ifelse(x<0.1,p.cex[1],ifelse(x<0.2,p.cex[2],ifelse(x<0.3,p.cex[3],

ifelse(x<0.4,p.cex[4],p.cex[5]))))

return(cex)

}

# 2.3根据定义的函数进行上色

#根据pvalue定义圆圈的颜色

points.color = fcolor(x=data$pvalue,p.col=p.col)

data$points.color = points.color

points.cex = fcex(x=data$cor)

data$points.cex = points.cex

# 4.3.进行可视化

xlim = ceiling(max(abs(data$cor))*10)/10

pdf(file="../../../Breastcancer_EMBOJ/emboj_预后模型/101机器学习/得到模型后续分析/棒棒糖图_自己的数据_TNBC亚型数据.pdf", width=9, height=7)

pdf(file="../出图_24_9_3/fig7b_棒棒糖图_自己的数据_TNBC亚型数据.pdf", width=9, height=9)

# pdf(file="../Breastcancer_EMBOJ/new_analyse_24_4_17/ssgsea/棒棒糖图_自己的数据_TNBC亚型加了epi数据.pdf", width=9, height=7)

layout(mat=matrix(c(1,1,1,1,1,0,2,0,3,0),nc=2),width=c(8,2.2),heights=c(1,2,1,2,1))

par(bg="white",las=1,mar=c(5,18,2,4),cex.axis=1.5,cex.lab=2)

plot(1,type="n",xlim=c(-xlim,xlim),ylim=c(0.5,nrow(data)+0.5),xlab="Correlation Coefficient",ylab="",yaxt="n",yaxs="i",axes=F)

rect(par('usr')[1],par('usr')[3],par('usr')[2],par('usr')[4],col="#F5F5F5",border="#F5F5F5")

grid(ny=nrow(data),col="white",lty=1,lwd=2)

# 直到这里我们就将基本的图形框架绘制完了，但是需要直观地展示仍需添加一些东西

# 添加图形的线段

#绘制图形的线段

segments(x0=data$cor,y0=1:nrow(data),x1=0,y1=1:nrow(data),lwd=4)

#绘制图形的圆圈

points(x=data$cor,y = 1:nrow(data),col = data$points.color,pch=16,cex=data$points.cex)

#展示免疫细胞的名称

text(par('usr')[1],1:nrow(data),data$Cell,adj=1,xpd=T,cex=1.5)

# 展示每个免疫细胞的p值

#展示pvalue

pvalue.text=ifelse(data$pvalue<0.001,'<0.001',sprintf("%.03f",data$pvalue))

redcutoff_cor=0

redcutoff_pvalue=0.05

text(par('usr')[2],1:nrow(data),pvalue.text,adj=0,xpd=T,col=ifelse(abs(data$cor)>redcutoff_cor & data$pvalue<redcutoff_pvalue,"red","black"),cex=1.5)

axis(1,tick=F)

# 对图形添加图例

#绘制圆圈大小的图例

par(mar=c(0,4,3,4))

plot(1,type="n",axes=F,xlab="",ylab="")

legend("left",legend=c(0.1,0.2,0.3,0.4,0.5),col="black",pt.cex=p.cex,pch=16,bty="n",cex=2,title="abs(cor)")

#绘制圆圈颜色的图例

par(mar=c(0,6,4,6),cex.axis=1.5,cex.main=2)

barplot(rep(1,5),horiz=T,space=0,border=NA,col=p.col,xaxt="n",yaxt="n",xlab="",ylab="",main="pvalue")

axis(4,at=0:5,c(1,0.8,0.6,0.4,0.2,0),tick=F)

dev.off()

library(CIBERSORT)

# 运行

f = "../ssgsea/ciber_GSE58812.Rdata" #设置一个默认的结果保存文件

if(!file.exists(f)){

#devtools:: install_github ("Moonerss/CIBERSORT")

library(CIBERSORT)

lm22f = system.file("extdata", "LM22.txt", package = "CIBERSORT")

TME.results = cibersort(lm22f,

"../ssgsea/exp_cibersortUse.txt" , #存好的RNA-seq文件

perm = 1000, #迭代次数

QN = T) # # QN如果是芯片设置为T，如果是测序就设置为F，这边应该设置为F

save(TME.results,file = f)

}

load(f)

TME.results[1:4,1:4]

# B cells naive B cells memory Plasma cells T cells CD8

# GSM1419942 0.01064418 0 0.04222759 0.00000000

# GSM1419943 0.01363919 0 0.17402532 0.02386505

# GSM1419944 0.00000000 0 0.16042050 0.00000000

# GSM1419945 0.02107582 0 0.03571689 0.00000000

# 导出表格

load("~/my_prepare/lfr_finally/Breastcancer_EMBOJ/emboj_预后模型/ssgsea/ciber_GSE58812.Rdata")

write.csv(TME.results,file = "../出图_24_9_3/附表13_LM22_反卷积结果.csv")

# 添加风险分组

load("~/my_prepare/lfr_finally/Breastcancer_EMBOJ/emboj_预后模型/101机器学习/不用封装的跑/选定模型计算好的riskscore_24_8_29.Rdata")

Train_riskScore_cli[1:4,1:4]

rownames(Train_riskScore_cli)=Train_riskScore_cli[,1]

#

identical(rownames(Train_riskScore_cli),rownames(TME.results))

# [1] TRUE

forinfiltrate=cbind(Train_riskScore_cli,TME.results)

colnames(forinfiltrate)

# colnames(forinfiltrate)[40]="EMT_like_CAF"

forinfiltrate[1:2,1:16]

#

df=forinfiltrate

# 将数据框转化为长格式，假设 riskScore2 是高低风险的分组标签

df_melted <- melt(df, id.vars = "riskScore2",

measure.vars = c("B cells naive",

"B cells memory","Plasma cells","T cells CD8",

"T cells CD4 naive","T cells CD4 memory resting","T cells CD4 memory activated",

"T cells follicular helper","T cells regulatory (Tregs)","T cells gamma delta",

"NK cells resting","NK cells activated","Monocytes",

"Macrophages M0","Macrophages M1","Macrophages M2",

"Dendritic cells resting","Dendritic cells activated","Mast cells resting",

"Mast cells activated","Eosinophils","Neutrophils"))

ggplot(df_melted, aes(x = variable, y = value, fill = riskScore2)) +

geom_violin(trim = FALSE, scale = "width", width = 0.6, position = position_dodge(width = 0.9)) +

geom_boxplot(width = 0.1, position = position_dodge(0.9)) +

labs(x = "Cell Type", y = "Fraction", title = "") +

scale_fill_manual(values = c("#E95C59","#4DBBD5E5"), name = "Risk Group") +

# theme(axis.text.x = element_text(angle = 45, hjust = 1)) +

# theme_minimal()+

theme_classic() + # 设置为经典主题，没有背景

theme(

axis.text.x = element_text(angle = 45, hjust = 1), # 设置x轴标签垂直显示

# axis.text.x = element_text(size = 10), # 调整x轴标签字体大小

axis.title.x = element_text(size = 12), # 调整x轴标题字体大小

axis.title.y = element_text(size = 12), # 调整y轴标题字体大小

panel.spacing = unit(1, "lines") # 设置不同细胞类型之间的间隔

,panel.border = element_rect(colour = "gray", fill = NA, size = 1), # 添加黑色边框

axis.line.x = element_blank(), # 去掉x轴线

axis.line.y = element_blank(), # 去掉y轴线

axis.ticks.x = element_blank(), # 去掉x轴刻度线

axis.ticks.y = element_blank(), # 去掉y轴刻度线

axis.text.y = element_blank(), # 去掉y轴刻度标签

axis.title = element_blank() # 去掉轴标题

)

# TIDE小提琴图：

my_comparisons <- list( c("high", "low")) #添加比较分组

p1 <- ggviolin(tidy_res, x = 'riskGroup', y = 'TIDE', fill = 'riskGroup',

palette = c("#E95C59","#4DBBD5E5"),

width = 0.5,

add = 'boxplot', add.params = list(fill = "white")) +

stat_compare_means(comparisons = my_comparisons, label = "p.signif",

bracket.size=0.5, tip.length = 0.02, method = 't.test')+

theme(

# axis.text.x = element_text(angle = 90, hjust = 1), # 设置x轴标签垂直显示

# axis.text.x = element_text(size = 10), # 调整x轴标签字体大小

# axis.title.x = element_text(size = 12), # 调整x轴标题字体大小

# axis.title.y = element_text(size = 12), # 调整y轴标题字体大小

panel.border = element_rect(colour = "black", fill = NA, size = 1), # 添加黑色边框

axis.line.x = element_blank(), # 去掉x轴线

axis.line.y = element_blank(), # 去掉y轴线

# axis.ticks.x = element_blank(), # 去掉x轴刻度线

# axis.ticks.y = element_blank(), # 去掉y轴刻度线

# axis.text.y = element_blank(), # 去掉y轴刻度标签

# axis.title = element_blank() # 去掉轴标题

)

p1

# 抗肿瘤免疫循环 雷达图

library(ggradar)

ggradar(leida, background.circle.transparency = 0, group.colours = c("#E95C59","#4DBBD5E5"),

grid.min = -5,

grid.mid = 1,

grid.max = 5, # 默认最大为1，如果画图数据表中有大于1的就报错了，得改

values.radar = c(1,5,15)

)

#

Fig8.R

gene <- dd$gene## 转换

library(clusterProfiler)

gene = bitr(gene, fromType='SYMBOL', toType='ENTREZID', OrgDb='org.Hs.eg.db')## 去重

gene <- dplyr::distinct(gene,SYMBOL,.keep_all=TRUE)

gene_df <- data.frame(logFC=dd$logFC,

SYMBOL = dd$gene)

gene_df <- merge(gene_df,gene,by='SYMBOL')

head(gene_df)

# SYMBOL logFC ENTREZID

# 1 A1BG 0.06575477 1

# 2 A1BG-AS1 -0.02881001 503538

# 3 A1CF -0.05084698 29974

# 4 A2M 0.06471498 2

# 5 A2M-AS1 0.13557040 144571

# 6 A2ML1 0.10653129 144568

## geneList 三部曲

## 1.获取基因logFC

geneList <- gene_df$logFC

## 2.命名

names(geneList) = gene_df$ENTREZID

## 3.排序很重要

geneList = sort(geneList, decreasing = TRUE)

# 5.运行GSEA分析

#

# 从GESA(https://www.gsea-msigdb.org/gsea/downloads.jsp)的官网上，下载一个gmt文件

library(clusterProfiler)

## 读入hallmarks gene set，从哪来？ 这边要下载entrezid版本

hallmarks <- read.gmt('../../new_analyse_24_4_17/gsea/h.all.v2023.2.Hs.entrez.gmt')

# 需要网络

y <- GSEA(geneList,TERM2GENE =hallmarks)

# 作图看整体分布

### 看整体分布library(ggplot2)

dotplot(y,showCategory=12,split='.sign')+facet_grid(~.sign)

dotplot(y,showCategory=50,split='.sign')+facet_grid(~.sign)+

# 调整字体大小

theme(

axis.title = element_text(size = 14), # 坐标轴标题字体大小

axis.text = element_text(size = 12), # 坐标轴刻度标签字体大小

strip.text = element_text(size = 14), # 分面标签字体大小

legend.title = element_text(size = 12), # 图例标题字体大小

legend.text = element_text(size = 10) # 图例文本字体大小

)

# 6.特定通路作图

yd <- data.frame(y)

library(enrichplot)

gseaplot2(y,'HALLMARK_EPITHELIAL_MESENCHYMAL_TRANSITION',color = 'red',pvalue_table = T)

gseaplot2(y,'HALLMARK_TGF_BETA_SIGNALING',color = 'red',pvalue_table = T)

gseaplot2(y,'HALLMARK_NOTCH_SIGNALING',color = 'red',pvalue_table = T)

gseaplot2(y,'HALLMARK_WNT_BETA_CATENIN_SIGNALING',color = 'red',pvalue_table = T)

gseaplot2(y,'HALLMARK_PI3K_AKT_MTOR_SIGNALING',color = 'red',pvalue_table = T)

gseaplot2(y,'HALLMARK_GLYCOLYSIS',color = 'red',pvalue_table = T)

gseaplot2(y,'HALLMARK_OXIDATIVE_PHOSPHORYLATION',color = 'red',pvalue_table = T)

gseaplot2(y,'HALLMARK_OXIDATIVE_PHOSPHORYLATION',color = 'red',pvalue_table = T)

##对于多个通路绘制在一起：：：

pathway=c("HALLMARK_EPITHELIAL_MESENCHYMAL_TRANSITION","HALLMARK_TGF_BETA_SIGNALING","HALLMARK_WNT_BETA_CATENIN_SIGNALING","HALLMARK_PI3K_AKT_MTOR_SIGNALING","HALLMARK_NOTCH_SIGNALING","HALLMARK_GLYCOLYSIS","HALLMARK_OXIDATIVE_PHOSPHORYLATION")

gseaplot2(y,pathway,color = c('red',"blue"),pvalue_table = F)

#

save(yd,y,file = "../101机器学习/得到模型后续分析/GSEA_riskscore_用的是gse58812去计算_24_8_30.Rdata")

# hallmark相关性

load("~/my_prepare/lfr_finally/Breastcancer_EMBOJ/emboj_预后模型/101机器学习/不用封装的跑/选定模型计算好的riskscore_24_8_29.Rdata")

#

# 想这么做，就需要先通过gsva打分的方法计算每个样本各自通路的评分

load("~/my_prepare/lfr_finally/Breastcancer_EMBOJ/emboj_预后模型/101机器学习/得到模型后续分析/gse58812_重新清洗cli_24_8_29.Rdata")

exp_filter=exp

exp_filter[1:4,1:4]

# TCGA-BH-A0E0-11A TCGA-BH-A18V-11A TCGA-BH-A1FC-11A TCGA-E2-A158-11A

# RP11-368I23.2 0.5770281 0.00000000 0.33007291 0.93334894

# RP11-742D12.2 0.0000000 0.03483256 0.02550387 0.02258519

# RAB4B 3.0713037 3.90911352 3.50316793 2.76627551

# AC104183.2 0.0000000 0.00000000 0.00000000 0.00000000

expr=as.matrix(exp_filter)

# 2，获取目标基因集

# 根据自己的需要选择MSigDB数据库中的基因集

# 2.1 手动下载

#

# 进入http://www.gsea-msigdb.org/gsea/msigdb/index.jsp后选择需要下载的基因集，然后使用R读取下载好的gmt格式的文件。

# 下载50个肿瘤特征基因集合

# 多取几个基因集，取交集

# data1=clusterProfiler::read.gmt("../101机器学习/得到模型后续分析/h.all.v2024.1.Hs.symbols.gmt") # 返回的数据框

# geneset=GSA::GSA.read.gmt("new_analyse_24_4_17/h.all.v2023.2.Hs.symbols.gmt")

# geneset=mogsa::prepMsigDB("new_analyse_24_4_17/h.all.v2023.2.Hs.symbols.gmt") #list

geneset <- cogena::gmt2list("../101机器学习/得到模型后续分析/h.all.v2024.1.Hs.symbols.gmt") #list

# $HALLMARK_PROTEIN_SECRETION

# [1] "ABCA1" "ADAM10" "ANP32E" "AP1G1" "AP2B1" "AP2M1" "AP2S1" "AP3B1"

# [9] "AP3S1" "ARCN1" "ARF1" "ARFGAP3" "ARFGEF1" "ARFGEF2" "ARFIP1" "ATP1A1"

# [17] "ATP6V1B1" "ATP6V1H" "ATP7A" "BET1" "BNIP3" "CAV2" "CD63" "CLCN3"

# [25] "CLN5" "CLTA" "CLTC" "COG2" "COPB1" "COPB2" "COPE" "CTSC"

# [33] "DNM1L" "DOP1A" "DST" "EGFR" "ERGIC3" "GALC" "GBF1" "GLA"

# [41] "GNAS" "GOLGA4" "GOSR2" "ICA1" "IGF2R" "KIF1B" "KRT18" "LAMP2"

# [49] "LMAN1" "M6PR" "MAPK1" "MON2" "NAPA" "NAPG" "OCRL" "PAM"

# [57] "PPT1" "RAB14" "RAB22A" "RAB2A" "RAB5A" "RAB9A" "RER1" "RPS6KA3"

# [65] "SCAMP1" "SCAMP3" "SCRN1" "SEC22B" "SEC24D" "SEC31A" "SGMS1" "SH3GL2"

# [73] "SNAP23" "SNX2" "SOD1" "SSPN" "STAM" "STX12" "STX16" "STX7"

# [81] "TMED10" "TMED2" "TMX1" "TOM1L1" "TPD52" "TSG101" "TSPAN8" "USO1"

# [89] "VAMP3" "VAMP4" "VAMP7" "VPS45" "VPS4B" "YIPF6" "YKT6" "ZW10"

#

# $HALLMARK_REACTIVE_OXYGEN_SPECIES_PATHWAY

# [1] "ABCC1" "ATOX1" "CAT" "CDKN2D" "EGLN2" "ERCC2" "FES" "FTL" "G6PD"

# [10] "GCLC" "GCLM" "GLRX" "GLRX2" "GPX3" "GPX4" "GSR" "HHEX" "HMOX2"

# [19] "IPCEF1" "JUNB" "LAMTOR5" "LSP1" "MBP" "MGST1" "MPO" "MSRA" "NDUFA6"

# [28] "NDUFB4" "NDUFS2" "NQO1" "OXSR1" "PDLIM1" "PFKP" "PRDX1" "PRDX2" "PRDX4"

# [37] "PRDX6" "PRNP" "PTPA" "SBNO2" "SCAF4" "SELENOS" "SOD1" "SOD2" "SRXN1"

# [46] "STK25" "TXN" "TXNRD1" "TXNRD2"

#

# 2.2 msigdbr包

#

# 直接使用msigdbr包内置好的基因集，含有多个物种 以及 多个基因集，通过参数选择物种以及数据集，较为方便。推荐！

# library(msigdbr)

# msigdbr_species() #列出有的物种

#选择基因集合

# ?msigdbr

# human_KEGG = msigdbr(species = "Homo sapiens", #物种

# category = "C2",

# subcategory = "KEGG") %>%

# dplyr::select(gs_name,gene_symbol)#这里可以选择gene symbol或者ID

# human_KEGG_Set = human_KEGG %>% split(x = .$gene_symbol, f = .$gs_name)#list

#

# A：如果你的研究是其中的物种就可以无缝做GSEA 和 GSVA了。

#

# B：如果研究的物种不在其中，也可以自定义基因集，注意转为对应的形式。human_KEGG_Set 为基因集合的列表形式。

# 二 GSVA分析

#

# 1, GSVA分析

#

# 数据准备好后，加载GSVA包，一个gsva函数就可以得到GSVA的结果了。

library(GSVA)

gsva.kegg <- gsva(expr, gset.idx.list = geneset,

kcdf="Gaussian",

method = "gsva",

parallel.sz=1)

head(gsva.kegg)

# GSM1419942 GSM1419943 GSM1419944 GSM1419945

# HALLMARK_ADIPOGENESIS 0.319234180 -0.02995030 -0.12036696 0.12704217

# HALLMARK_ALLOGRAFT_REJECTION -0.373894018 0.09860410 -0.45872752 0.29896703

# HALLMARK_ANDROGEN_RESPONSE 0.375729281 0.13031497 -0.02026651 0.14336347

# HALLMARK_ANGIOGENESIS 0.073590349 0.15138661 -0.23981522 0.44516777

# HALLMARK_APICAL_JUNCTION -0.006224164 -0.13768544 -0.15815801 0.19611173

# HALLMARK_APICAL_SURFACE -0.100360665 -0.08079993 -0.29144559 -0.03340218

#

# 行为目标基因集，列为celltype ，数值为gsva分数。

#

# 这里需要注意，如果输入矩阵为log转化后的连续表达矩阵指则设置kcdf参数为"Gaussian"，如果是counts矩阵则设置kcdf为"Poisson"。

save(gsva.kegg,file = "../101机器学习/得到模型后续分析/gse数据_hallmark通路富集评分_24_8_30.Rdata")

# 2, 绘制热图

# 以结果的前50个绘制示例热图，可以自选择重点的通路

load("~/my_prepare/lfr_finally/Breastcancer_EMBOJ/emboj_预后模型/101机器学习/不用封装的跑/选定模型计算好的riskscore_24_8_29.Rdata")

#

# 想这么做，就需要先通过gsva打分的方法计算每个样本各自通路的评分

load("~/my_prepare/lfr_finally/Breastcancer_EMBOJ/emboj_预后模型/101机器学习/得到模型后续分析/gse58812_重新清洗cli_24_8_29.Rdata")

exp_filter=exp

exp_filter[1:4,1:4]

# TCGA-BH-A0E0-11A TCGA-BH-A18V-11A TCGA-BH-A1FC-11A TCGA-E2-A158-11A

# RP11-368I23.2 0.5770281 0.00000000 0.33007291 0.93334894

# RP11-742D12.2 0.0000000 0.03483256 0.02550387 0.02258519

# RAB4B 3.0713037 3.90911352 3.50316793 2.76627551

# AC104183.2 0.0000000 0.00000000 0.00000000 0.00000000

expr=as.matrix(exp_filter)

#

load("~/my_prepare/lfr_finally/Breastcancer_EMBOJ/emboj_预后模型/101机器学习/得到模型后续分析/gse数据_hallmark通路富集评分_24_8_30.Rdata")

library(pheatmap)

pheatmap(gsva.kegg[1:50,], show_colnames = T,

scale = "row",angle_col = "45",

cluster_row = T,cluster_col = T,

color = colorRampPalette(c("navy", "white", "firebrick3"))(50))

# 设置分组

ann_col =Train_riskScore_cli[,c(1,14)] #创建分组列

rownames(ann_col)=ann_col$sample

ann_col=as.data.frame(ann_col[-1])

colnames(ann_col)="Sample"

row.names(ann_col) = colnames(expr) #这一行必须有，否则会报错：Error in check.length("fill") : 'gpar' element 'fill' must not be length 0

ann_color = list(Sample = c(High="#E95C59",Low="#4DBBD5E5")) #定义分组颜色

pheatmap(gsva.kegg[1:50,], show_colnames = T,

scale = "row",angle_col = "45",

cluster_row = T,cluster_col = F,

color = colorRampPalette(c("navy", "white", "firebrick3"))(50),

annotation_col = ann_col, #表示是否对行、列进行注释，默认NA

annotation = NA, annotation_colors = ann_color #表示行注释及列注释的颜色，默认NA

)

pheatmap(gsva.kegg[1:50,],

scale = "row", #表示进行均一化的方向，值为 “row”, “column” 或者"none"

cluster_rows = T,cluster_cols = F, #cluster_rows表示仅对行聚类，cluster_cols表示仅对列聚类，值为TRUE或FALSE

cutree_rows = NA, cutree_cols = NA, #若进行了行/列聚类，根据行/列聚类数量分隔热图行,cutree_rows=num分割行，cutree_cols=num分割列

treeheight_row = 30, treeheight_col = 30, #若行、列聚类树高度调整

border_color = "grey60", #表示热图每个小的单元格边框的颜色，默认为 "grey60"

# cellwidth = 60, cellheight = 7.5, #表示单个单元格的宽度\高度，默认为 “NA”

display_numbers = F, #表示是否在单元格上显示原始数值或按照特殊条件进行区分标记

fontsize_number = 6, #表示热图上显示数字的字体大小

number_format = "%.2f", #表示热图单元格上显示的数据格式，“%.2f” 表示两位小数,“%.1e”表示科学计数法

number_color = "grey30", #表示热图单元格上显示的数据字体颜色

fontsize =10, fontsize_row = 6, fontsize_col = 10, #热图中字体大小、行、列名字体大小

show_rownames = T, show_colnames = T, #表示是否显示行名、列名

main = "Gene标题", #表示热图的标题名字

color = colorRampPalette(c("navy","white","firebrick3"))(100), #表示热图颜色,(100)表示100个等级

angle_col = "45", #表示列标签的角度

gaps_row = NULL, #仅在未进行行聚类时使用，表示在行方向上热图的隔断位置

gaps_col = c(1,2,3,4,5,6), #仅在未进行列聚类时使用，表示在列方向上热图的隔断位置

# annotation_row = ann_row,

annotation_col = ann_col, #表示是否对行、列进行注释，默认NA

annotation = NA, annotation_colors = ann_color, #表示行注释及列注释的颜色，默认NA

annotation_legend = TRUE, #表示是否显示注释的图例信息

annotation_names_row = TRUE, annotation_names_col = TRUE) #表示是否显示行、列注释的名称

# 为啥分组都分开了，我觉得是顺序的问题

# 修改行注释的顺序

# 使用order函数对Sample列进行排序，使得"High"在前，"Low"在后

ann_col$group=NA # 必不可少，只有一列下面这个代码会报错

ann_col <- ann_col[order(ann_col$Sample, decreasing = TRUE), ]

ann_col=as.data.frame(ann_col[-2])

# 根据修改好得注释得顺序，修改gsva.kegg顺序

# 使用match函数找出mat1行名在mat2中的位置

match_indices <- match(rownames(ann_col), colnames(gsva.kegg)) #使用第一个矩阵的行名作为参照，找出这些行名在第二个矩阵中的位置

# 使用这个索引顺序来重新排列mat2

gsva.kegg <- gsva.kegg[,match_indices]

#

identical(rownames(ann_col), colnames(gsva.kegg))

#

pheatmap(gsva.kegg[1:50,], show_colnames = F,

scale = "row",angle_col = "45",

cluster_row = T,cluster_col = F,

color = colorRampPalette(c("navy", "white", "firebrick3"))(50),

annotation_col = ann_col, #表示是否对行、列进行注释，默认NA

annotation = NA, annotation_colors = ann_color #表示行注释及列注释的颜色，默认NA

)

#

pheatmap(gsva.kegg[1:50,], show_colnames = F,

scale = "row",angle_col = "45",

cluster_row = T,cluster_col = F,

color = colorRampPalette(c("#4DBBD5E5","white","#E95C59"))(100),

annotation_col = ann_col, #表示是否对行、列进行注释，默认NA

annotation = NA, annotation_colors = ann_color #表示行注释及列注释的颜色，默认NA

)

#

save(ann_col,ann_color,gsva.kegg,file = "../出图_24_9_3/fig8d_hallmark_热图_画图需要.Rdata")

## 现在可以计算riskScore与通路得相关性了

rm(list=ls())

gc()

#

load("~/my_prepare/lfr_finally/Breastcancer_EMBOJ/emboj_预后模型/101机器学习/得到模型后续分析/gse数据_hallmark通路富集评分_24_8_30.Rdata")

#

load("~/my_prepare/lfr_finally/Breastcancer_EMBOJ/emboj_预后模型/101机器学习/不用封装的跑/选定模型计算好的riskscore_24_8_29.Rdata")

Train_riskScore_cli[1:4,1:4]

Train_riskScore_cli=Train_riskScore_cli[,c(1,13)]

rownames(Train_riskScore_cli)=Train_riskScore_cli$sample

Train_riskScore_cli=as.data.frame(Train_riskScore_cli[-1])

Train_riskScore_cli=t(Train_riskScore_cli)

Train_riskScore_cli=as.data.frame(Train_riskScore_cli)

#

identical(colnames(gsva.kegg),colnames(Train_riskScore_cli))

# [1] TRUE

exprSet=rbind(gsva.kegg,Train_riskScore_cli)

#

exprSet[49:51,1:4]

# GSM1419942 GSM1419943 GSM1419944 GSM1419945

# HALLMARK_WNT_BETA_CATENIN_SIGNALING -0.2861151 -0.01692967 0.2692770 -0.03754189

# HALLMARK_XENOBIOTIC_METABOLISM 0.2257636 -0.01212682 -0.2393317 0.26050552

# riskScore 1.9325508 1.47404423 3.1763459 1.04706084

exprSet=as.data.frame(exprSet)

class(exprSet[1,1])

# [1] "numeric"

#

exprSet1=exprSet

# 下面将行名去掉HALLMARK

rownames(exprSet)=str_split_fixed(rownames(exprSet),"_",n=2)[,2]

rownames(exprSet)[51]="riskScore"

# 2.写一个函数批量计算相关性

#

# 这个函数只要输入一个基因，他就会批量计算这个基因跟其他编码基因的相关

#

# 性，返回相关性系数和p值。

###对于有缺失值的基因，有效样本小于4会报错

batch_cor <- function(gene){

y <- as.numeric(exprSet[gene,])

rownames <- rownames(exprSet)

do.call(rbind,future_lapply(rownames, function(x){

dd <- cor.test(as.numeric(exprSet[x,]),y,type='spearman')

data.frame(gene=gene,mRNAs=x,cor=dd$estimate,p.value=dd$p.value )

}))

}

###这是修改的代码 加一个判断 样本量<10的就不要了吧

batch_cor <- function(gene){

rownames <- rownames(exprSet)

do.call(rbind,future_lapply(rownames, function(x){

xy <- exprSet[c(gene,x),]

xy <- t(xy) %>% na.omit() %>% as.data.frame()

if (nrow(xy)>10){

dd <- cor.test(as.numeric(xy[,1]),as.numeric(xy[,2]),type='spearman')

data.frame(gene=gene,mRNAs=x,cor=dd$estimate,p.value=dd$p.value )

}

}))

}

# 3.并行化运行函数

#

# 以riskScore这个基因为例

library(future.apply)

# plan(multiprocess)

system.time(dd <- batch_cor('riskScore'))

# system.time(dd <- batch_cor('MFAP2'))

# 绘制热图

exprSet_t=t(exprSet)

corr <- cor(exprSet_t)

library(corrplot)

# 默认绘图样式

corrplot(corr)

col2 <- rev(COL2('RdBu', 100)) # 生成调色板后使用 rev() 函数来颠倒顺序

col2 = colorRampPalette(c("#4DBBD5E5","white","#E95C59"))(200)

corrplot(corr, method = c('pie'),

type = c('upper'),

col = col2, # 设置一个连续的

outline = 'white',

# order = c('AOE'),

diag = TRUE,

tl.cex = 0.5, #对角线文字大小

tl.col = 'black', #对角线文字颜色

tl.pos = 'td' # d仅在对角线显示文本标签

# ,bg = "lightblue" # 设置图标背景颜色

# ,mar = c(0,0,0,0)

)

save(exprSet,exprSet_t,corr,col2,file = "../出图_24_9_3/fig8e_hallmark相关性热图_表格.Rdata")

#### 计算hallmark上下调比较显著的通路与预后的关系。

rm(list=ls())

gc()

#

load("~/my_prepare/lfr_finally/Breastcancer_EMBOJ/emboj_预后模型/101机器学习/Mime1包/mydata/GSE58812_不分割log的清洗好的data.Rdata")

Train$OS.time=as.numeric(Train$OS.time)

Train=Train %>% filter(OS.time>200)

Train[1:4,1:4]

#

Train=Train[,c(1:3)]

head(Train)

# ID OS.time OS

# GSM1419942 GSM1419942 1520 1

# GSM1419943 GSM1419943 1281 1

# GSM1419944 GSM1419944 1066 1

# GSM1419945 GSM1419945 1050 1

# GSM1419946 GSM1419946 422 1

# GSM1419947 GSM1419947 2081 0

# 取hallmark的GSVA富集分析结果

load("~/my_prepare/lfr_finally/Breastcancer_EMBOJ/emboj_预后模型/101机器学习/得到模型后续分析/gse数据_hallmark通路富集评分_24_8_30.Rdata")

gsva=as.data.frame(t(gsva.kegg))

gsva[1:4,1:4]

# HALLMARK_ADIPOGENESIS HALLMARK_ALLOGRAFT_REJECTION

# GSM1419942 0.3192342 -0.3738940

# GSM1419943 -0.0299503 0.0986041

# GSM1419944 -0.1203670 -0.4587275

# GSM1419945 0.1270422 0.2989670

identical(rownames(Train),rownames(gsva))

#

aimplot=cbind(Train,gsva)

#

aimplot$OS.time=as.numeric(aimplot$OS.time)

aimplot$OS=as.numeric(aimplot$OS)

#

aimplot$Alveolar_quartile=ifelse(aimplot$HALLMARK_ANGIOGENESIS>median(aimplot$HALLMARK_ANGIOGENESIS),'high','low')

aimplot$Alveolar_quartile=ifelse(aimplot$HALLMARK_EPITHELIAL_MESENCHYMAL_TRANSITION>median(aimplot$HALLMARK_EPITHELIAL_MESENCHYMAL_TRANSITION),'high','low')

aimplot$Alveolar_quartile=ifelse(aimplot$HALLMARK_INFLAMMATORY_RESPONSE>median(aimplot$HALLMARK_INFLAMMATORY_RESPONSE),'high','low')

aimplot$Alveolar_quartile=ifelse(aimplot$HALLMARK_NOTCH_SIGNALING>median(aimplot$HALLMARK_NOTCH_SIGNALING),'high','low')

aimplot$Alveolar_quartile=ifelse(aimplot$HALLMARK_ALLOGRAFT_REJECTION>median(aimplot$HALLMARK_ALLOGRAFT_REJECTION),'high','low')

aimplot$Alveolar_quartile=ifelse(aimplot$HALLMARK_INTERFERON_ALPHA_RESPONSE>median(aimplot$HALLMARK_INTERFERON_ALPHA_RESPONSE),'high','low')

aimplot$Alveolar_quartile=ifelse(aimplot$HALLMARK_INTERFERON_GAMMA_RESPONSE>median(aimplot$HALLMARK_INTERFERON_GAMMA_RESPONSE),'high','low')

aimplot$Alveolar_quartile=ifelse(aimplot$HALLMARK_GLYCOLYSIS>median(aimplot$HALLMARK_GLYCOLYSIS),'high','low')

aimplot$Alveolar_quartile=ifelse(aimplot$HALLMARK_MYOGENESIS>median(aimplot$HALLMARK_MYOGENESIS),'high','low')

aimplot$Alveolar_quartile=ifelse(aimplot$HALLMARK_CHOLESTEROL_HOMEOSTASIS>median(aimplot$HALLMARK_CHOLESTEROL_HOMEOSTASIS),'high','low')

aimplot$Alveolar_quartile=ifelse(aimplot$HALLMARK_HYPOXIA>median(aimplot$HALLMARK_HYPOXIA),'high','low')

aimplot$Alveolar_quartile=ifelse(aimplot$HALLMARK_COAGULATION>median(aimplot$HALLMARK_COAGULATION),'high','low')

aimplot$Alveolar_quartile=ifelse(aimplot$HALLMARK_APICAL_JUNCTION>median(aimplot$HALLMARK_APICAL_JUNCTION),'high','low')

aimplot$Alveolar_quartile=ifelse(aimplot$HALLMARK_HEDGEHOG_SIGNALING>median(aimplot$HALLMARK_HEDGEHOG_SIGNALING),'high','low')

aimplot$Alveolar_quartile=ifelse(aimplot$HALLMARK_ESTROGEN_RESPONSE_EARLY>median(aimplot$HALLMARK_ESTROGEN_RESPONSE_EARLY),'high','low')

aimplot$Alveolar_quartile=ifelse(aimplot$HALLMARK_ESTROGEN_RESPONSE_LATE>median(aimplot$HALLMARK_ESTROGEN_RESPONSE_LATE),'high','low')

aimplot$Alveolar_quartile=ifelse(aimplot$HALLMARK_IL6_JAK_STAT3_SIGNALING>median(aimplot$HALLMARK_IL6_JAK_STAT3_SIGNALING),'high','low')

aimplot$Alveolar_quartile=ifelse(aimplot$HALLMARK_COMPLEMENT>median(aimplot$HALLMARK_COMPLEMENT),'high','low')

aimplot$Alveolar_quartile=ifelse(aimplot$HALLMARK_KRAS_SIGNALING_UP>median(aimplot$HALLMARK_KRAS_SIGNALING_UP),'high','low')

aimplot$Alveolar_quartile=ifelse(aimplot$HALLMARK_TNFA_SIGNALING_VIA_NFKB>median(aimplot$HALLMARK_TNFA_SIGNALING_VIA_NFKB),'high','low')

aimplot$Alveolar_quartile=ifelse(aimplot$HALLMARK_INFLAMMATORY_RESPONSE>median(aimplot$HALLMARK_INFLAMMATORY_RESPONSE),'high','low')

aimplot$Alveolar_quartile=ifelse(aimplot$HALLMARK_KRAS_SIGNALING_DN>median(aimplot$HALLMARK_KRAS_SIGNALING_DN),'high','low')

aimplot$Alveolar_quartile=ifelse(aimplot$HALLMARK_IL2_STAT5_SIGNALING>median(aimplot$HALLMARK_IL2_STAT5_SIGNALING),'high','low')

table(aimplot$Alveolar_quartile)

NK_OS <- survfit(Surv(OS.time,OS)~Alveolar_quartile,data = aimplot)

ggsurvplot(NK_OS,pval = T,risk.table = T,surv.median.line = 'hv',

title='Overall survival',xlab='Days')

library(ggsci)

palette = "npg" # 使用 Nature 期刊风格配色

ggsurvplot(NK_OS, data = aimplot,

conf.int = TRUE, pval = TRUE,

surv.median.line = "hv",

# risk.table = TRUE,

# risk.table.height = 0.25, # 调整风险表高度

# risk.table.col = "strata", # 风险表按分层显示颜色

palette = "npg",

legend.labs=c("High","Low"), #标签

legend.title="RiskScore",

title="HALLMARK_IL2_STAT5_SIGNALING", #标题

ylab="Overall survival",xlab = " Time (Days)", #更改横纵坐标

censor.shape = 124,censor.size = 2, #删失点的形状和大小 break.x.by = 720#横坐标间隔

ggtheme = theme_minimal()+ # 使用干净的主题

theme(plot.title = element_text(hjust = 0.5),# 标题居中

panel.grid = element_blank(), # 移除背景网格线

axis.line = element_line(color = "black", size = 0.5) # 添加x轴和y轴线条

)

)

#

Fig9.R

# cellchat same as fig3

# KM

load("~/my_prepare/lfr_finally/Breastcancer_EMBOJ/emboj_预后模型/wgcna/new/最相关模块基因.Rdata")

# 过滤样本

load("~/my_prepare/lfr_finally/Breastcancer_EMBOJ/emboj_预后模型/101机器学习/Mime1包/mydata/GSE58812_不分割log的清洗好的data.Rdata")

Train$OS.time=as.numeric(Train$OS.time)

Train=Train %>% filter(OS.time>200)

#

load("~/my_prepare/lfr_finally/Breastcancer_EMBOJ/emboj_预后模型/101机器学习/自己代码跑/traindataset.Rdata")

TrainDataset$OS.time=as.numeric(TrainDataset$OS.time)

TrainDataset=TrainDataset %>% filter(OS.time>200)

load("~/my_prepare/lfr_finally/Breastcancer_EMBOJ/emboj_预后模型/101机器学习/自己代码跑/testDatasetGeo135565.Rdata")

testDatasetGeo135565$OS.time=as.numeric(testDatasetGeo135565$OS.time)

testDatasetGeo135565=testDatasetGeo135565 %>% filter(OS.time>200)

#

Train$OS.time=as.numeric(Train$OS.time)

Train$OS=as.numeric(Train$OS)

#

TrainDataset$OS.time=as.numeric(TrainDataset$OS.time)

TrainDataset$OS=as.numeric(TrainDataset$OS)

testDatasetGeo135565$OS.time=as.numeric(testDatasetGeo135565$OS.time)

testDatasetGeo135565$OS=as.numeric(testDatasetGeo135565$OS)

library(survival)

library(survminer)

#

Train[1:4,1:4]

# ID OS.time OS A1BG

# GSM1419942 GSM1419942 1520 1 8.591714

# GSM1419943 GSM1419943 1281 1 7.533359

# GSM1419944 GSM1419944 1066 1 7.366889

# GSM1419945 GSM1419945 1050 1 7.574151

exp_meta_for_survival=Train

# 按基因表达值，计算二分类

exp_meta_for_survival$SULF1_Group=ifelse(exp_meta_for_survival$WFDC1>median(exp_meta_for_survival$WFDC1),'high','low')

exp_meta_for_survival$SULF1_Group=ifelse(exp_meta_for_survival$UQCRQ>median(exp_meta_for_survival$UQCRQ),'high','low')

exp_meta_for_survival$SULF1_Group=ifelse(exp_meta_for_survival$ISCU>median(exp_meta_for_survival$ISCU),'high','low')

exp_meta_for_survival$SULF1_Group=ifelse(exp_meta_for_survival$FN1>median(exp_meta_for_survival$FN1),'high','low')

exp_meta_for_survival$SULF1_Group=ifelse(exp_meta_for_survival$TBX2>median(exp_meta_for_survival$TBX2),'high','low')

exp_meta_for_survival$SULF1_Group=ifelse(exp_meta_for_survival$NOTCH3>median(exp_meta_for_survival$NOTCH3),'high','low')

exp_meta_for_survival$SULF1_Group=ifelse(exp_meta_for_survival$C1S>median(exp_meta_for_survival$C1S),'high','low')

exp_meta_for_survival$SULF1_Group=ifelse(exp_meta_for_survival$JUN>median(exp_meta_for_survival$JUN),'high','low')

exp_meta_for_survival$SULF1_Group=ifelse(exp_meta_for_survival$PLS3>median(exp_meta_for_survival$PLS3),'high','low')

table(exp_meta_for_survival$SULF1_Group)

# sfit <- survfit(Surv(OS.time, OS)~SULF1_Group, data=exp_meta_for_survival)

# print(sfit)

# ggsurvplot(sfit, conf.int=F, pval=TRUE)

#

fit <- survfit(Surv(OS.time, OS) ~ SULF1_Group, data = exp_meta_for_survival)

ggsurvplot(fit, data = exp_meta_for_survival,

conf.int = TRUE, pval = TRUE,

surv.median.line = "hv",

risk.table = TRUE, palette = "hue")

library(ggsci)

palette = "npg" # 使用 Nature 期刊风格配色

ggsurvplot(fit, data = exp_meta_for_survival,

conf.int = TRUE, pval = TRUE,

surv.median.line = "hv",

# risk.table = TRUE,

# risk.table.height = 0.25, # 调整风险表高度

# risk.table.col = "strata", # 风险表按分层显示颜色

palette = "npg",

legend.labs=c("High","Low"), #标签

legend.title="RiskScore",

title="PLS3", #标题

ylab="Overall survival",xlab = " Time (Days)", #更改横纵坐标

censor.shape = 124,censor.size = 2, #删失点的形状和大小 break.x.by = 720#横坐标间隔

ggtheme = theme_minimal()+ # 使用干净的主题

theme(plot.title = element_text(hjust = 0.5),# 标题居中

panel.grid = element_blank(), # 移除背景网格线

axis.line = element_line(color = "black", size = 0.5) # 添加x轴和y轴线条

)

)

# 韦恩图

# TCGA差异基因 # log2fc>1,<-1

load("~/my_prepare/lfr_finally/Breastcancer_EMBOJ/emboj_预后模型/DEG_analyse/step5_差异分析矩阵和结果.Rdata")

# 导出表格

write.csv(expr_for_diff,file = "../出图_24_9_3/附表18_TCGA癌跟癌旁差异分析.csv")

deg_tcga=expr_for_diff[expr_for_diff$change!="nochange",]

tcga=deg_tcga$row

# GSE76250 # allDiff$logFC > 1 & allDiff$adj.P.Val < 0.05

load("~/my_prepare/lfr_finally/Breastcancer_EMBOJ/emboj_预后模型/geo数据/gse76250_癌跟癌旁分组差异分析_24_9_12.Rdata")

# 导出表格

write.csv(allDiff,file = "../出图_24_9_3/附表19_gse76250差异分析.csv")

deg_gse76250=allDiff[allDiff$type!="not-sig",]

gse76250=deg_gse76250$gene

# 再加上那9个基因取交集

gene=c("UQCRQ","ISCU","FN1","TBX2","NOTCH3","WFDC1","C1S","JUN","PLS3")

# 绘制韦恩图

library(VennDiagram)

# 绘制 3 集合的韦恩图

venn.plot <- venn.diagram(

x = list(GSE76250 = gse76250, TCGA = tcga, Modelgene = gene), # 数据

category.names = c("GSE76250", "TCGA", "Modelgene"), # 集合标签

filename = NULL, # 不保存到文件，直接在R绘图窗口中显示

col = "transparent", # 边框颜色

fill = c("#53A85F", "#E95C59", "#57C3F3"), # 填充颜色

# alpha = 0.5, # 透明度

cex = 2, # 字体大小

cat.cex = 2, # 标签字体大小

cat.pos = c(-20, 20, 0), # 调整标签的位置

cat.dist = c(0.1, 0.1, 0.1) # 调整标签与图的距离

)

# 显示韦恩图

grid.draw(venn.plot)

dev.off()

#
