# Supplementary material for: Construction of a stromal cell-related prognostic signature based on a 101-combination machine learning framework for predicting prognosis and immunotherapy response in triple-negative breast cancer
Source: Front Immunol. 2025 May 14;16:1544348. doi: 10.3389/fimmu.2025.1544348 (PMC12116347; doi:10.3389/fimmu.2025.1544348)
Supplement: Supplementary file 3 [file Image1.pdf]

Supplementary Material

1.1 Supplementary Figures

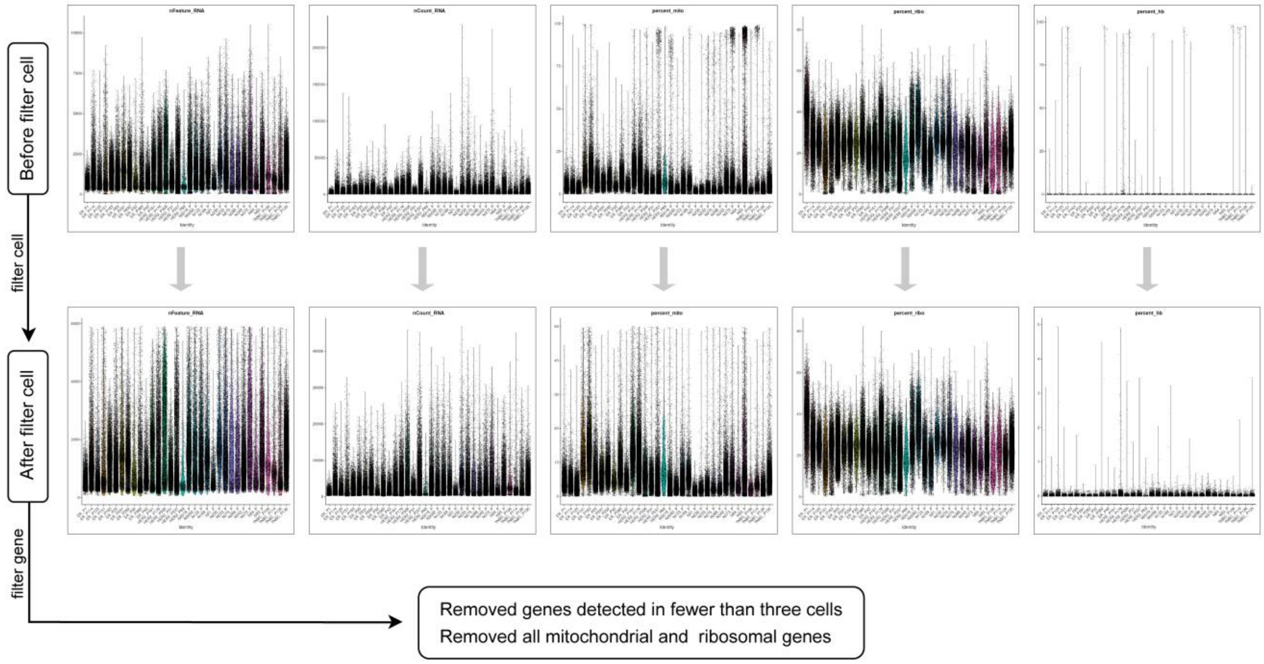

Supplementary Figure 1. Workflow of Cell and Gene Filtering in Single-Cell RNA Sequencing Data. This figure illustrates the quality control process of scRNA-seq data before and after cell and gene filtering, including the distribution of nFeature_RNA, nCount_RNA, percent_mito, percent_ribo, and percent_hb. The first and second rows respectively show sequencing quality parameters of different samples before and after filtering. The final row further demonstrates the gene filtering process.

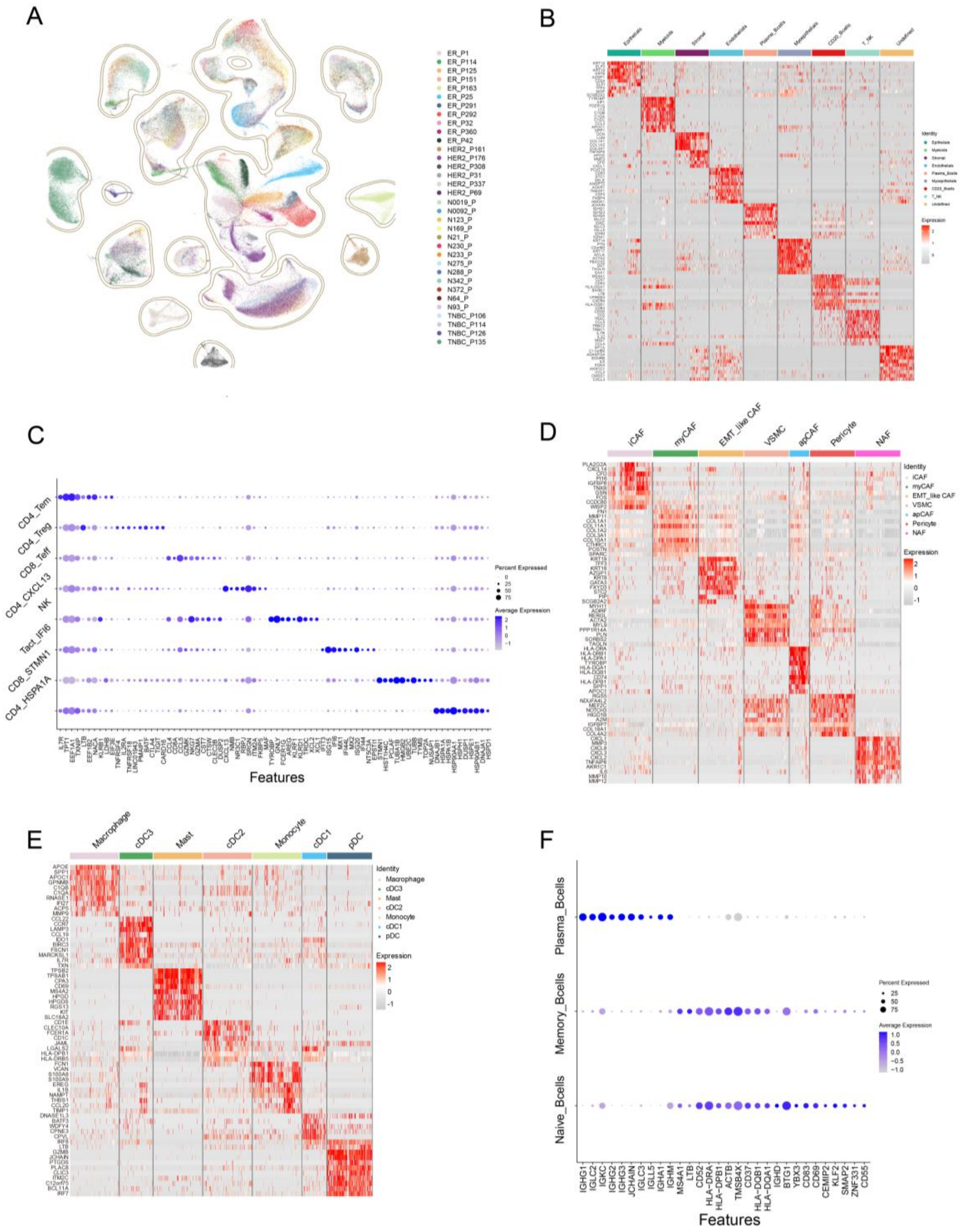

Supplementary Figure 2. Cellular origins and marker genes of cell populations. (A) UMAP plot of single-cell transcriptomic data across multiple breast cancer subtypes, including ER+, HER2+, and TNBC tissues. Each color represents a specific patient sample. (B) Single-cell expression heatmap of representative genes across major cell types. (C)Dot plot showing the expression levels of marker genes for different T cell subsets. (D) Heatmap illustrating the expression patterns of marker genes for fibroblast subsets. (E) Heatmap of marker gene expression for Myeloid cell subsets. (F) Dot plot demonstrating the expression of marker genes for B cell subsets.

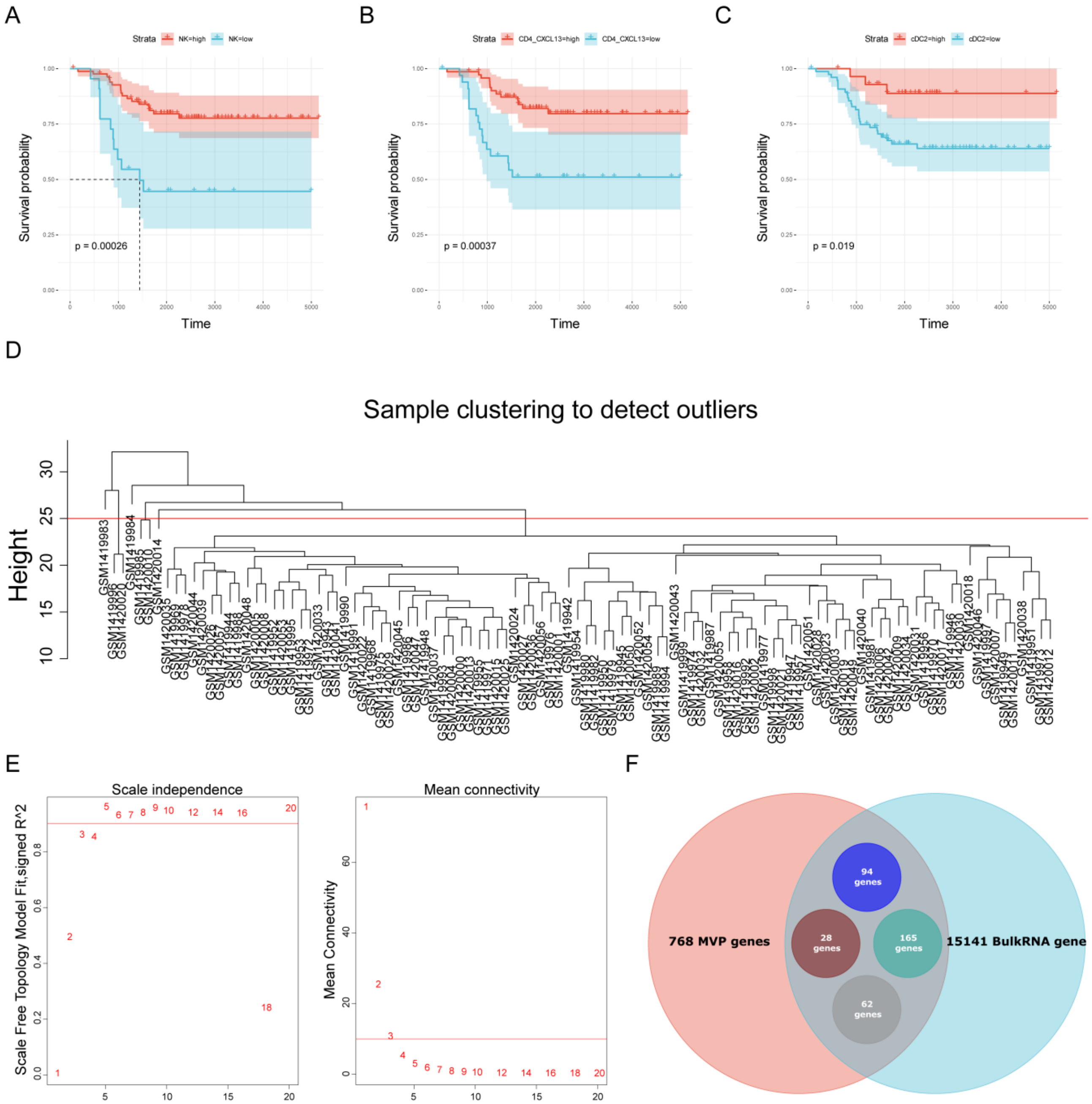

Supplementary Figure 3. Prognosis of preferentially distribution cell clusters in TNBC and sample clustering with soft-threshold selection in WGCNA analysis. (A-C) Kaplan-Meier curves illustrating the prognostic significance of NK cell, CD4_CXCL13 cell and cDC2 cell infiltration. (D) Sample clustering dendrogram used to identify and exclude outlier samples. (E) Scale-free topology model fit (R^2) and mean connectivity plots used to determine soft-thresholding power for constructing a weighted gene co-expression network. (F) Venn Diagram Analysis of MVP Genes and Bulk RNA Genes. The left red region represents the MVP gene set identified from scRNA-seq data analysis, while the right blue region represents highly variable genes in Bulk RNA-seq data. The overlapping section in the middle shows the intersection of MVP genes from scRNA-seq and highly variable genes in Bulk RNA data, with different colors indicating distinct co-expression gene modules identified by WGCNA.

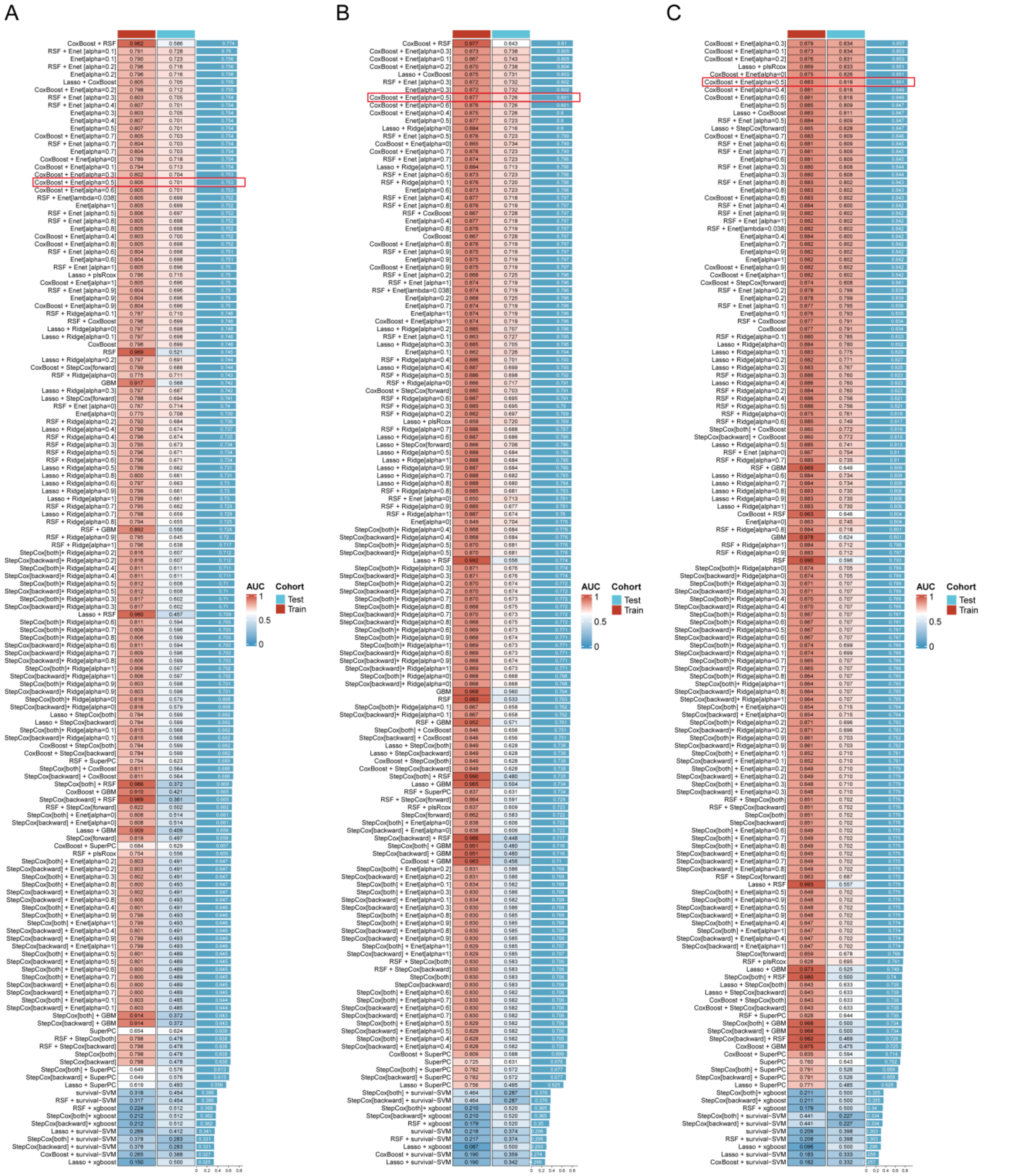

Supplementary Figure 4. AUC Evaluation of Different Model Combinations at 3, 5, and 7 Years. (A-C) This figure presents the AUC evaluation results of 101 predictive models for survival prediction at 3, 5, and 7 years, comparing the discriminative ability of different models. The Y-axis represents the model names, with each row corresponding to a specific model combination. The Cohort distinguishes the dataset sources used in the models (Test and Train). The AUC value is used to assess the survival prediction performance of the models, where values closer to 1.0 indicate a stronger ability to distinguish between high-risk and low-risk patients.

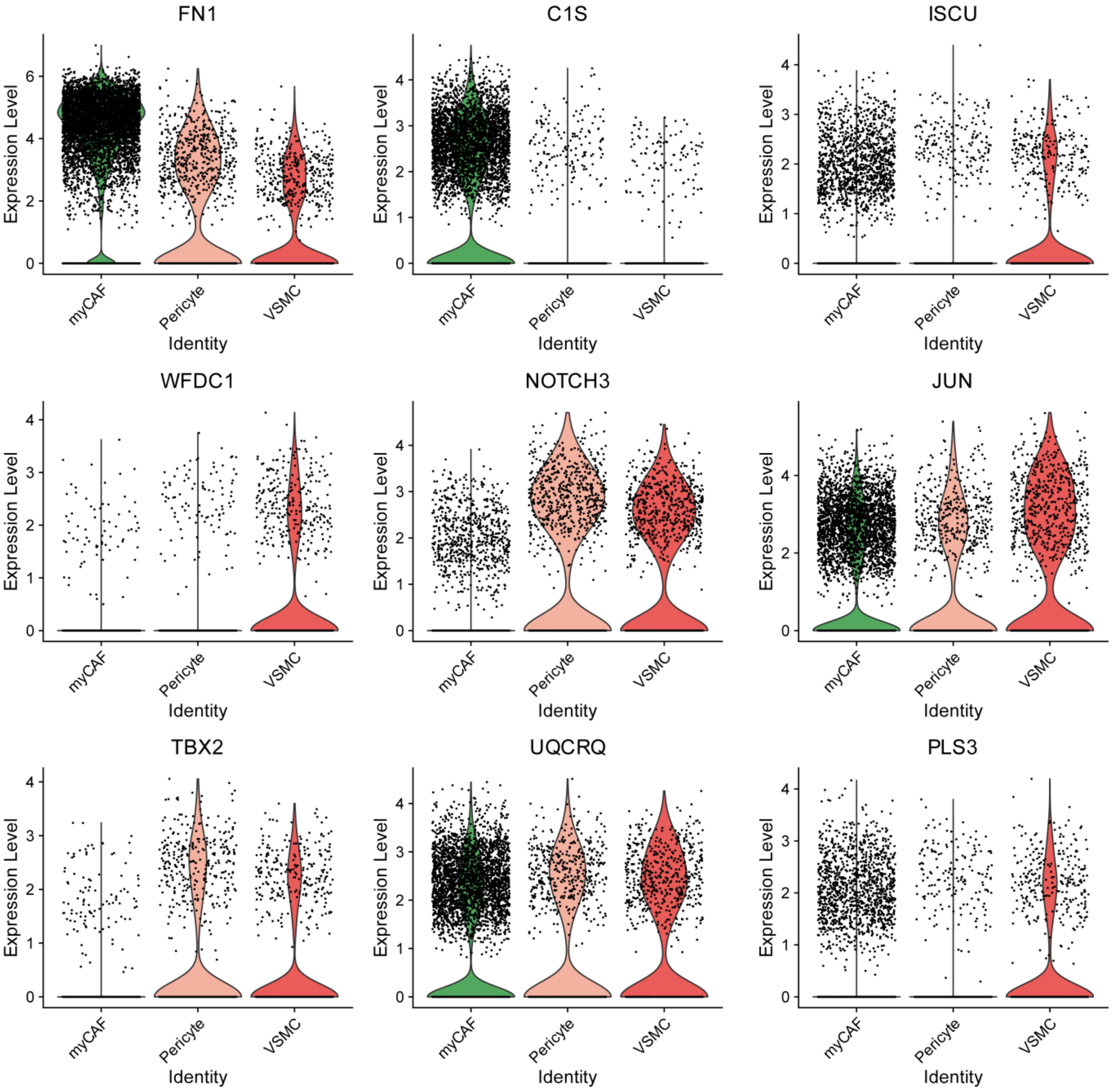

Supplementary Figure 5. Expression distribution of model genes across myCAF, Pericyte, and VSMC cell populations. Violin plots illustrate the expression levels of model genes (FN1, C1S, ISCU, WFDC1, NOTCH3, JUN, TBX2, UQCRQ, and PLS3) across three stromal cell populations. The X-axis lists different stromal cell types, and the Y-axis represents gene expression levels. Each dot represents a single cell.

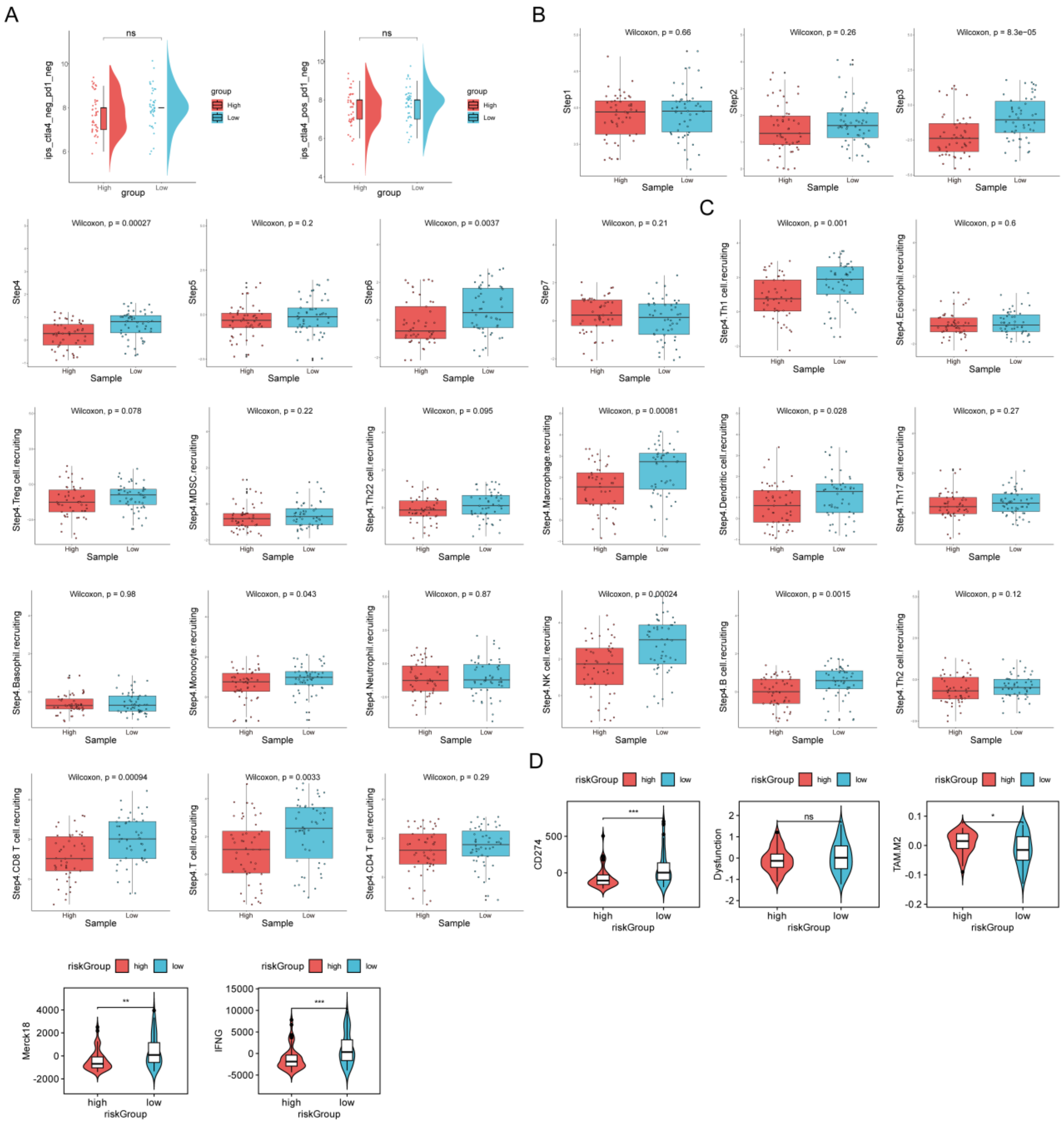

Supplementary Figure 6. Immunotherapy response and anti-cancer immune cycle in high- and low-risk groups. (A) The IPS score of ips_ctla4_neg_pd1_neg and ips_ctla4_pos _pd1_neg compared across high- and low-risk groups. (B) Boxplots showing the differences in the activity of the seven-step anti-cancer immunity cycle between high- and low-risk groups. (C) Boxplots depicting the difference in immune cell recruitment capabilities between MVPRS subgroups. (D) Violin plots depicting CD274 scores, Dysfunction scores, TAM.M2 scores, Merck18 scores, and IFNG scores between high- and low-risk groups.

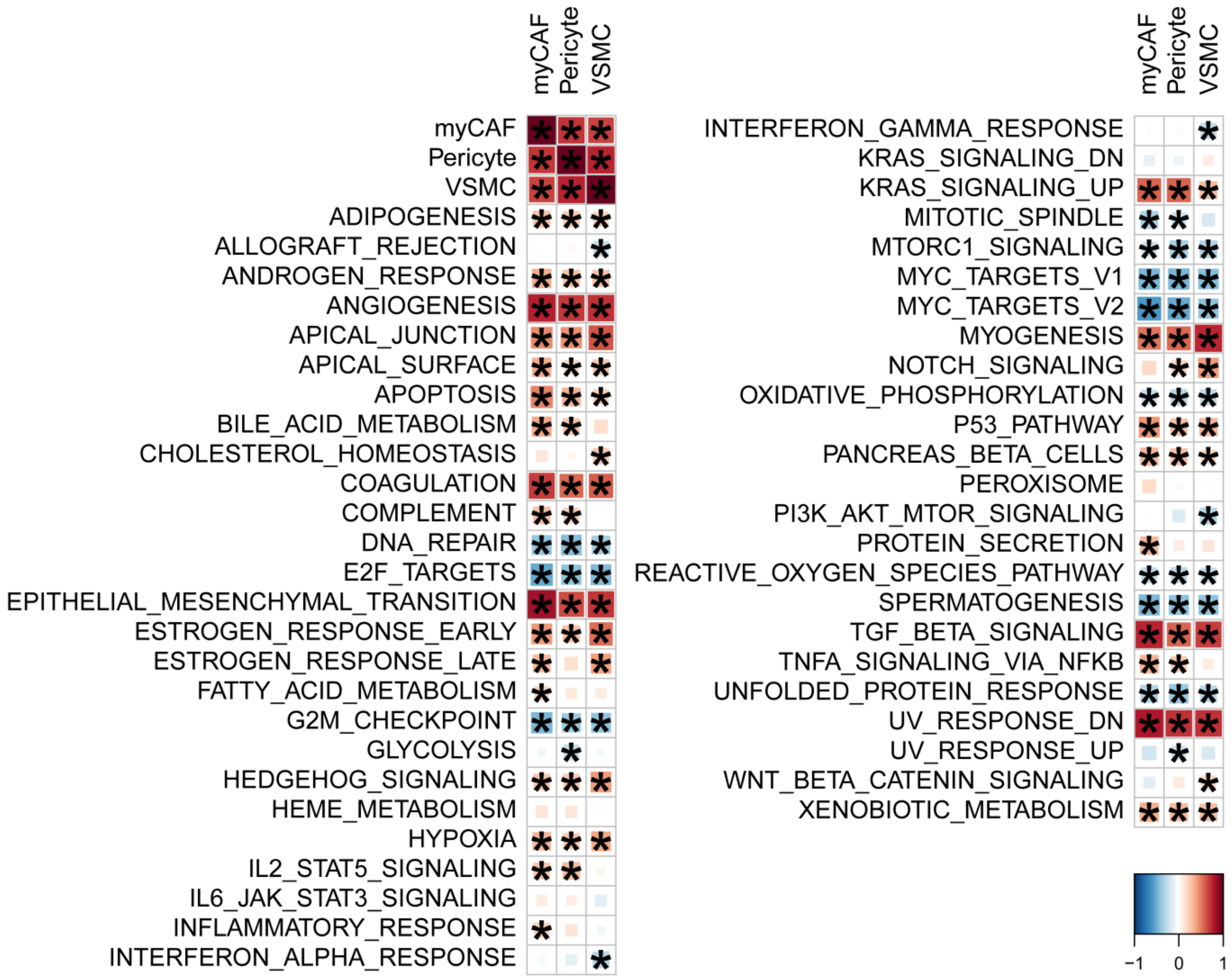

Supplementary Figure 7. Correlation analysis between stromal cell gene set scores and Hallmark pathway scores. (A) This figure illustrates the correlation between myCAF, Pericyte, and VSMC gene set scores and Hallmark pathway scores, calculated using the GSVA method. All gene sets were sourced from the MsigDB database. The color gradient represents the magnitude of the correlation coefficient, with red indicating positive correlation and blue indicating negative correlation. Statistical significance is indicated by symbols (*).
